# Virulence and transcriptome profile of multidrug-resistant *Escherichia coli* from chicken

**DOI:** 10.1038/s41598-017-07798-1

**Published:** 2017-08-21

**Authors:** Hafiz I. Hussain, Zahid Iqbal, Mohamed N. Seleem, Deyu Huang, Adeel Sattar, Haihong Hao, Zonghui Yuan

**Affiliations:** 1 MOA Laboratory for Risk Assessment of Quality and Safety of Livestock and Poultry Products, Huazhong Agricultural University, Wuhan, China; 2 National Reference Laboratory of Veterinary Drug Residues (HZAU) and MOA Key Laboratory for Detection of Veterinary Drug Residues, Huazhong Agricultural University, Wuhan, China; 3 Department of Comparative Pathobiology, College of Veterinary Medicine, Purdue University, West Lafayette, Indiana, USA; 4 University College of Veterinary and Animal Sciences, Present Address: The Islamia University of Bahawalpur, Bahawalpur, Pakistan

## Abstract

Numerous studies have examined the prevalence of pathogenic *Escherichia coli* in poultry and poultry products; however, limited data are available regarding their resistance- and virulence-associated gene expression profiles. This study was designed to examine the resistance and virulence of poultry *E. coli* strains *in vitro* and *in vivo* via antibiotic susceptibility, biofilm formation and adhesion, and invasion and intracellular survivability assays in Caco-2 and Raw 264.7 cell lines as well as the determination of the median lethal dose in two-day old chickens. A clinical pathogenic multidrug-resistant isolate, *E. coli* 381, isolated from broilers, was found to be highly virulent in cell culture and 1000-fold more virulent in a chicken model than other strains; accordingly, the isolate was subsequently selected for transcriptome analysis. The comparative gene expression profile of MDR *E. coli* 381 and the reference human strain *E. coli* ATCC 25922 was completed with Illumina HiSeq. 2500 transcriptome analysis. Differential gene expression analysis indicates that there are multiple pathways involved in the resistance and virulence of this highly virulent strain. The results garnered from this study provide critical information about the highly virulent MDR *E. coli* strain of poultry origin and warrant further investigation due to its significant threat to public health.

## Introduction

*Escherichia coli* is a Gram-negative bacterium that displays a wide range of genomic diversity. *E. coli* is the causative agent of many critical poultry diseases, including airsacculitis, pericarditis, peritonitis, salpingitis, polyserositis, colisepticemia, diarrhea, synovitis, osteomyelitis, and swollen head syndrome. In addition, in mammals it causes neonatal meningitis, septicemia, pyometra, mastitis, urinary tract infection, sepsis, diarrhea, all of which are complicated by other syndromes^1^. These diseases are collectively called colibacillosis. Colibacillosis is blamed for detrimental economic losses in the poultry sector in the form of morbidity, mortality, less body weight gain, carcass contamination, and recalled products^2, 3^.

The widespread use of antibiotics in the poultry industry to promote growth and prevent microbial infections has led to the emergence of MDR *E. coli* strains^4^. These MDR strains are easily transmitted to humans by direct or indirect contact^5^. MDR *E. coli* of poultry origin are highly prevalent in China and also present globally^6, 7^. The increasing prevalence of MDR and the virulent characteristics of poultry *E. coli* need to be better understood at the genetic bases. Therefore, RNA-Seq based transcriptomic profiling of selected poultry *E. coli* will be used here to identify the contributing factors to the virulence and resistance of the strain.

The acidic environment of most of the digestive tract provides a natural obstacle against infections of pathogenic bacteria. However, many pathogenic bacteria, such as enteric *E. coli*, have developed resistance against an acidic environment. Acquiring acid-resistant genes, such as *hdeA, hdeB, hdeD*, and *asr*, enable these bacteria to survive and colonize in acidic environments and cause infections under extremely low pH conditions. Thus, the acquisition of acid-resistant genes results in bacteria that are resistant to acidic environments^8^. These pathogenic bacteria developed virulence characters by enhancing their adhesion and invasion tools for host cells, which led to biofilm formation^3, 9^, a prominent character of virulent and resistant bacteria. In avian *E. coli*, several virulent genes, including *fimA, fimH, and fimC*, and siderophore, were involved in biofilm development. There was a positive correlation between biofilm formation and type-1 fimbriae, curli fimbriae, and other motility genes^10, 11^. Many pathogenic *E. coli* from poultry and humans harbor similar virulence genes, including adhesin, *fimH* and siderophore, as well as biofilm forming genes, and exhibit similar characteristics^3, 12, 13^. Similarly, most of the poultry *E. coli*-harboring virulent genes have the ability to cause extra-intestinal human infection^14^. *E. coli* mobile genetic elements (MGEs), including phage-shock proteins, phage tail proteins, and transposases, are responsible for the increase in resistance and virulence^15^.

The virulence regulators and global gene expression profile of such strains of *E. coli* from poultry are not well established. Therefore, the goal of our work was to study the transcriptome of MDR and highly virulent *E. coli* strain (*E. coli* 381) from fecal samples of poultry exhibiting colibacillosis and compare it with human *E. coli* ATCC 25922 in order to determine the genetic features. Moreover, our study determines the antibiotic resistance profile, biofilm formation assay, and virulence in an *in vivo* chicken model as well as the adhesion, invasion, and intracellular survivability of different types of cell lines. Results from this study demonstrated the unique virulence and gene expression profile of MDR *E. coli* of poultry origin.

## Results

### Bacterial isolation and antimicrobial susceptibility test

A total of 45 ciprofloxacin-resistant poultry *E. coli* isolates were selected for this experiment. *E. coli* ATCC 25922 was used as a reference strain. The minimum inhibitory concentrations (MICs) were obtained from the antimicrobial susceptibility testing for all strains, and found that five isolates (112, 130, 351, 357 and 381) were highly resistant (Table 1). Among these five isolates, *E. coli* 381 exhibited the most resistant profile. Accordingly, for further study these five strains (112, 130, 351, 357 and 381) were pursued further.

**Table 1:** MICs (µg/mL) of five MDR *E. coli* isolates.

Antibiotic Classes	Antibiotic Sub Class	Antibiotics	*E. coli* isolates				
			112	130	351	357	381
			MIC µg/ml				
Quinolones	Fluoroquinolone	Ciprofloxacin	16	128	128	64	64
		Enrofloxacin	4	8	2	0.5	16
	Quinolone	Clavulanic acid	16	32	16	32	128
Tetracyclines	Tetracyclines	Tetracycline	32	16	128	16	64
		Doxycycline	8	1	4	128	1
		Chlortetracycline	1	16	4	1	32
Cephems	Cephalosporin	Ceftriaxone	64	8	16	32	16
		Ceftiofur	64	64	16	32	128
		Cefalothin	16	4	16	8	64
		Cefquinome	64	8	8	32	16
		Ceftazidime	32	64	64	64	128
		Cefotaxime	64	32	128	64	128
Phenicols	Carbapenems	Imipenem	32	16	8	4	32
	Phenicols	Chloramphenicol	4	0.125	2	0.5	16
		Florfenicol	4	4	16	64	8
	Polypeptides	Polymyxin B	1	0.5	0.5	0.5	0.5
Penicillins	Aminopenicillin	Amoxicillin	64	64	32	8	64
		Ampicillin	64	64	64	8	32
Aminoglycosides	Aminoglycosides	Amikacin	128	32	1	32	64
		Neomycin	32	16	8	2	128
		Gentamicin	128	4	16	8	64
	Aminocyclitols	Spectinomycin	32	16	16	16	64
Monobactams	Monobactams	Aztreonam	16	8	16	16	16
Miscellaneous	Phosphonic acid derivative	Fosfomycin	8	4	2	4	256
Combination	Penicillins + β-lactamase inhibitor	Amoxicillin-Clavulanic Acid	32	128	8	4	128

### Resistant genes detection

A total of fifteen resistant genes, mostly prevalent in poultry *E. coli* strains, were screened^16–25^. A PCR assay was performed and found that of 16 resistant genes from different antibiotic classes (β-lactams and β-lactams), most of the genes were prevalent in the selected isolates. Resistant genes from chloramphenicol (*cat-A1* and *cml-A*) and tetracycline (*tet-A* and *tet-B*) were present in all selected isolates. Only isolate 381 showed the presence of the quinolone-resistant gene *qnr-S*; none of the isolates contained other quinolone-resistant genes. All of the isolates contained β-lactam resistant genes, mainly *CTX-M, CTX-M-1, TEM-1*, and *OXY*. Isolate 381 contained the maximum number of detected genes (10 out of 16). The prevalence of the investigated genes in selected *E. coli* isolates is presented in Table 2 with their references.

**Table 2:** Selected clinical isolates of poultry *E. coli* producing resistant genes.

Isolate No	Antibiotic resistant genes detected
112	aac(3)-IV, cat-A1, cml-A, tet-A, tet-B, CTX-M, TEM-1, OXY
130	cml-A, tet-A, tet-B, CTX-M, CTX-M-1
351	aac(3)-IV, cat-A1, cml-A, tet-A, tet-B, CTX-M, CTX-M-1, OXY
357	cml-A, tet-A, tet-B, CTX-M, CTX-M-1, OXY
381	aac(3)-IV, cml-A, qnr-S, tet-A, tet-B, CTX-M, CTX-M-1, TEM-1, MdtF, MdtG

### Biofilm formation

**Figure 1:**
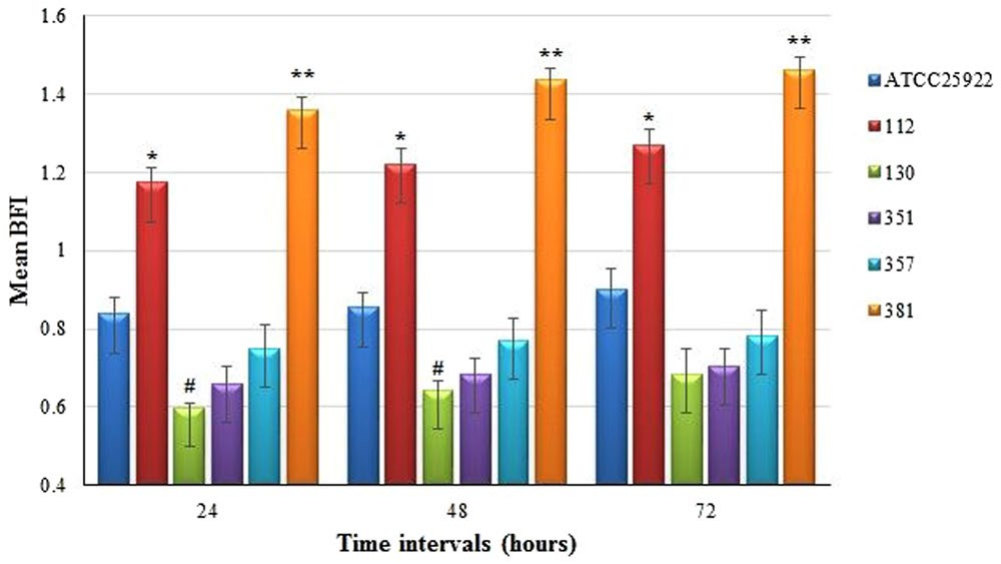
Biofilm formation of five isolates of *E. coli* and reference strain at different time intervals. The results are shown in the form of mean biofilm formation index (BFI) of three independent repeats and compared to *E. coli* ATCC 25922. Statistical significance (P ≤ 0.05) was calculated using two-tailed t-test. *P- values of ≤ 0.05 was considered significant higher than control. ^#^P values of ≤0.05 was considered significant lower than control. **P- values of ≤0.05 was considered significant higher than control and *.

Using the crystal-violet staining method, five MDR *E. coli* isolates were further investigated for their ability to form biofilm *in vitro*. At different time intervals, several levels of biofilm formation were noted for *E. coli* isolates. A positive correlation was found between the time of incubation and biofilm formation. Biofilm formation was observed at 24, 48, and 72 hours post incubation (PI), and the highest level was observed at 72 h PI. Among the tested strains, *E. coli* 381 was the strongest biofilm producer (Fig. 1), and there was a significant difference in the biofilm formation of *E. coli* 381 and the other tested strains.

### Adhesion, invasion, and survivability assay

**Figure 2:**
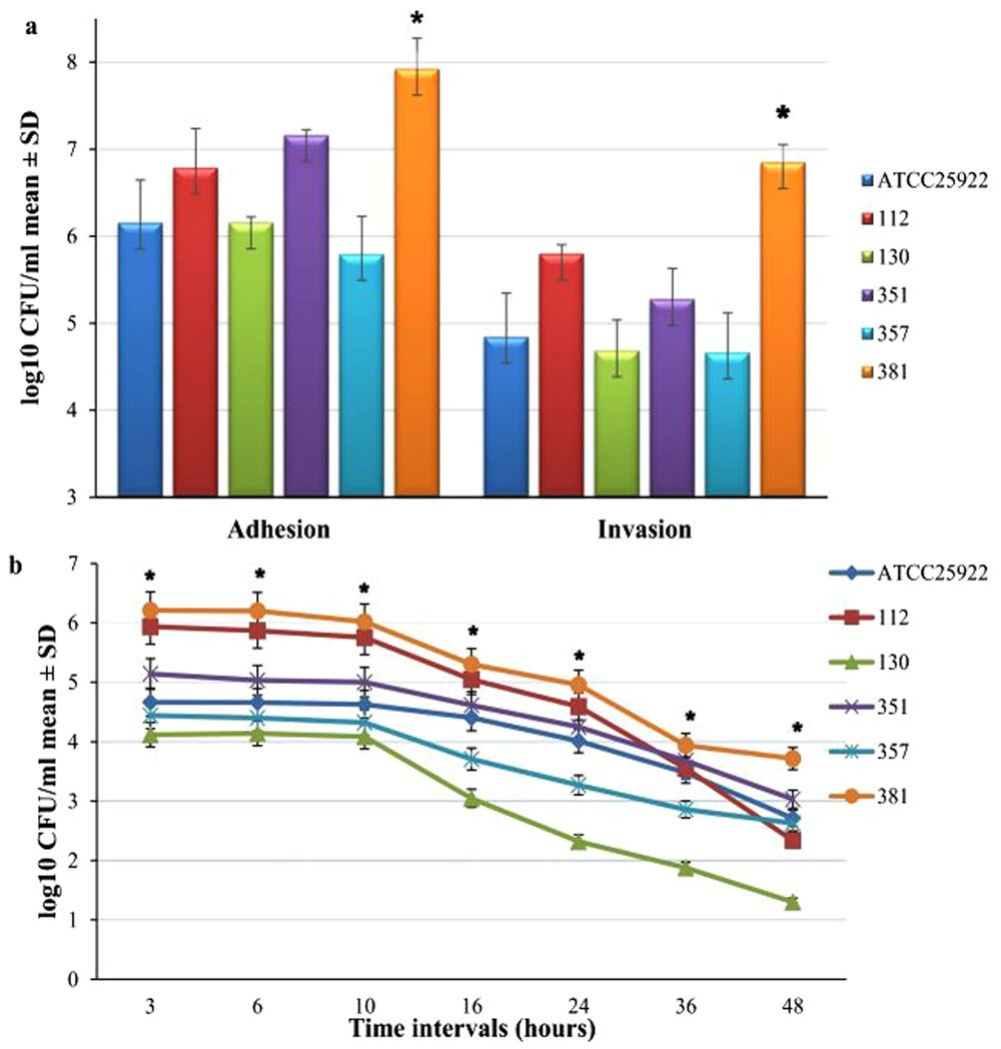
*In vitro* virulence assay of five *E. coli* isolates along with ATCC 25922 in Caco-2 cells. (**a**) Number of adherent and internalized bacteria. The results are presented as log10 of the mean ± standard deviation (SD) CFU/mL of three independent repeats and compared to *E. coli* ATCC 25922. (**b**) Intracellular survival rate of *E. coli* isolates at different time intervals. The results are presented as log10 CFU/mL mean ± SD of survival rate. Asterisk (*) represents statistical significance (P ≤ 0.05) using two-tailed t-test.

**Figure 3:**
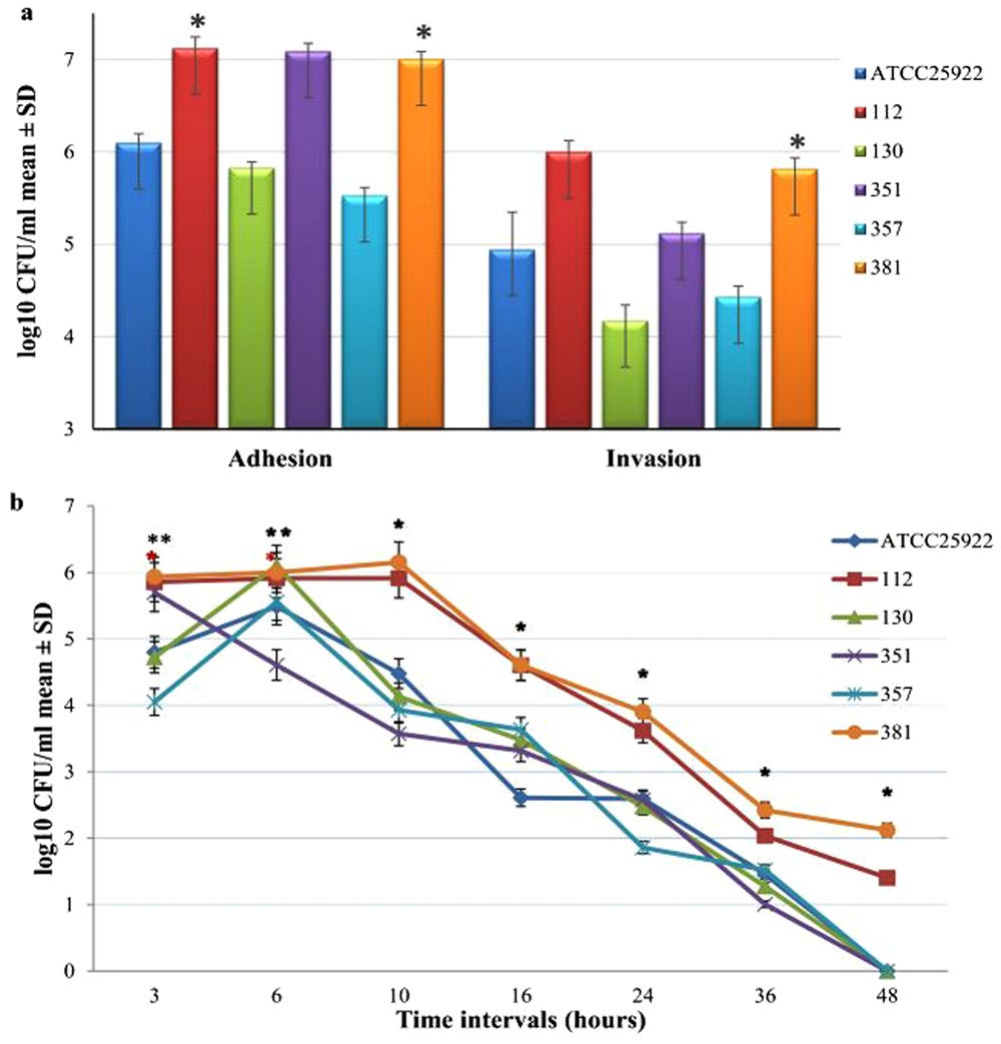
*In vitro* virulence assay of five *E. coli* isolates along with ATCC 25922 in macrophage RAW 264.7 cells. (**a**) Number of adherent and internalized bacteria. The results are presented as log10 of the mean ± standard deviation (SD) CFU/mL of three independent repeats and compared to *E. coli* ATCC 25922. (**b**) Intracellular survival rate of *E. coli* isolates at different time intervals. The results are presented as log10 CFU/mL mean ± SD of survival rate. Asterisk (*) represents statistical significance (P ≤ 0.05) using two-tailed t-test.

The adhesion, invasion, and internalization of *E. coli* are considered to have very strong, virulent, and resistant potentials, so these abilities were tested in selected MDR *E. coli* isolates. For this purpose, two types of cell lines, Caco-2 gut cells and murine macrophage RAW 264.7, were used. In both cell lines, the isolate *E. coli* 381 showed significantly higher trends for adhesion and invasion compared to other isolates and the reference strain *E. coli* ATCC 25922 (Fig. 2a and Fig. 3a).

The experiment was extended further to investigate the internalization of these MDR isolates in the same cell lines at different time intervals (3, 6, 10, 16, 24, 36, and 48 hours post infection). In Caco-2 cells, all tested *E. coli* isolates survived and were not cleared, even at 48 hours post infection. At each time interval, isolate *E. coli* 381 was significantly differentiated from the reference strain and had the highest survival rate among all tested isolates (Fig. 2b) (P ≤ 0.02 at 3 h, P ≤ 0.03 at 6 h, P ≤ 0.022 at 10 h, P ≤ 0.02 at 16 h, P ≤ 0.011 at 24 h, P ≤ 0.006 at 36 h, and P ≤ 0.003 at 48 h).

In the RAW 264.7 cell line, there was little increase in the bacterial numbers during the initial time intervals; after 10 h, the internal survival bacteria decreased. The bacteria cleared at 48 h, except for isolates 112 and 381. However, only isolate 381 was significant. Isolate 381 exhibited significant survival in murine macrophages RAW 264.7 (Fig. 3b). The survival rate of isolate *E. coli* 381 was significantly greater than the reference strain and the other tested isolates (P ≤ 0.003 at 3 h, P ≤ 0.002 at 6 h, P ≤ 0.04 at 10 h, P ≤ 0.02 at 16 h, P ≤ 0.011 at 24 h, P ≤ 0.003 at 36 h, and P ≤ 0.015 at 48 h).

### Determination of the lethal dose 50 (LD_50_)

In order to validate the results of the *in vitro* experiments, an *in vivo* experiment was performed using specific pathogen-free (SPF), two-day old broiler chickens. Using oral and intraperitoneal administration routes, the LD_50_ of three highly virulent and resistant *E. coli* isolates (112, 357 and 381) and one reference strain ATCC 25922 were determined. Different mortality rates were observed in the different groups. The lethality rate was significantly different for isolate 381. With the lowest dose, isolate 381 exhibited the highest mortality rates, for both oral administration and intraperitoneal administration. Isolate *E. coli* 381 was observed with mean LD_50_ of 5.6 × 10^6^ and 2.3 × 10^5^ during oral and intraperitoneal administrations, respectively, almost 100- and 1000-fold higher than the control strain. The mean LD_50_ values for other isolates were not significantly different from the reference strain, as shown in Table 3. Therefore, isolate *E. coli* 381 considered the most virulent strain among all tested isolates.

**Table 3:** Determination of LD50 of the three multidrug resistant *E. coli* isolates in two-day old chickens.

Isolates	Oral administration		Intraperitoneal administration	
	Mean LD_50_	P-values	Mean LD_50_	p-value
ATCC 25922	1.53E+08	0.00	3.51E+08	0.00
112	1.33E+08	0.196	1.25E+08	0.185
357	1.32E+08	0.479	2.04E+08	0.204
381	5.66E+06	0.004	2.32E+05	0.005

### Phylogenetic group

The phylogenetic group of MDR isolate 381 was determined via two-step triplex polymerase reaction^26^. Gene *yjaA* was present in this isolate; gene *chuA* and DNA fragment TspE4C.2 were absent. Therefore, strain 381 belongs to phylogenetic group A.

### Transcriptome analysis

In order to identify the molecular mechanism of virulent regulators in MDR *E. coli*, further transcriptome analysis of the most significant strain, *E. coli* 381, was performed and compared to the transcriptome profile of reference strain *E. coli* ATCC 25922. A total of 30 M reads were obtained with a clean ratio of 81%. Of the 5141 total transcripts identified, 2574 differentially expressed genes (DEGs) were found, with 723 (28.09%) genes up-regulated and 1851 (71.91%) genes down-regulated (Supplementary Tables 1 and 2). The differential expression of the DEGs is also presented in the form of heat-map in Supplementary Data, Fig. S1.

To explore the possible role of DEGs in the resistance and virulence characteristics of isolate 381, the Kyoto Encyclopedia of Genes and Genomes (KEGG) pathway analysis and Gene Ontology (GO) classification were performed. The KEGG analysis found that 1652 DEGs played different roles in 132 important pathways. GO terms were assigned to 1918 (74.51%) DEGs, which were further divided into the following three categories: cellular component, molecular function, and biological process. Out of 1918 DEGs, 90 (4.69%), 896 (46.72%), and 932 (48.59%) genes were identified as the cellular component class, molecular functioning class, and biological process class, respectively.

A large number of significantly up-regulated genes found in *E. coli* 381 played an important role in virulence and resistance (Table 4). These include the genes for encoding adhesion and fimbrial attachment proteins (*adhesin DR76_RS14730, FimA, FimC, FimH, csgC, csgF* and *Fic*), invasion proteins (*inv, NlpD* and *YcgZ*), biofilm forming proteins (*bssR* and *bdm*), and toxicity related proteins (*hha* and *DR76_RS04815*). Many stress-controlling genes for encoding the multidrug ABC transporter (DR76_RS00715), multidrug resistance proteins (*MdtB, MdtF* and *MdtG*), porin proteins (*ompE* and *DR76_RS16805*), and the MATE efflux family protein (*DR76_RS14675*) were among the up-regulated genes. Several stress-controlling genes were also up-regulated (*DR76_RS21990, DR76_RS10275, HdeA, IbpB, BhsA, Hsp31* and *CspA*). The genes responsible for the production of metal binding proteins, such as iron ABC transporter, heme ABC transporter permease, manganese transporter protein MntH, zinc transporter, and iron-sulfur cluster binding protein, were also among the overexpressed genes. Some genes encoding proteins, including phage-shock, transposase, and fimbriae (*DR76_RS18230, DR76_RS12215, DR76_RS14730, csgF* and *FimA*), were also up-regulated. Additionally, there were many differentially expressed genes vital to the carbohydrate metabolism pathways, and a majority were up-regulated as well (Supplementary Fig. S2). Genes involved in the quaternary ammonium drug resistance efflux pump, including *sugE*, were also up-regulated.

**Table 4:** Important up-regulated and dwown-regulated genes in *E. coli* 381.

Gene ID/ORF	Gene symbol	Gene Description	Log 2FC	P-value
**Up-regulated genes**				
Virulence				
DR76_RS18720	*inv*	Invasin	1.98	0.01
DR76_RS09810	*NlpD*	Lipoprotein NlpD	1.33	0.03
DR76_RS19050	*ycgZ*	Two-component-system connector protein YcgZ	1.28	0.01
DR76_RS21875	*BssR*	Biofilm formation regulatory protein BssR	6.52	0.01
DR76_RS17570	*bdm*	Biofilm-dependent modulation protein	1.34	0.01
DR76_RS23570	*Hha*	Hha toxicity attenuator B conjugation-related protein	1.36	0.01
Resistance				
DR76_RS00715		Multidrug ABC transporter ATP-binding protein	1.99	0.01
DR76_RS05060	*MdtF*	Multidrug resistance protein MdtF	4.28	0.01
DR76_RS20295	*MdtG*	Multidrug resistance protein MdtG	2.70	0.01
DR76_RS01425	*MdtB*	Multidrug resistance protein B	1.33	0.01
DR76_RS24475	*OmpE*	Outer membrane phosphoporin protein E	1.73	0.01
DR76_RS16805		Porin	1.18	0.03
DR76_RS03370	*rarD*	Chloramphenicol resistance permease RarD	1.17	0.01
DR76_RS23475	*fsr*	Fosmidomycin resistance protein	1.18	0.01
DR76_RS14675	*mate*	MATE efflux family protein	1.55	0.01
DR76_RS20275	*cybB*	Cytochrome B561	1.51	0.01
DR76_RS24965	*sbmC*	DNA gyrase inhibitor protein	2.15	0.01
DR76_RS11510	*SugE*	Quaternary ammonium compound-resistance protein	1.57	0.01
Stress				
DR76_RS21990	*dps*	DNA protection during starvation protein	3.09	0.01
DR76_RS10275	*cstA*	Carbon starvation induced protein	3.94	0.01
DR76_RS05200	*uspB*	Universal stress (ethanol tolerance) protein B	4.53	0.01
DR76_RS15340	*uspC*	Universal stress protein C	2.08	0.01
DR76_RS02730	*uspD*	Universal stress protein D	1.35	0.01
DR76_RS16580		Stress response membrane	1.54	0.01
DR76_RS10585		Zinc resistance protein	2.94	0.02
DR76_RS05085	*HdeA*	Acid-resistance protein HdeA	5.30	0.01
DR76_RS05090	*HdeB*	Acid-resistance protein HdeB	5.17	0.01
DR76_RS05080	*HdeD*	Acid-resistance protein HdeD	5.81	0.01
DR76_RS20000	*BhsA*	Multiple stress resistance protein BhsA	1.30	0.01
DR76_RS04045	*IbpB*	Heat shock chaperone IbpB	2.80	0.01
DR76_RS04040	*IbpA*	Heat shock protein IbpA	1.91	0.01
DR76_RS14975	*Hsp31*	Heat shock protein Hsp31	2.21	0.01
DR76_RS21250	*HspQ*	Heat shock protein HspQ	2.00	0.01
DR76_RS05740	*Hsp15*	Ribosome-associated heat shock protein Hsp15	1.35	0.01
DR76_RS04820	*CspA*	Cold shock protein CspA	1.50	0.02
DR76_RS16900	*asr*	Acid-shock protein	1.25	0.01
MGEs				
DR76_RS18230		Phage-shock protein	1.72	0.01
DR76_RS18245		Phage-shock protein	1.39	0.01
DR76_RS12215		Transposase	2.46	0.01
DR76_RS05670		Transposase	1.26	0.01
Transporters/Regulators				
DR76_RS05095		Magnesium transporter ATPase	6.25	0.01
DR76_RS22320	*zitB*	Zinc transporter ZitB	2.11	0.01
DR76_RS01555	*mntH*	Manganese/divalent cation transporter	2.61	0.01
DR76_RS04895	*dpp*	Dipeptide/heme ABC transporter permease	2.12	0.01
DR76_RS23075		Iron-enterobactin transporter membrane protein	1.87	0.01
DR76_RS23065		Ferrienterobactin ABC transporter periplasmic binding protein	1.80	0.01
DR76_RS13325		Cobalt transporter	1.54	0.01
DR76_RS12185		Fe3+ dicitrate ABC transporter permease	1.40	0.01
DR76_RS23085		Iron-enterobactin transporter ATP-binding protein	1.42	0.01
DR76_RS12180		Iron ABC transporter	1.24	0.01
DR76_RS12205	*fecR*	Fec operon regulator FecR	2.33	0.01
DR76_RS06040	*bfr*	Bacterioferritin	5.87	0.01
DR76_RS06035		Bacterioferritin-associated ferredoxin	2.61	0.01
DR76_RS12190	*FecC*	Fe(3+) dicitrate transport system permease protein FecC	1.16	0.01
DR76_RS12840	*FhuF*	Ferric iron reductase involved in ferric hydroximate transport	2.65	0.01
DR76_RS11585		Iron-sulfur cluster binding protein	1.38	0.01
DR76_RS16445		Iron-sulfur cluster assembly scaffold protein	4.56	0.01
DR76_RS00310		Catecholate siderophore receptor CirA	1.22	0.01
Fimbrial/Flagellar Protein				
DR76_RS05905	*Fic*	Cell filamentation protein Fic	4.17	0.01
DR76_RS20385	*csgF*	Curli assembly protein CsgF	2.22	0.01
DR76_RS20355	*csgC*	Curli assembly protein CsgC	2.07	0.01
DR76_RS12570	*FimA*	Type-1 fimbrial protein subunit A	3.22	0.01
DR76_RS12580	*FimC*	Molecular chaperone FimC	2.37	0.01
DR76_RS12600	*FimH*	Fimbrial protein FimH	1.97	0.01
DR76_RS12575	*FimI*	Fimbrin fimI	3.13	0.01
DR76_RS14730		Adhesin	3.39	0.01
DR76_RS25465		Tail fiber assembly protein	2.39	0.01
Down-regulated genes				
Virulence				
DR76_RS07065				
DR76_RS07265	*RelE*	Toxin RelE	−6.71	0.01
DR76_RS08005		Capsule polysaccharide transporter	−12.24	0.01
DR76_RS09375	*ImpG*	Type VI secretion protein ImpG	−16.84	0.01
DR76_RS12265	*HlyC*	Hemolysin activation protein hlyC	−7.05	0.01
DR76_RS12560	*FimE*	Type 1 fimbriae regulatory protein FimE	−4.27	0.01
DR76_RS15150	*ompC*	Outer membrane porin protein C	−15.25	0.01
DR76_RS25010	*YoeB*	Toxin YoeB	−1.46	0.01
Resistance				
DR76_RS03915		Multidrug resistance protein MdtL	−1.17	0.02
DR76_RS22090		Multidrug ABC transporter ATP-binding protein	−1.40	0.02
Stress				
DR76_RS15385		Chemotaxis protein CheR	−4.63	0.01
DR76_RS15395		Chemotaxis protein CheY	−4.69	0.01
DR76_RS15400		Chemotaxis protein CheZ	−4.77	0.01
DR76_RS17090	*cspB*	Cold shock-like protein CspB	−13.40	0.01
DR76_RS17115	*cspI*	Cold shock-like protein CspI	−15.19	0.01
DR76_RS18010	*uspF*	Universal stress protein F	−1.96	0.01
DR76_RS22985	*uspG*	Universal stress protein G	−2.37	0.01
MGEs				
DR76_RS14445		Transposase	−8.87	0.01
DR76_RS19120		Phage tail protein	−16.82	0.01
Transporters				
DR76_RS18300		Multidrug transporter	−16.81	0.01
DR76_RS24615		Autotransporter	−12.61	0.01

**Figure 4:**
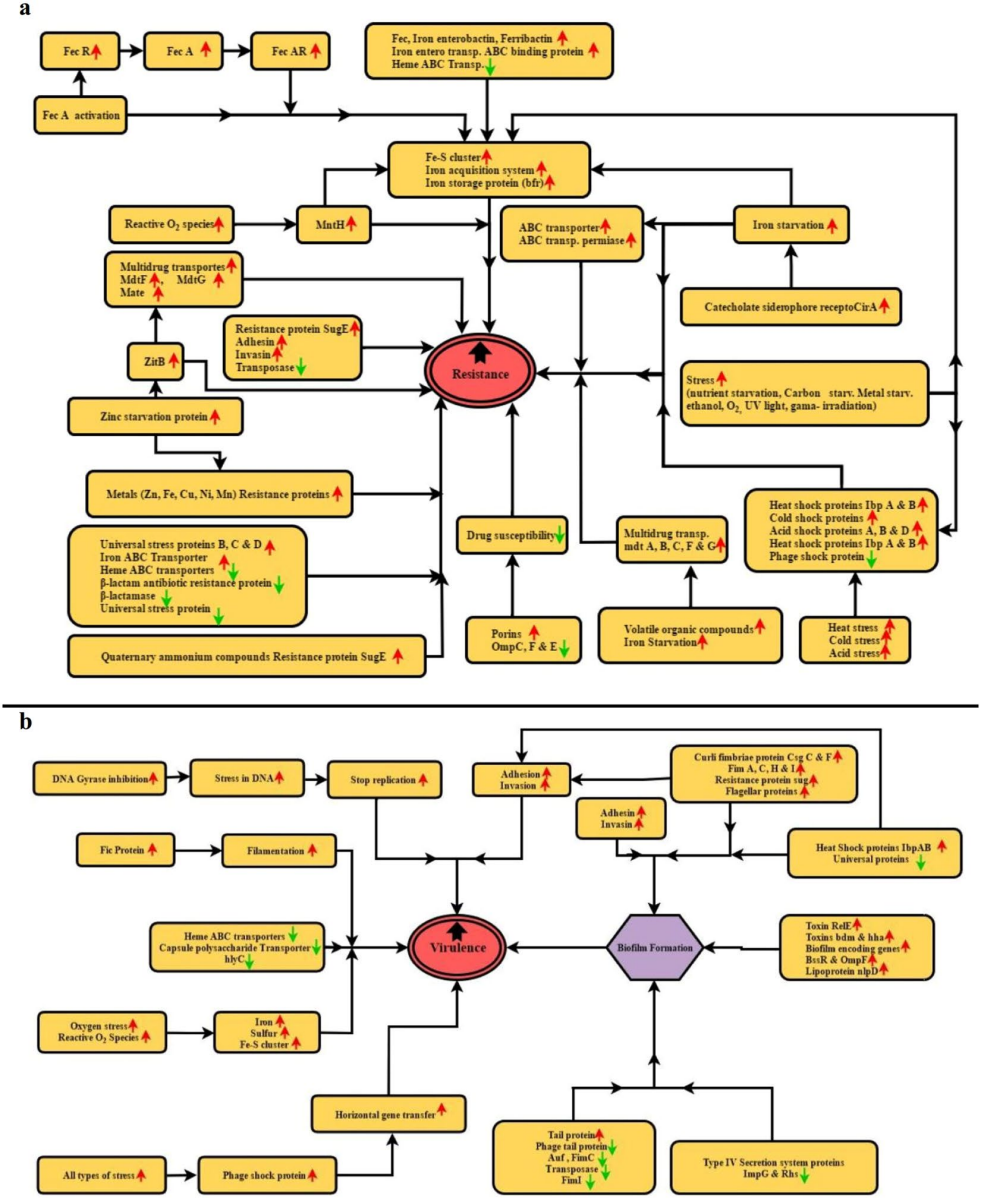
Proposed resistance (**a**) and virulence (**b**) regulating genes and associated factors in isolate *E. coli* 381. Up-regulated genes and factors are shown with red-colored upward arrows while down-regulated genes and factors are shown with green-colored downward arrows.

Similarly, several genes related to our field of interest were down-regulated in *E. coli* 381 compared to reference strain (Table 4). Of these genes, some encode the proteins necessary to combat stress (*uspF* and *CheR*). The virulence-associated toxin and antitoxin genes (*YhaY, RelE, YeeU, YoeB, DR76_RS12335* and *DR76_RS13310*), and outer membrane protein/porin and secretion system-related genes (*DR76_RS08000, DR76_RS08575, DR76_RS18510, DR76_RS19265, Rhs* and *ImpG*) were among the significant down-regulated genes. The Down-regulated genes (*DR76_RS07270, DR76_RS08315, DR76_RS14570, DR76_RS18070* and *DR76_RS18315*), encoding transporters and regulators, also played a crucial role in the resistance and virulence mechanisms. Fimbrial proteins, type IV pilin, phage-related proteins, and molecular chaperons were among the repressed proteins (*AufA, SetB, DR76_RS14920, DR76_RS19150, ParB* and *FimC*). One gene-encoding, multidrug-resistant protein (*MdtL*) and two gene-encoding, multidrug transporters (*DR76_RS22090* and *DR76_RS25275*) were also down-regulated. Schematic diagrams of the proposed resistance- and virulence-regulating genes and associated factors of isolate *E. coli* 381 are given (Fig. 4a and b).

### Validation of RNA-Seq Results via RT-qPCR

RT-qPCR was performed by selecting nine important DEGs to validate the RNA sequencing data. These genes were as follows: *NlpD*, (lipoprotein), *BssR* (biofilm formation regulatory protein), *hha* (toxicity attenuator B conjugation-related protein), *MdtF* (multidrug-resistance protein), *mdtG* (multidrug-resistance protein), *FicC* (molecular chaperon), *DR76_RS08135* (transposase), *ImpG* (type VI secretion protein), *RelE* (toxin), and *CspB* (cold shock-like protein) were expressed during RT-qPCR with fold changes of 1.83, 5.85, 2.87, 5.32, 1.82, 3.03, −13.74, −10.87, and −10.26, respectively. Their expression levels were compared with the test and reference strains. These tested genes were expressed in RT-qPCR similarly to those in the RNA-Seq expression (Supplementary Fig. S3a). Person’s correlation coefficient (r) was 0.963, indicating that the results from both techniques were strongly correlated (Supplementary Fig. S3b).

## Discussion

Two-thirds of the total antibiotics produced per year globally are consumed in livestock sector^4^; therefore, food-producing animals and poultry raised with antibiotics contain important MDR organisms. These MDR organisms can spread to the human population through direct contact or consumption^6^. Among these MDR organisms, *E. coli* is the most threatening due to high economic losses and food contamination rates^27^. These MDR organisms carry antibiotic-resistant genes with the potential to spread to other populations^28^. The abundant use of antibiotics in poultry farms has been associated with treatment failure and the development of antibiotic resistance itself^29^. A study showed that *E. coli* from poultry in China were resistant to at least 18 different antibiotics^16^. In this study, *E. coli* 381 showed resistance to 20 of 26 tested antimicrobial drugs, and exhibited significant MDR behavior.

The following genes were present in the tested isolates and previously identified in *E. coli* from avian samples: aminoglycoside acetyltransferase *gene aac(3´)-IV*, which not only acetylates aminoglycosides but also fluoroquinolones such as norfloxacin and ciprofloxacin, chloramphenicol-resistant genes *cml-A* and *cat-AI*, and tetracycline-resistant genes *tet-A* and *tet-B* ^17, 30^. Quinolone-resistant genes provide a low level of resistance; however, their presence may facilitate the development of resistance against other antibiotic agents and cause treatment failure^31^. In a previous study, *qnr-S* was the most common quinolone-resistant gene found in poultry *E. coli* ^30^.

The production of β-lactamases leads to resistance of third and fourth generation cephalosporins. The random use of β-lactams can result in the co-resistance of β-lactams and non- β-lactams in *E. coli*, since plasmid-mediated β-lactamases are the main reservoir of MDR genes^32^. Most of the identified β-lactamase genes were present in these pathogenic MDR *E. coli* isolates of poultry origin. A previous study found that the fecal *E. coli* of healthy broilers also harbor β-lactamase genes^33^. MDR proteins MdtF and MdtG, involved in the resistance nodulation division (RND) type efflux pump, were detected by PCR only in *E. coli* isolate 381. The over expression of MdtF and MdtG decrease the susceptibility to a number of antibiotics through the efflux pump, including erythromycin and fluoroquinolone^18, 19^. The presence of these resistant genes support the previous results of antibiotic susceptibility assay and indicate the threat of their spread to other populations, especially humans, through contamination.

Most MDR *E. coli* strains have the ability to form biofilm, which not only acts as a barrier against the treatment of infections but also increases the virulence characteristics of these strains^10^. Biofilm is the well-organized accumulation of bacterial structures enclosed by a self-formulated matrix sheath and attached to the host cell^34^. The biofilm assay found that biofilm formation was time dependent in the tested isolates. Isolate 381 had the highest capability of biofilm formation at each time point compared to the reference strain *E. coli* ATCC 25922.

*E. coli* can survive inside the Caco-2 and RAW 264.7 cell lines for an extended period of time to protect themselves from elimination, antibiotic attack, and host defense mechanism, where *E. coli* acts as a reservoir for spreading of infections^35, 36^. We found that *E. coli* can attack, invade, and survive inside the cells for more than 48 hours, and many studies concur with our results^36, 37^. These findings suggest that *E. coli* 381 is an MDR, invasive, and biofilm-producing strain. For the validation of the *in vitro* results, an *in vivo* study in chickens was conducted. For this purpose, three isolates (112, 357, and 381) were selected. The results clarified that the LD_50_ dose for isolate 381 was the lowest among the tested isolates, depicting additional evidence of its virulence potential. Triplex PCR found that MDR isolate *E. coli* 381 belongs to phylogenetic group A, which is prevalent in intra-intestinal poultry *E. coli* and other domestic animals^38–40^. Poultry *E. coli* isolates belonging to phylogenic group A and share a genetic link with the human *E. coli* causing urinary tract infection and newborn meningitis, which proposes the zoonotic importance of this phylogenetic group^39, 40^. Consequently, the results of the *in vitro* and the *in vivo* studies cleared that *E. coli* 381 was the most virulent and resistant isolate of all tested strains. Therefore, this strain was selected for further study of its gene expression analysis and virulence determinants.

Transcriptional analysis allows scientists to suggest how a composite regulatory network expresses several virulence determinants in organisms^41^. Transcriptome analysis of microbes has become a major, reliable tool to examine genetic potential of the microorganisms via differential gene expression analysis; accordingly, the analysis was of great interest to us^41^. Gram-negative bacteria change the permeability of their membrane to control the intracellular passage of antibiotics through efflux pumps. Of the five major families of multidrug transporters in bacteria, genes related to RND, ATP binding cassette (ABC), and multidrug and toxin extrusion (MATE) transporter families were up-regulated in MDR *E. coli* 381 strain compared to the reference strain. The multidrug resistance proteins mdtABCFG up-regulated in *E. coli* 381 were mainly involved in transmembrane transport. MdtF, or Yhiv, is a multidrug resistance efflux pump of the RND protein family^42^, and its overexpression leads to iron starvation and reduced susceptibility to many drugs (e.g., erythromycin and ethidium bromide^18, 43^). Similar to other MDR-type drug exporters, MftF and MdtE also confer β-lactam antibiotic resistance in *E. coli* ^44^. The overexpression of the *mdtG* gene confers resistance to fosfomycin and deoxycholate^45^; *mdtABC* genes lead to resistance against novobiocin^46^. These results were supported by another transcriptome study of fluoroquinolone resistant *E. coli*, where *mdtG* was up-regulated almost 2-fold^47^. The overexpression of MATE family transporters confer resistance to quinolone, fluoroquinolones, trimethoprim, chloramphenicol, and fosfomycin in *E. coli* ^48, 49^. Thus, the up-regulation of multidrug transporters and ABC-binding proteins lead to the reduced antibiotic concentration inside the cells, a bacterial defense mechanism. In consequences, these MDR-genes confer resistance to the bacteria, which often results in treatment failure.

In this study, the up-regulation of gene “DR76_RS24965,” also known as the “*sbmC*” encoding DNA gyrase-inhibitor protein, was observed in *E. Coli* 381. It provides protection from toxins that attack DNA gyrase by blocking the toxin mechanism, reducing the development of lethal double-strand breaks, and protecting from synthetic quinolones and alkylating agents that attack independently on DNA gyrase^50, 51^. These processes suggest an overall function in defending the DNA impairment. On the other hand, a fosmidomycin-resistant gene (*fsr*) has been identified in glpT mutant strains, where the resistance was due to the efficient bacterial efflux of the drug. Up-regulation of the *fsr* gene was also identified in isolate 381 and reported earlier for *E. coli* K-12^52, 53^. In addition, the quaternary ammonium compound-resistant protein *SugE*, a member of the small multidrug resistance (SMR) protein family, was up-regulated in MDR *E. coli* 381. In many microbes, this gene is responsible for the efflux and resistance to many ammonium compounds, including cetyldimethylethyl, acriflavin tellurite, and cetylpyridinium^54, 55^.

Interestingly, there were a number of differently expressed genes in MDR *E. coli* 381 encoding the porins and most of them were down-regulated. Bacteria can alter membrane permeability and control the passage of drugs via the down-regulation or loss of porins; therefore, porins act as the first line of defense against antibiotics^56^. The loss or decreased expression of the porins *ompC, ompF*, and *opmE* lowered the drug susceptibility of bacteria and conferred resistance to many antibiotics (e.g. fluoroquinolone^57, 58^), and metal ions (Cu^2+^ and Ag^+^^57–59^). The down-regulation of capsular polysaccharide-related genes promote bacterial survival during infection via changes in the recognition of capsule-targeting antibiotics, which results in high resistance and chronic infection^60^. The repression of autotransporters, the specific targets of several antibiotics, was observed in this study, which can lead to prolonged treatment or treatment failure^61^. The differential expression analysis indicates that there are multiple cellular pathways implicated in the development of resistance in *E. coli* 381. A thorough understanding of these pathways is needed to identify novel targets and antimicrobial therapeutics.

The transcriptional analysis of the virulence genes in *E. coli* were evaluated. Adhesin and fimH are fimbrial tip adhesion molecules in *E. coli* which facilitate adhesion, internalization, and biofilm formation^62, 63^. In the present study, many fimbrial genes that encode the type-1 fimbrial proteins *fimA, fimC*, *fimH*, and *fimI* were involved in infection^11, 62^. *FimI* activate fimbrial biogenesis^64^ was up-regulated, suggesting the virulence status of the *E. coli* 381 strain.. The pathogenic and virulence-associated gene *fimC* was the most abundant gene isolated from the MDR *E. coli* of poultry in China^11^. The co-presence and overexpression of type-1 fimbriae (*fim*) and curli fimbriae (*sug*) were significant findings in the *E. coli* 381 strain. This combination has already been reported in human *E. coli* and avian *E. coli* strains, where they conferred virulence and biofilm formation^12^. Other up-regulated curli fimbriae genes in these data were *csgC* and *csgF*, also associated with virulence and biofilm formation^65^. Furthermore, the overexpression of the adhesion gene *ompF*, invasin genes *inv* and *ychO* ^9^, and biofilm formation regulatory genes *ycgZ*, *bssR*, and *bdm* ^66^ also contributed to the virulence of the strain. On the other hand, some repressed curli proteins and type IV pilin were possibly involved in the programmed dispersion of biofilm and spread of infection^67^.

In addition, many toxin genes were up-regulated in the MDR *E. coli* 381 strain. The overexpression of *bdm, hha*, and gene-encoding small toxic polypeptide cause high rate of cell death by the activation of the stress-response^68^. Also, *hha* causes membrane aggregation, biofilm formation, conjugation internalization, and flagella gene activation^69, 70^. In addition, lipoprotein nlpD is an outer membrane protein that supports cell survival and confers virulence in *E. coli*, was up-regulated^71^. The connector protein YcgZ, which has a two-component-system, responds to acid chemicals, and confers resistance to toxins, therefore making the strain more virulent, was also up-regulated^72^. Augmented by biofilm-forming and colonization factors, the overexpression of these important genes made MDR *E. coli* 381 a highly virulent strain.

The transcriptional analysis of *E. coli* 381 provided an evaluation of the stress genes and metal homeostasis. Heat shock proteins form a network of multifactorial stress reactions, where they control basic metabolic enzyme functions. These proteins are further controlled by heat shock factors, which are mainly altered under stress conditions^34^. In *E. coli* 381, the enhanced expression of acid shock protein (asr)^73^, acid resistance proteins hdeA, hdeB, and hdeD^12^, heat shock proteins IbpA, IbpB, Hsp31, and HspQ^15, 74^, and cold shock protein cspA^75, 76^ reflected the genetic potential for survival of the pathogen in a diversified and harsh environment. Most enteric bacteria like *E. coli* contain acid-shock resistant proteins. These bacteria have the ability to thrive in and pass through the mammal or poultry host gastrointestinal tract (GIT), where they cause infections and carry resistance to many antibiotics^77^. The up-regulation of carbon starvation induced protein (cstA) helped the bacteria to cope with carbon and energy limitation^78^. There are six universal stress proteins^47^, three (*uspB, uspC* and *uspD*) of which were overexpressed in MDR *E. coli* 381 compared to the reference strain. During stress conditions, the overexpression of universal-stress proteins (usp), cold-shock protein (cspE), and phage-shock protein (pspE) were observed in *E. coli* K-12^79^.

Several genes that transport metals, including iron, manganese, potassium, cobalt, nickel, and zinc, were up-regulated in MDR *E. coli* 381. Previous studies from different organisms have proven that these genes are responsible for increased resistance and virulence^80, 81^. The up-regulation of zinc-resistant protein, zinc-transporter, and multidrug-resistant proteins were observed, which are crucial under antibiotic and zinc stress conditions^80^. In addition to several iron-related genes, such as *bfr*, *FecR* and *FecC*, the enhanced expression of iron-sulphur cluster genes were also observed, which can facilitate *E. coli* to react towards variable O_2_ concentrations and result in increased bacteria resistance^82^.

The up-regulation of genes linked to carbohydrate metabolism pathways (Fig. S2) are associated with an increase in fermentation products, and thus confer stress tolerance to bacteria^83, 84^. Most of the overexpressed genes elicit acetate production, which is a good source of carbon; under stress and carbon starvation, acetate production facilitates bacteria resist to these conditions^85^. Toxin-antitoxin (TA) modules are very important for the regulation of bacterial functions, including stress tolerance, antibiotic resistance, and biofilm formation. The TA operons were down-regulated by several stress conditions and nutrient starvation, which released free toxins^86^. Few iron or heme ABC transporters and universal stress proteins were down-regulated in *E. coli* 381 isolate compared to the human reference strain *E. coli* ATCC 25922. This was because many genes with iron and copper acquisition were up-regulated, which might have increased the concentration of copper in the *E. coli* 381 cytoplasm^47, 87^. The repression of some genes encoding phage shock proteins may be due to different environmental stress conditions in *E. coli*, which fortified the resistant character of strain^88^.

Finally, there were many differently expressed mobile genetic elements (MGEs) in *E. coli* 381 that represented the resistance and virulence potential of the strain^79^. For example, up-regulated phage-shock proteins are induced by stress conditions to transport metal ions^89^, preserve natural membrane integrity in extracytoplasmic stress^89, 90^, bolster intramacrophage survival^91^, tolerate heat and ethanol stress^92^, bear acid-base stress^93^, and resist membrane-targeting antibiotics^93^. In addition, transposase-encoding DEGs increased the resistance and virulence of MDR *E. coli* 381 because *E. coli* transposases are involved in reshaping the genome by cutting and pasting the genes, resulting in mutations, disrupting the expression of genes, and participating in horizontal gene transfer^94, 95^.

## Conclusion

The results of this study provide critical information about a highly virulent and resistant *E. coli* strain of poultry origin. Our *in vitro* and *in vivo* studies confirmed the unique and highly virulent determinants of *E. coli* 381. *E. coli 381* was found to be highly virulent in cell culture and 1000-fold more virulent in a chicken model than other strains. Our differential gene expression analysis indicate that there are multiple pathways related to stress, biofilm formation, metal acquisition, transportation, adhesion, and invasion that ultimately led to the resistance and virulence of this strain. A thorough understanding of these pathways is necessary in order to identify novel targets and potential therapeutics.

## Materials and Methods

### Bacterial strains

Clinical pathogenic *E. coli* isolates (n = 929) were previously collected from cases of broilers colibacillosis and initially screened for resistant to ciprofloxacin^29^. Of the 929 isolates, 45 strains with high MICs ( ≥ 16 µg/mL) against ciprofloxacin were selected for this study, due to the fact that chicken is the source of ciprofloxacin-resistant human *E. coli* ^96–98^. *E. coli* ATCC 25922 was used as reference strain in all experiments. Routinely, strains were cultured on Mueller-Hinton agar under incubation condition of 37 °C for 18–24 h.

### Species confirmation and antimicrobial susceptibility test

All strains were cultured on selective MacConkey agar. Species confirmation and the amplification of the gene malB/eco was completed by PCR as described previously^99, 100^. The MICs of 26 antibiotics were determined using the broth microdilution method following the Clinical and Laboratory Standard Institute (CLSI) procedures^101^, with the exceptions of florfenicol and ceftiofur^102^, using *E. coli* ATCC 25922 as the control strain. Based on the MIC results, five multidrug-resistant strains (112, 130, 351, 357 and 381) were selected for further investigations.

### Resistant gene identification by PCR

For resistant gene identification, polymerase chain reaction (PCR) assay was performed using different starting primers as described previously (Supplementary Table 3). MDR *E. coli* strains, along with negative control strain ATCC 25922 *E. coli*, were harvested in MH broth at 37 °C overnight. DNA was extracted systematically using a DNA kit (per the instructions of TianGen Biotech. Co. Ltd. China). The extracted DNA was used as the template for PCR amplification. DNA application was performed as follows: initial denaturation at 95 °C for 4 min, 35 cycles of second denaturation at 94 °C for 30 sec, annealing at 53–60 °C for 60 sec, extension at 72 °C for 60 sec, and final elongation at 72 °C for 10 min^17^. PCR amplicons were visualized via electrophoresis in agarose gel (1%) with an ethidium bromide stain (1 mg/mL) and imaged on a UV transilluminator^17^.

### *In vitro* cell lines and growth conditions

To determine the virulence of the five MDR strains and the reference strain, we used murine macrophage RAW 264.7 cells and human colonic carcinoma Caco-2 cells (China Infrastructure of Cell Line Resource, Wuhan, China). Both cell types were grown as previously described^35^.

### Adhesion, invasion, and intracellular survival assays

Adhesion, invasion, and intracellular survival assays for both cell types were performed as previously described^35, 83^.

### Biofilm assay

The biofilm formation of MDR *E. coli* strains and the reference strain was performed via crystal-violet staining method. For each strain, three independent experiments of three iterations were performed. Biofilm formation index (BFI) and numerical classifications of the biofilm development were calculated as described earlier^103^.

### Lethal dose determination

Our three most resistant and virulent *E. coli* isolates (112, 357 and 381), along with the reference strain ATCC 25922, were selected from our previous experimentation for the determination of their mean lethal dose (LD_50_). Specific-pathogen free (SPF), two-day-old broiler chickens were used for the determination of LD_50_ as described previously^104^. For the oral and intraperitoneal administration of the bacteria, the birds were randomly divided into eight groups (n = 35), four groups for each administration route. Seven chickens from each group were administered 0, 10^5^, 10^6^, 10^7^ or 10^8^ CFU of *E. coli* isolate either orally or intraperitoneally. The survival ratios of each group were observed in two independent experiments for up to three days, and the LD_50_ was calculated as described earlier^83^.

### Phylogenetic group determination

To determine the phylogenetic group of MDR *E. coli* 381 isolate, we performed rapid and simple triplex PCR test. For this PCR, three primer pairs (ChuA.1 and ChuA.2, YjaA.1 and YjaA.2, and TspE4C2.1 and TspE4C2.2) were used. Primer design and all first and second step PCR conditions were followed as previously described^26^.

### RNA-Seq transcriptome analysis

According to the antibiotic susceptibility assay, virulence, and resistance tests, the most virulent and resistant isolate, *E. coli* 381, and reference strain ATCC 25922 were selected for additional comparative transcriptomic studies. The RNA-Seq analysis of bacterial samples were completed as previously described^83^. The samples were harvested at log phase, two samples from the isolate *E. coli* 381 and two samples from the reference strain ATCC 25922. According to the manufacturer’s guidelines, total RNA was extracted from the bacterial isolates using TRIzol (Invitrogen Inc., California, USA) followed by strand-specific RNA-Seq protocol on Illumina HiSeq. 2500 platform (paired-end sequencing; 100 b.p. fragments) at Shanghai Biochip Corporation. Reads longer than 25 nt and ≤ 2 N (ambiguous nucleotides) were preserved. In addition, the paired reads that matched to the silva database were removed (http://www.arb-silva.de/download/arb-files/).

Using the blind and fit-only parameters in the edgeR package, the gene expression of all samples were changed to count per gene (CPG)^105^. From the respective repeats, the mean CPG of gene expressions were intended for the MDR *E. coli* 381 strain and the reference strain, and then their differentially expressed genes were compared. The transcripts were considered as differentially expressed with *P*-values of ≤ 0.05 and fold changes of ≥2. The data were deposited in the Gene Expression Omnibus (GEO) and are available with accession number GSE94978 (http://www.ncbi.nlm.nih.gov/Traces/sra/sra.cgi). For the functional classification of the genes, Gene Ontology (GO) is the best international standardized system. GO provides up-to-date terminology and a comprehensive guide to the genetic properties and products of any organism^106^. Using the structural terminologies (ontology) of biological processes, cellular components, and molecular functions, all differentially expressed genes (DEGs) were further analyzed. The Kyoto Encyclopedia of Genes and Genomes (KEGG) database (http://www.genome.jp/kegg) was consulted to find out the role of DEGs in several pathways^107^.

### Validation of RNA-Seq analysis by RT-qPCR

RNA sequencing results were verified by RT-qPCR. For this purpose, nine important DEGs were selected from isolate *E. coli* 381: *NlpD*, *BssR*, *hha*, *MdtF*, *mdtG*, *FicC*, *DR76_RS08135, ImpG*, *RelE*, and *CspB*. DNA polymerase III subunit alpha (*dnaE*) was used as a reference gene, and RT-qPCR was performed as described previously^108^. The sequence of primers used in RT-qPCR is included in Supplementary Table 4.

### Statistical analysis

Statistical analysis were performed using SPSS version 22.0 (IBM Corp., Armonk, NY, USA). Probit analysis was completed to calculate the values of LD_50_. A two-tailed *t*-test was applied to estimate the mean ± standard deviation and significance level among different strains for biofilm formation, adhesion, invasion, intracellular survivability assays, and for LD_50_. For the comparison of RNA-Seq and RT-qPCR results, a correlation coefficient (r) was determined via Pearson’s analysis. *P*-values of ≤ 0.05 were considered significant in all experiments.

### Ethic statement

All experiments and animal care were endorsed and performed as per the rules and directions of the Animal Care Center, Hubei Science and Technology Agency in China and use of birds was also in accordance with the guidelines and regulations of the agency (SYXK-0044).

## Electronic supplementary material

supplementary figures and Supplementary tables
