# Supplementary material for: Virulence and transcriptome profile of multidrug-resistant Escherichia coli from chicken
Source: Sci Rep. 2017 Aug 21;7:8335. doi: 10.1038/s41598-017-07798-1 (PMC5567091; doi:10.1038/s41598-017-07798-1)
Supplement: Supplementary file 1 — supplementary figures and Supplementary tables [file 41598_2017_7798_MOESM1_ESM.pdf]

Virulence and Transcriptome Profile of Multidrug-Resistant *Escherichia coli* from Chicken

Hafiz I. Hussain, Zahid Iqbal, Mohamed N. Seleem, Deyu Huang, Adeel Sattar, Haihong Hao, Zonghui Yuan

Supplementary Figures and Tables

Supplementary Figures

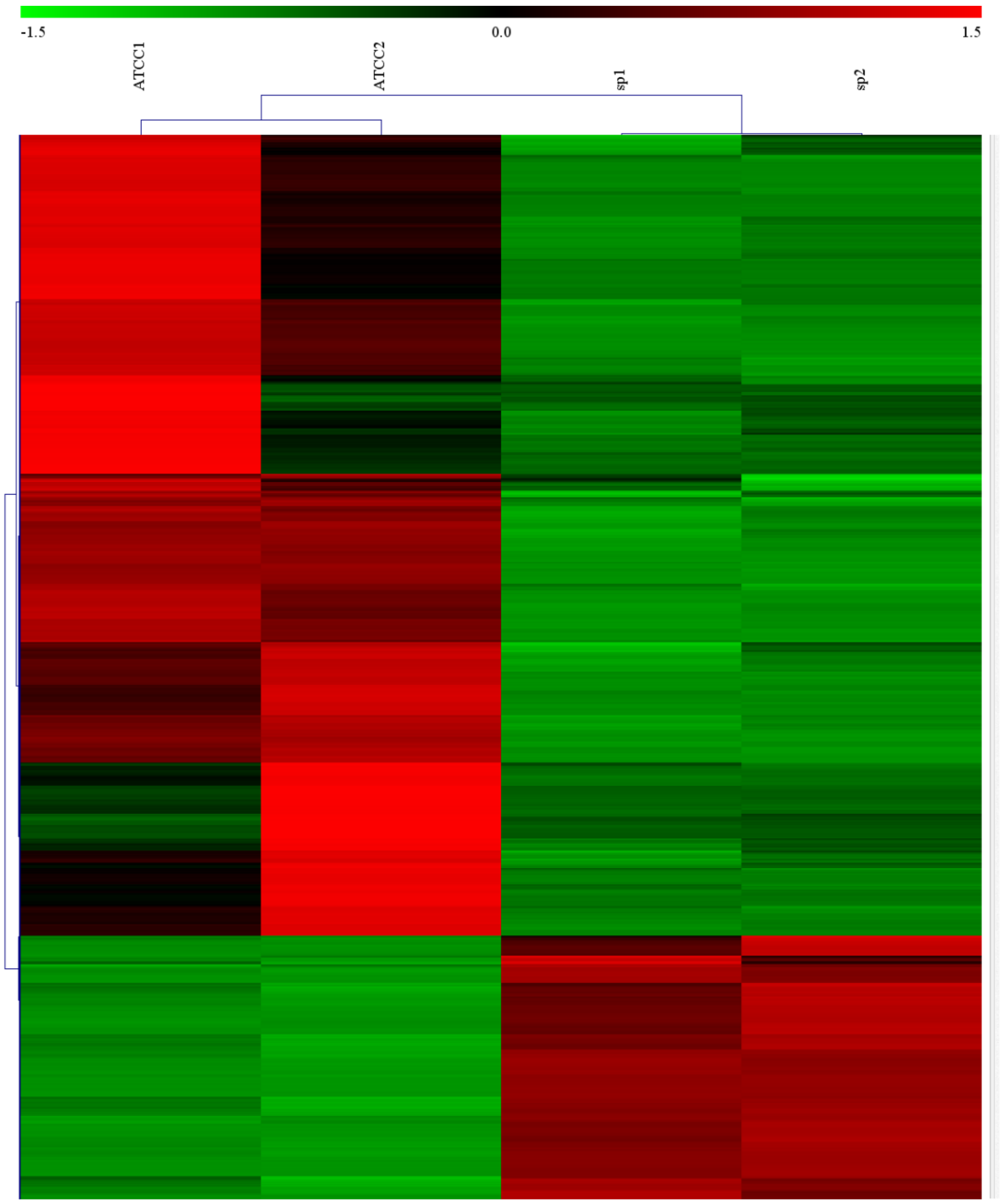

Supplementary Figure S1: Heat map of DEGs of *E. coli* 381 and ATCC 25922. Differential expressed genes were filtered with the absolute value of log2Ratio ≥ 1, based on the false discovery rate < 0.001 between *E. coli* 381 (sp1 and sp2) and ATCC 25922 (ATCC1 and ATCC2). Each column stands for a bacterial strain and each row represents a gene. Red stands for high expression while green stands for low expression.

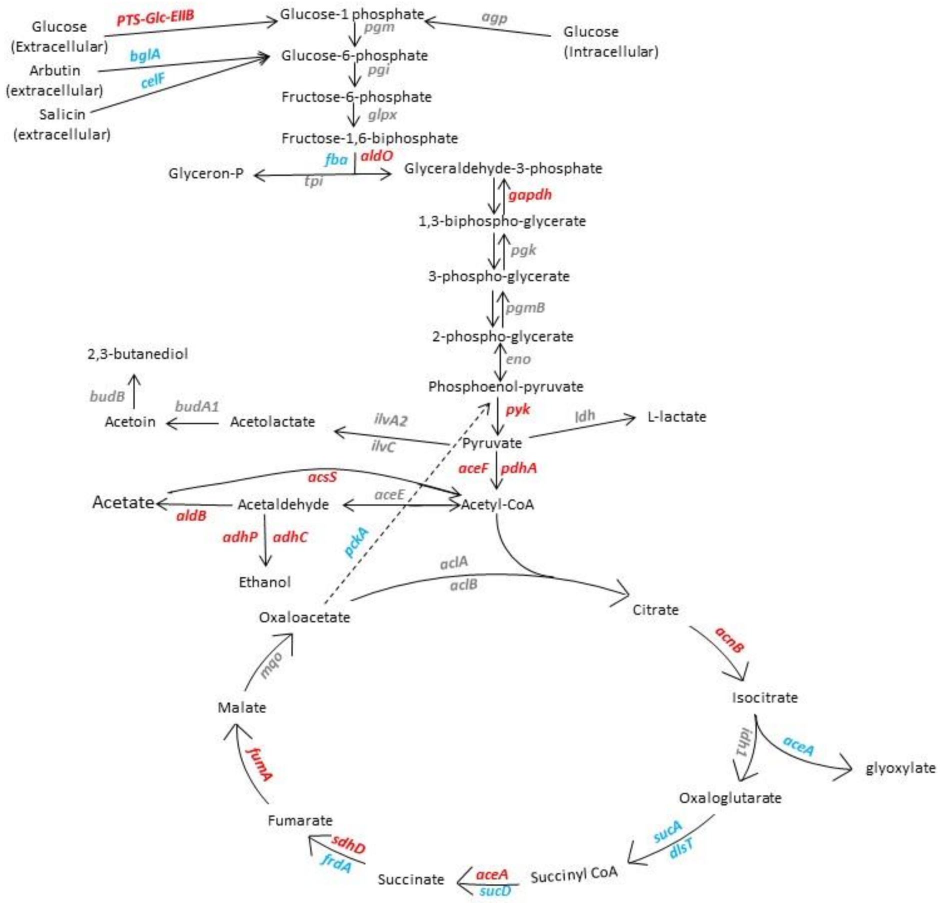

Supplementary Figure S2: Genes related to carbohydrate metabolism pathway in MDR *E. coli* 381 as determined in transcriptome analysis. Up-regulated genes are shown in red color, down-regulated genes in light blue color while genes with no change are given the color dark-gray.

a

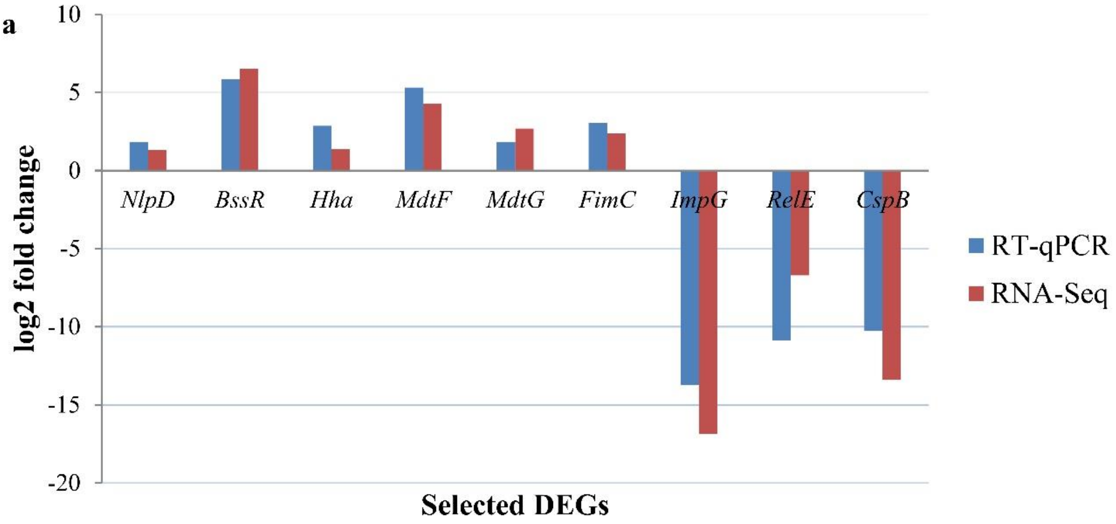

b

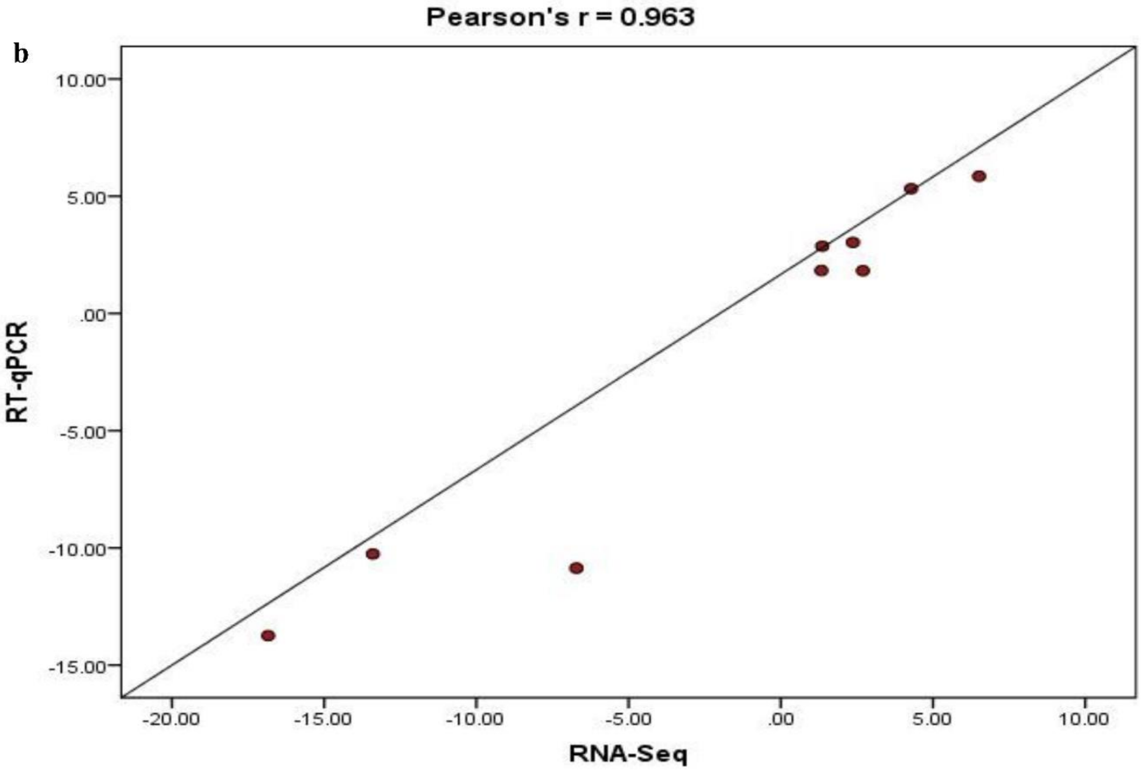

Supplementary Figure S3: Graphical presentation of statistical results of TR-qPCR comparison with RNA-Seq. (a) Differential expression ratio (log2) attained by RT-qPCR and RNA-Seq for the selected DEGs. The comparison pairs has almost similar trends. **(b)** Correlation of differential expression ratio (log2) attained by RT-qPCR and RNA-Seq for the selected DEGs. The Pearson's coefficient (r) is 0.963.

Supplementary Tables

Supplementary Table 1: Up-regulated genes resulted from differential gene analysis of *E.coli* 381 and ATCC 25922

Gene ID	Gene description	log2 FC	P-value
DR76_RS00080	hypothetical protein	2.68	1.1E-09
DR76_RS00155	MerR family transcriptional regulator	4.80	5.8E-25
DR76_RS00160	putative membrane protein	2.97	7.5E-07
DR76_RS00165	osmoprotectant uptake system permease	2.97	3.5E-11
DR76_RS00170	ATP-binding protein	4.12	6.1E-18
DR76_RS00175	osmoprotectant uptake system permease	4.27	1.3E-20
DR76_RS00180	osmoprotectant uptake system substrate-binding protein	2.53	1.7E-09
DR76_RS00200	Yip1 family inner membrane protein	5.77	2.1E-31
DR76_RS00210	hypothetical protein	2.14	2.6E-07
DR76_RS00230	UPF0299 family inner membrane protein	1.08	1.1E-02
DR76_RS00235	membrane protein	1.57	3.5E-04
DR76_RS00285	hypothetical protein	1.64	1.2E-02
DR76_RS00310	catecholate siderophore receptor CirA	1.22	5.0E-03
DR76_RS00315	membrane protein	2.08	4.9E-03
DR76_RS00430	microcin C ABC transporter permease YejB	1.29	2.9E-03
DR76_RS00435	microcin C ABC transporter permease	1.37	7.8E-04
DR76_RS00610	membrane protein	1.48	2.4E-02
DR76_RS00700	hypothetical protein	2.20	1.8E-03
DR76_RS00715	multidrug ABC transporter ATP-binding protein	1.99	1.8E-05
DR76_RS00720	alpha-ketoglutarate-dependent dioxygenase	3.40	2.7E-11
DR76_RS00725	transcriptional regulator	2.47	1.2E-08
DR76_RS00730	thiamine biosynthesis lipoprotein ApbE	1.48	4.7E-04
DR76_RS00800	hypothetical protein	1.37	1.7E-03
DR76_RS00850	hypothetical protein	2.63	5.5E-10
DR76_RS00875	sn-glycerol-3-phosphate dehydrogenase subunit C	1.37	6.8E-03
DR76_RS00990	isochorismate synthase	2.27	2.7E-06
DR76_RS00995	protein ElaB	4.84	3.2E-15
DR76_RS01160	GSH-dependent disulfide bond oxidoreductase	4.67	6.8E-23
DR76_RS01195	hypothetical protein	2.76	2.4E-09
DR76_RS01375	membrane protein	3.88	3.4E-18
DR76_RS01425	multidrug resistance protein B	1.33	6.3E-03
DR76_RS01430	multidrug transporter	1.66	1.0E-03
DR76_RS01445	CoA-transferase	2.82	4.9E-09
DR76_RS01450	transporter	2.20	1.3E-04
DR76_RS01455	oxalyl-CoA decarboxylase	1.17	1.4E-02
DR76_RS01470	hypothetical protein	1.57	1.7E-02
DR76_RS01480	hypothetical protein	1.52	3.2E-04
DR76_RS01540	ion channel protein	1.56	1.3E-04
DR76_RS01545	hypothetical protein	1.33	2.5E-02

DR76_RS01550	hypothetical protein	2.45	4.1E-05
DR76_RS01555	manganese/divalent cation transporter	2.61	1.2E-06
DR76_RS01625	nucleoside permease	1.06	2.6E-02
DR76_RS01650	hypothetical protein	1.12	1.9E-02
DR76_RS01860	transaldolase A	5.02	2.7E-17
DR76_RS01865	transketolase	4.75	1.1E-15
DR76_RS01870	hypothetical protein	1.55	7.3E-03
DR76_RS01915	hydrolase	1.05	1.4E-02
DR76_RS01920	methionine tRNA cytidine acetyltransferase	1.38	1.6E-03
DR76_RS01925	hypothetical protein	1.76	1.5E-04
DR76_RS01930	phosphoribosylaminoimidazole-succinocarboxamide synthetase	1.67	1.3E-02
DR76_RS01935	hypothetical protein	2.08	4.1E-06
DR76_RS01995	phosphoribosylglycinamide formyltransferase	1.51	3.1E-03
DR76_RS02020	membrane protein	1.70	5.7E-04
DR76_RS02150	HscA co-chaperone-2 J domain-containing protein Hsc56-3B IscU-specific chaperone HscAB	1.25	4.3E-03
DR76_RS02165	cysteine desulfurase (tRNA sulfurtransferase)2 PLP-dependent	1.31	2.6E-02
DR76_RS02170	transcriptional regulator	1.48	8.7E-03
DR76_RS02190	stationary phase inducible protein CsiE	1.91	2.0E-05
DR76_RS02200	membrane protein	3.45	2.5E-14
DR76_RS02235	hypothetical protein	1.44	1.3E-03
DR76_RS02320	gap repair protein	1.34	9.3E-04
DR76_RS02345	SoxR reducing system protein RseC	1.45	1.0E-03
DR76_RS02360	RNA polymerase sigma E factor	1.29	2.3E-02
DR76_RS02440	alpha-ketoglutarate transporter	2.61	6.7E-10
DR76_RS02500	soluble pyridine nucleotide transhydrogenase	1.87	2.1E-03
DR76_RS02655	transcriptional regulator	1.03	1.4E-02
DR76_RS02730	universal stress protein D	1.35	4.1E-03
DR76_RS02735	hypothetical protein	1.73	5.6E-05
DR76_RS02865	formate dehydrogenase accessory protein	1.26	2.1E-03
DR76_RS02880	formate dehydrogenase-O-2C Fe-S subunit	1.74	8.6E-03
DR76_RS02885	formate dehydrogenase-N subunit gamma	1.65	1.1E-02
DR76_RS03005	hypothetical protein	1.29	5.5E-03
DR76_RS03015	nitrogen regulation protein NR(II)	1.07	1.9E-02
DR76_RS03060	hypothetical protein	1.52	8.2E-04
DR76_RS03230	carboxymethylenebutenolidase	1.92	7.2E-06
DR76_RS03320	5-methyltetrahydropteroyltriglutamate--homocysteine methyltransferase	2.52	5.1E-09
DR76_RS03330	membrane protein	1.35	1.7E-03
DR76_RS03345	homoserine-2C homoserine lactone and S-methyl-methionine efflux pump	2.13	2.3E-07
DR76_RS03365	hypothetical protein	2.68	1.7E-09
DR76_RS03370	chloramphenical resistance permease RarD	1.17	6.0E-03
DR76_RS03390	membrane protein	1.64	2.1E-03
DR76_RS03415	tyrosine recombinase XerC	1.03	1.7E-02
DR76_RS03420	hypothetical protein	1.05	1.1E-02

DR76_RS03505	alanine glycine permease	1.56	1.7E-04
DR76_RS03575	transcription termination factor	1.86	1.5E-03
DR76_RS03580	rho operon leader peptide	1.94	8.3E-04
DR76_RS03635	acetolactate synthase 2 small subunit	1.32	1.1E-02
DR76_RS03645	ilvG operon leader peptide	1.44	1.3E-02
DR76_RS03735	low affinity potassium transport system protein kup	2.07	1.1E-06
DR76_RS03745	protoheme IX farnesyltransferase	1.06	2.4E-02
DR76_RS03750	asparagine synthetase AsnA	3.68	4.1E-09
DR76_RS03860	PTS beta-glucoside transporter subunit IIABC	1.21	1.1E-02
DR76_RS03970	DNA replication and repair protein RecF	1.12	7.3E-03
DR76_RS03985	hypothetical protein	1.11	1.5E-02
DR76_RS04040	heat shock protein IbpA	1.91	4.4E-06
DR76_RS04045	heat shock chaperone IbpB	2.80	2.7E-10
DR76_RS04070	hypothetical protein	1.39	2.2E-03
DR76_RS04100	acetolactate synthase	1.25	8.1E-03
DR76_RS04160	hypothetical protein	1.60	2.2E-03
DR76_RS04355	xanthine permease	1.94	1.2E-04
DR76_RS04370	tRNA methyltransferase	1.14	5.7E-03
DR76_RS04385	guanylate kinase	1.30	1.6E-02
DR76_RS04450	5-hydroxymethyluracil DNA glycosylase	1.06	1.5E-02
DR76_RS04510	ADP-heptose:LPS heptosyl transferase	1.20	5.5E-03
DR76_RS04540	hypothetical protein	1.01	1.5E-02
DR76_RS04590	transcriptional regulator	1.11	1.9E-02
DR76_RS04615	hypothetical protein	4.12	6.1E-15
DR76_RS04620	hypothetical protein	2.37	8.1E-09
DR76_RS04630	PTS mannitol transporter subunit IIABC	1.04	2.0E-02
DR76_RS04635	hypothetical protein	3.11	1.9E-08
DR76_RS04640	membrane protein	1.40	4.2E-03
DR76_RS04645	glutathione S-transferase	1.29	1.9E-03
DR76_RS04670	aldehyde dehydrogenase	2.30	1.2E-07
DR76_RS04740	valine--pyruvate aminotransferase	1.54	3.3E-03
DR76_RS04750	putative glucosaminidase	1.78	7.0E-04
DR76_RS04815	toxic polypeptide-2C small	1.21	2.4E-02
DR76_RS04820	cold shock protein CspA	1.50	1.5E-02
DR76_RS04825	XRE family transcriptional regulator	6.47	3.2E-32
DR76_RS04860	hypothetical protein	2.97	1.3E-12
DR76_RS04880	hypothetical protein	3.00	8.0E-09
DR76_RS04885	peptide ABC transporter substrate-binding protein	2.46	1.3E-07
DR76_RS04890	hypothetical protein	2.62	4.7E-07
DR76_RS04895	dipeptide/heme ABC transporter permease	2.12	4.9E-06
DR76_RS04900	dipeptide transport system permease protein DppC	1.84	1.3E-04
DR76_RS04905	peptide ABC transporter ATP-binding protein	1.51	8.0E-04
DR76_RS04915	transporter	1.12	1.2E-02
DR76_RS05000	hypothetical protein	1.39	2.2E-02

DR76_RS05010	hypothetical protein	1.76	4.4E-05
DR76_RS05015	transporter	1.73	6.2E-05
DR76_RS05020	membrane protein	3.52	2.9E-16
DR76_RS05030	LuxR family transcriptional regulator	2.34	5.3E-07
DR76_RS05035	trehalase	2.48	4.9E-06
DR76_RS05045	glutamate decarboxylase	5.82	6.3E-20
DR76_RS05050	AraC family transcriptional regulator	3.20	1.2E-07
DR76_RS05055	AraC family transcriptional regulator	3.80	9.5E-14
DR76_RS05060	multidrug resistance protein MdtF	4.28	6.1E-12
DR76_RS05065	multidrug transporter	5.12	1.1E-19
DR76_RS05070	hypothetical protein	5.93	5.3E-31
DR76_RS05075	gad regulon transcriptional activator	6.03	7.0E-19
DR76_RS05080	acid-resistance protein HdeD	5.81	8.3E-20
DR76_RS05085	acid-resistance protein HdeA	5.29	3.8E-08
DR76_RS05090	acid-resistance protein HdeB	5.16	4.4E-08
DR76_RS05095	magnesium transporter ATPase	6.25	1.3E-21
DR76_RS05145	LuxR family transcriptional regulator	6.81	1.1E-30
DR76_RS05150	membrane protein	4.68	5.2E-12
DR76_RS05165	hypothetical protein	3.26	1.7E-13
DR76_RS05200	universal stress (ethanol tolerance) protein B	4.53	6.2E-17
DR76_RS05305	pheromone autoinducer 2 transporter	3.10	2.9E-13
DR76_RS05320	membrane protein	1.24	6.5E-03
DR76_RS05395	branched-chain amino acid transporter permease subunit LivH	1.25	1.9E-02
DR76_RS05405	amino acid ABC transporter ATP-binding protein	1.27	1.1E-02
DR76_RS05415	hypothetical protein	2.97	8.1E-06
DR76_RS05420	glycerol-3-phosphate ABC transporter substrate-binding protein	2.35	1.3E-07
DR76_RS05425	sn-glycerol-3-phosphate transport system permease protein UgpA	2.73	3.2E-07
DR76_RS05430	glycerol-3-phosphate transporter membrane protein	2.91	2.8E-08
DR76_RS05440	cytoplasmic glycerophosphodiester phosphodiesterase	1.08	1.2E-02
DR76_RS05445	membrane protein	3.67	4.1E-12
DR76_RS05450	gamma-glutamyltranspeptidase	3.66	2.0E-16
DR76_RS05455	hypothetical protein	1.74	2.4E-03
DR76_RS05475	hypothetical protein	2.63	1.5E-06
DR76_RS05600	glycerol-3-phosphate dehydrogenase	2.54	3.1E-09
DR76_RS05605	thiosulfate:cyanide sulfurtransferase (rhodanese)	1.66	9.5E-05
DR76_RS05610	rhomboid intramembrane serine protease	1.41	7.0E-04
DR76_RS05670	transposase	1.26	1.3E-02
DR76_RS05725	hypothetical protein	1.89	1.3E-04
DR76_RS05740	ribosome-associated heat shock protein Hsp15	1.35	1.2E-03
DR76_RS05745	nucleotidase	1.31	1.9E-03
DR76_RS05805	DNA adenine methylase	1.03	1.8E-02
DR76_RS05865	membrane protein	4.18	2.6E-11
DR76_RS05890	putative transporter	1.63	2.3E-04
DR76_RS05900	hypothetical protein	4.17	1.5E-19

DR76_RS05905	cell filamentation protein Fic	4.17	1.6E-20
DR76_RS05910	anthranilate synthase subunit II	3.01	2.1E-12
DR76_RS05915	succinyldiaminopimelate aminotransferase	1.26	3.8E-03
DR76_RS06005	sulfur transfer complex subunit TusB	1.88	2.6E-05
DR76_RS06035	bacterioferritin-associated ferredoxin	2.61	1.1E-07
DR76_RS06040	bacterioferritin	5.86	2.8E-22
DR76_RS06045	methyltransferase	1.74	1.2E-04
DR76_RS06095	secretion system protein D	1.11	1.4E-02
DR76_RS06100	pilus assembly protein PilZ	3.15	1.4E-09
DR76_RS06255	hypothetical protein	1.10	1.1E-02
DR76_RS06260	zinc-responsive transcriptional regulator	1.20	5.5E-03
DR76_RS06265	putative regulator	1.28	4.6E-03
DR76_RS06270	large-conductance mechanosensitive channel	1.12	1.3E-02
DR76_RS06275	potassium transporter peripheral membrane component	1.14	1.6E-02
DR76_RS06310	tRNA(ANN) t(6)A37 threonylcarbamoyladenosine modification protein	1.34	4.7E-03
DR76_RS06315	shikimate 5-dehydrogenase	1.54	1.3E-03
DR76_RS06320	hypothetical protein	1.97	7.3E-06
DR76_RS06360	amino acid ABC transporter permease	1.44	2.9E-03
DR76_RS06365	amino acid ABC transporter substrate-binding protein	1.49	6.1E-04
DR76_RS06505	regulatory protein	1.39	2.0E-03
DR76_RS06525	rod shape-determining protein MreD	1.27	2.3E-03
DR76_RS06550	transcriptional regulator for aaeXAB operon	1.83	7.6E-06
DR76_RS06555	protein AaeX	1.59	7.2E-04
DR76_RS06560	hydroxybenzoic acid transporter	1.37	3.1E-03
DR76_RS06565	hydroxybenzoic acid transporter	1.16	8.4E-03
DR76_RS06570	putative barnase inhibitor	5.70	5.4E-31
DR76_RS06575	membrane protein	3.71	5.2E-15
DR76_RS06690	hypothetical protein	3.27	5.7E-09
DR76_RS06750	organic solvent ABC transporter ATP-binding protein	1.08	1.7E-02
DR76_RS06830	23S rRNA U2552 2"-O-ribose methyltransferase-2C SAM-dependent	1.55	7.9E-03
DR76_RS06930	putative luciferase-like monooxygenase	2.30	1.0E-04
DR76_RS07165	membrane protein	1.68	3.5E-05
DR76_RS07170	hypothetical protein	3.05	7.5E-12
DR76_RS07175	membrane protein	2.82	4.0E-11
DR76_RS07180	membrane protein	2.97	5.7E-10
DR76_RS07185	membrane protein	2.90	2.6E-09
DR76_RS07190	membrane protein	2.76	3.9E-07
DR76_RS07195	hypothetical protein	2.98	1.5E-07
DR76_RS07235	serine/threonine transporter	1.27	5.8E-03
DR76_RS07315	putrescine--2-oxoglutarate aminotransferase	5.27	3.4E-22
DR76_RS07400	tRNA nucleotidyl transferase	1.10	2.0E-02
DR76_RS07530	hypothetical protein	1.36	3.7E-03
DR76_RS07630	hypothetical protein	2.73	4.1E-08

DR76_RS07635	AraC family transcriptional regulator	1.55	2.6E-04
DR76_RS07640	ABC transporter substrate-binding protein	1.38	1.6E-03
DR76_RS07700	membrane protein	1.24	1.9E-02
DR76_RS07705	hypothetical protein	1.43	1.2E-03
DR76_RS07710	2-2C5-diketo-D-gluconic acid reductase	2.26	1.6E-06
DR76_RS07755	oxidoreductase	4.30	1.7E-19
DR76_RS07860	hypothetical protein	1.39	5.8E-03
DR76_RS07870	permease	1.58	1.0E-03
DR76_RS07875	permease	1.38	2.6E-03
DR76_RS07880	8-amino-7-oxononanoate synthase	1.54	9.8E-04
DR76_RS07910	glycolate oxidase subunit GlcD	6.75	8.4E-21
DR76_RS07915	FAD-binding protein	7.38	4.5E-25
DR76_RS07920	glycolate oxidase	6.46	7.1E-21
DR76_RS07925	DUF336 family protein	5.66	1.1E-17
DR76_RS07930	malate synthase G	5.37	1.8E-14
DR76_RS07935	glycolate permease glcA	5.53	1.9E-25
DR76_RS08095	hypothetical protein	1.87	1.9E-04
DR76_RS08350	hypothetical protein	2.45	3.2E-03
DR76_RS08355	hypothetical protein	1.78	1.8E-05
DR76_RS08360	integrase	2.50	4.6E-09
DR76_RS08890	mechanosensitive channel MscS	2.65	4.3E-08
DR76_RS08930	3-phosphoglycerate dehydrogenase	1.30	2.8E-03
DR76_RS09195	D-galactose transporter	1.25	4.4E-03
DR76_RS09225	lysophospholipid transporter LplT	1.15	9.6E-03
DR76_RS09500	DUF903 family verified lipoprotein	4.72	4.8E-15
DR76_RS09505	glycine cleavage system transcriptional activator	1.78	1.5E-05
DR76_RS09510	UPF0382 family inner membrane protein	1.42	1.0E-03
DR76_RS09585	hypothetical protein	1.35	2.3E-02
DR76_RS09590	hypothetical protein	1.22	9.2E-03
DR76_RS09595	tRNA pseudouridine synthase C	1.75	9.3E-05
DR76_RS09810	lipoprotein NlpD	1.33	2.5E-02
DR76_RS10070	pleiotropic regulatory protein for carbon source metabolism	2.03	3.6E-04
DR76_RS10100	hypothetical protein	1.95	5.4E-05
DR76_RS10150	branched-chain amino acid ABC transporter permease	1.30	1.6E-03
DR76_RS10155	membrane protein	1.11	1.5E-02
DR76_RS10160	transporter	2.25	1.2E-07
DR76_RS10180	ribonucleotide-diphosphate reductase subunit beta	2.19	1.0E-05
DR76_RS10185	ribonucleotide-diphosphate reductase subunit alpha	2.63	6.4E-09
DR76_RS10190	protein NrdI	2.69	1.4E-07
DR76_RS10195	hydrogen donor for NrdEF electron transport system-3B glutaredoxin-like protein	3.46	7.8E-11
DR76_RS10210	putative membrane-anchored DUF883 family ribosome-binding protein	6.43	1.2E-20
DR76_RS10215	uncharacterized protein	2.11	2.1E-06
DR76_RS10230	membrane protein	1.18	9.5E-03

DR76_RS10240	hypothetical protein	3.00	6.5E-10
DR76_RS10245	peptidoglycan-binding protein LysM	4.20	4.5E-17
DR76_RS10250	transcriptional regulator	2.99	7.4E-13
DR76_RS10255	gamma-aminobutyrate transporter	3.94	2.3E-16
DR76_RS10260	4-aminobutyrate aminotransferase	4.08	8.2E-19
DR76_RS10265	succinate-semialdehyde dehdyrogenase	4.39	5.2E-23
DR76_RS10270	hydroxyglutarate oxidase	4.70	1.3E-24
DR76_RS10275	carbon starvation induced protein	3.94	9.2E-17
DR76_RS10365	lipoprotein	2.36	9.0E-08
DR76_RS10390	hypothetical protein	1.78	6.5E-04
DR76_RS10450	hypothetical protein	3.22	8.1E-08
DR76_RS10580	hypothetical protein	2.50	3.3E-05
DR76_RS10585	zinc resistance protein	2.94	1.6E-02
DR76_RS10590	sensor protein ZraS	1.39	3.1E-03
DR76_RS10595	acetoacetate metabolism regulatory protein AtoC	1.17	7.7E-03
DR76_RS10630	hypothetical protein	1.32	4.1E-03
DR76_RS10635	acetyltransferase	1.57	3.1E-04
DR76_RS10645	malate synthase	4.54	4.4E-11
DR76_RS10650	isocitrate lyase	5.77	5.0E-12
DR76_RS10655	bifunctional isocitrate dehydrogenase kinase/phosphatase	4.47	2.2E-12
DR76_RS10735	hypothetical protein	1.46	3.6E-04
DR76_RS10740	aspartate kinase	1.37	2.2E-03
DR76_RS10770	putative periplasmic protein	1.44	5.1E-03
DR76_RS10780	phosphate-starvation-inducible protein PsiE	2.16	1.4E-06
DR76_RS10820	chorismate pyruvate lyase	1.80	3.3E-05
DR76_RS10825	p-hydroxybenzoate octaprenyltransferase	1.99	4.9E-06
DR76_RS10850	hypothetical protein	4.08	2.1E-12
DR76_RS10860	tRNA-dihydrouridine synthase A	1.48	2.7E-04
DR76_RS10980	thiamin phosphate synthase	1.71	1.4E-03
DR76_RS10985	hypothetical protein	1.76	4.7E-05
DR76_RS11000	membrane protein	2.05	3.0E-05
DR76_RS11050	membrane protein	1.33	6.0E-03
DR76_RS11055	acetyl-coenzyme A synthetase	2.19	4.1E-07
DR76_RS11100	hypothetical protein	1.43	1.1E-03
DR76_RS11200	putative periplasmic protein	1.89	1.3E-05
DR76_RS11205	phosphonate metabolism protein PhnP	1.41	1.4E-03
DR76_RS11210	aminoalkylphosphonic acid N-acetyltransferase	1.47	1.9E-03
DR76_RS11275	hypothetical protein	5.24	1.4E-27
DR76_RS11295	proline/glycine betaine transporter	2.12	3.4E-04
DR76_RS11300	putative membrane-bound BasS regulator	2.09	1.3E-05
DR76_RS11380	hypothetical protein	4.09	3.8E-18
DR76_RS11385	hypothetical protein	3.98	4.4E-18
DR76_RS11430	membrane protein	2.04	2.0E-04
DR76_RS11435	putative transcriptional regulator	1.69	4.6E-04

DR76_RS11460	exclusion suppressor FxsA	1.63	2.9E-04
DR76_RS11490	lysine 2-2C3-aminomutase	1.12	1.1E-02
DR76_RS11505	entericidin B membrane lipoprotein	5.73	1.7E-31
DR76_RS11510	quaternary ammonium compound-resistance protein SugE	1.57	2.6E-04
DR76_RS11515	outer membrane lipoprotein Blc	4.16	1.4E-19
DR76_RS11585	iron-sulfur cluster binding protein	1.38	2.7E-03
DR76_RS11590	carbohydrate kinase	1.08	1.1E-02
DR76_RS11635	membrane protein	1.43	2.9E-03
DR76_RS11690	isovaleryl CoA dehydrogenase	4.73	2.8E-18
DR76_RS11750	membrane protein	2.48	1.6E-07
DR76_RS11815	3-ketoacyl-ACP reductase	2.00	8.4E-04
DR76_RS11845	membrane protein	1.72	3.6E-05
DR76_RS11865	3'-5'-bisphosphate nucleotidase	1.92	3.8E-05
DR76_RS11875	DUF1107 family protein	2.06	1.2E-04
DR76_RS12100	alcohol dehydrogenase	4.25	6.4E-19
DR76_RS12180	iron ABC transporter	1.24	4.4E-03
DR76_RS12185	Fe3+ dicitrate ABC transporter permease	1.40	1.7E-03
DR76_RS12190	Fe(3+) dicitrate transport system permease protein FecC	1.16	1.3E-02
DR76_RS12205	fec operon regulator FecR	2.33	7.4E-08
DR76_RS12210	RNA polymerase sigma factor	2.69	6.7E-10
DR76_RS12215	transposase	2.45	5.9E-09
DR76_RS12520	9-O-acetyl-N-acetylneuraminic acid deacetylase	2.14	7.6E-06
DR76_RS12525	N-acetylneuraminic acid mutarotase	1.99	7.2E-06
DR76_RS12570	type-1 fimbrial protein subunit A	3.22	2.0E-11
DR76_RS12575	fimbrin fimI	3.13	1.1E-09
DR76_RS12580	molecular chaperone FimC	2.37	5.0E-05
DR76_RS12600	fimbrial protein FimH	1.97	2.2E-05
DR76_RS12650	cell density-dependent motility repressor	1.03	1.4E-02
DR76_RS12660	SpmB family inner membrane protein	2.30	1.9E-06
DR76_RS12665	nucleoside recognition pore and gate family putative inner membrane transporter	2.60	3.4E-08
DR76_RS12675	hypothetical protein	1.94	3.8E-06
DR76_RS12700	membrane protein	2.79	7.1E-09
DR76_RS12705	multidrug transporter	1.32	8.8E-03
DR76_RS12715	GntR family transcriptional regulator	1.31	2.2E-03
DR76_RS12785	L-galactonate transporter	1.29	2.2E-03
DR76_RS12840	ferric iron reductase involved in ferric hydroximate transport	2.64	8.2E-10
DR76_RS12885	dUMP phosphatase	1.42	4.1E-04
DR76_RS12895	hypothetical protein	7.25	2.3E-23
DR76_RS12900	uncharacterized protein	7.06	2.2E-24
DR76_RS12905	putative patatin-like family phospholipase	2.91	2.2E-10
DR76_RS12910	deoxyribonuclease	1.93	1.6E-05
DR76_RS12925	hypothetical protein	2.53	1.4E-06
DR76_RS12975	nicotinamide-nucleotide adenylyltransferase	1.43	8.3E-04
DR76_RS13025	sensory histidine kinase	1.05	2.2E-02

DR76_RS13050	thr operon leader peptide	1.09	1.5E-02
DR76_RS13055	bifunctional aspartokinase I/homoserine dehydrogenase I	1.90	5.6E-03
DR76_RS13060	homoserine kinase	2.32	1.9E-03
DR76_RS13065	threonine synthase	1.85	9.6E-03
DR76_RS13070	hypothetical protein	1.59	1.1E-02
DR76_RS13165	hypothetical protein	1.08	1.6E-02
DR76_RS13215	hypothetical protein	2.29	9.2E-03
DR76_RS13300	potassium transporter	1.11	1.2E-02
DR76_RS13320	diadenosine tetraphosphatase	1.33	2.3E-03
DR76_RS13325	cobalt transporter	1.54	7.1E-04
DR76_RS13330	dimethyladenosine transferase	1.35	1.4E-02
DR76_RS13335	4-hydroxythreonine-4-phosphate dehydrogenase	1.43	8.9E-03
DR76_RS13355	23S rRNA/tRNA pseudouridine synthase A	1.60	9.0E-05
DR76_RS13360	ATP-dependent helicase	1.56	2.8E-03
DR76_RS13400	membrane protein	1.23	4.5E-03
DR76_RS13560	secretion monitor	1.19	2.5E-02
DR76_RS13735	glucose dehydrogenase	3.97	1.7E-11
DR76_RS13745	carbonic anhydrase	1.14	2.4E-02
DR76_RS13895	chloride channel protein	1.74	9.0E-05
DR76_RS14040	lysine decarboxylase CadA	2.15	3.8E-06
DR76_RS14045	lyase	1.71	2.6E-05
DR76_RS14160	2-2C5-diketo-D-gluconic acid reductase	2.40	4.6E-07
DR76_RS14170	endo/exonuclease/phosphatase family protein	1.27	1.6E-02
DR76_RS14175	S-adenosyl-L-methionine (SAM)-dependent methyltransferase PhcB	1.47	4.4E-04
DR76_RS14535	cobalamin synthase	1.16	7.2E-03
DR76_RS14540	nicotinate-nucleotide--dimethylbenzimidazole phosphoribosyltransferase	1.68	3.8E-05
DR76_RS14545	L-2CD-transpeptidase	4.13	1.4E-17
DR76_RS14675	MATE efflux family protein	1.55	2.9E-04
DR76_RS14690	LysR family transcriptional regulator	1.23	1.5E-02
DR76_RS14695	hypothetical protein	3.96	1.7E-09
DR76_RS14730	adhesin	3.39	1.8E-04
DR76_RS14940	hypothetical protein	3.33	2.2E-07
DR76_RS14955	TMAO/DMSO reductase	1.30	2.8E-03
DR76_RS14970	sensor histidine kinase	1.46	1.6E-03
DR76_RS14975	heat shock protein HSP31	2.21	6.7E-07
DR76_RS14980	hypothetical protein	3.22	7.0E-11
DR76_RS14985	outer membrane protein F	1.30	2.5E-03
DR76_RS14995	permease	2.09	3.5E-06
DR76_RS15000	hydrolase	1.91	2.7E-06
DR76_RS15025	hypothetical protein	2.81	4.1E-11
DR76_RS15030	diguanylate cyclase	2.18	4.7E-07
DR76_RS15035	mannosyl-3-phosphoglycerate phosphatase	5.44	2.8E-25
DR76_RS15040	uncharacterized protein	6.12	2.5E-19

DR76_RS15045	uncharacterized protein	1.08	2.1E-02
DR76_RS15155	hypothetical protein	3.13	2.7E-12
DR76_RS15175	alpha-amylase	2.07	5.1E-06
DR76_RS15300	hypothetical protein	1.59	3.0E-03
DR76_RS15330	trehalose-6-phosphate phosphatase	7.49	5.3E-26
DR76_RS15335	trehalose-6-phosphate synthase	7.04	5.2E-24
DR76_RS15340	universal stress protein C	2.07	7.6E-07
DR76_RS15440	trimethylamine N-oxide reductase I catalytic subunit	1.35	5.6E-03
DR76_RS15490	hypothetical protein	3.28	8.1E-11
DR76_RS15590	peptidase S9	1.44	7.5E-04
DR76_RS15605	DNA polymerase III-2C theta subunit	1.37	2.3E-03
DR76_RS15630	serine/threonine protein phosphatase	4.19	4.9E-21
DR76_RS15635	hypothetical protein	3.61	8.8E-16
DR76_RS15640	uncharacterized protein	7.81	3.0E-27
DR76_RS15650	hypothetical protein	1.64	1.8E-03
DR76_RS15655	membrane protein	2.08	1.4E-06
DR76_RS15690	uncharacterized protein	1.37	2.7E-03
DR76_RS15790	hypothetical protein	1.37	1.2E-03
DR76_RS15855	membrane protein	4.23	3.1E-16
DR76_RS15915	hypothetical protein	5.74	5.5E-21
DR76_RS15920	protein kinase-2C endogenous substrate unidentified-3B autokinase	5.88	1.7E-17
DR76_RS15995	nicotinamidase/pyrazinamidase	2.34	1.4E-08
DR76_RS16000	cytoplasmic asparaginase I	1.06	9.8E-03
DR76_RS16115	succinylornithine transaminase	1.90	9.4E-05
DR76_RS16120	arginine succinyltransferase	2.14	1.1E-05
DR76_RS16125	succinylglutamate-semialdehyde dehydrogenase	1.60	8.4E-04
DR76_RS16135	succinylglutamate desuccinylase	1.83	7.1E-05
DR76_RS16140	hypothetical protein	1.20	1.3E-02
DR76_RS16165	transcriptional regulator	3.37	1.2E-08
DR76_RS16200	hydroperoxidase II	5.32	2.8E-18
DR76_RS16225	membrane protein	1.42	9.5E-04
DR76_RS16235	hypothetical protein	1.76	1.5E-05
DR76_RS16255	membrane protein	1.20	2.2E-02
DR76_RS16295	integration host factor subunit alpha	1.54	6.8E-03
DR76_RS16310	vitamin B12 import ATP-binding protein BtuD	1.09	8.5E-03
DR76_RS16320	cyclic di-GMP regulator CdgR	1.87	3.3E-04
DR76_RS16325	hypothetical protein	1.30	2.3E-03
DR76_RS16425	membrane protein	1.61	1.1E-04
DR76_RS16440	uncharacterized protein	3.50	6.5E-09
DR76_RS16445	iron-sulfur cluster assembly scaffold protein	4.56	2.4E-13
DR76_RS16450	cysteine desulfurase activator complex subunit SufB	4.20	2.3E-14
DR76_RS16455	cysteine desulfurase	4.27	2.5E-17
DR76_RS16460	cysteine desulfurase activator complex subunit SufD	4.22	4.1E-18
DR76_RS16465	cysteine desulfurase	4.11	2.0E-19

DR76_RS16470	cysteine desufuration protein SufE	4.37	7.7E-23
DR76_RS16475	murein L-2CD-transpeptidase	3.37	3.9E-08
DR76_RS16485	pyruvate kinase	1.94	1.5E-03
DR76_RS16490	uncharacterized protein	1.06	1.4E-02
DR76_RS16525	hypothetical protein	3.68	4.5E-13
DR76_RS16530	monooxygenase	1.49	8.4E-04
DR76_RS16545	hypothetical protein	1.67	1.5E-03
DR76_RS16560	cyclopropane-fatty-acyl-phospholipid synthase	2.09	2.0E-04
DR76_RS16580	stress response membrane	1.54	1.2E-03
DR76_RS16585	MFS transporter	1.62	1.5E-04
DR76_RS16630	hypothetical protein	1.33	5.2E-03
DR76_RS16640	superoxide dismutase	2.70	6.9E-11
DR76_RS16645	fusaric acid resistance protein	2.44	4.6E-09
DR76_RS16655	membrane protein	1.58	5.2E-04
DR76_RS16705	hypothetical protein	1.63	1.7E-03
DR76_RS16805	porin	1.18	2.5E-02
DR76_RS16810	hypothetical protein	1.85	1.8E-03
DR76_RS16825	fumarate hydratase	1.19	1.2E-02
DR76_RS16830	DNA-binding protein	1.50	5.2E-04
DR76_RS16845	membrane protein	2.34	3.6E-07
DR76_RS16850	dihydromonapterin reductase	2.51	5.1E-10
DR76_RS16890	serine protease	1.92	4.8E-05
DR76_RS16900	acid-shock protein	1.21	9.2E-03
DR76_RS16905	membrane protein	3.39	4.5E-12
DR76_RS16975	membrane protein	1.17	1.3E-02
DR76_RS16990	hypothetical protein	2.04	2.0E-06
DR76_RS16995	DNA-binding protein	1.83	4.2E-04
DR76_RS17285	hypothetical protein	1.04	1.8E-02
DR76_RS17305	hypothetical protein	2.40	1.4E-06
DR76_RS17325	transporter	1.45	6.8E-04
DR76_RS17330	O-acetylserine/cysteine export protein	1.35	1.1E-03
DR76_RS17380	membrane protein	1.28	2.1E-03
DR76_RS17385	arabinose transporter	1.72	4.1E-05
DR76_RS17410	diguanylate cyclase	1.07	9.9E-03
DR76_RS17425	hypothetical protein	2.66	4.8E-08
DR76_RS17430	trans-aconitate 2-methyltransferase	3.40	2.8E-15
DR76_RS17435	antitoxin	1.24	1.1E-02
DR76_RS17530	zinc protease	1.46	1.0E-03
DR76_RS17540	glutamate decarboxylase	6.12	3.3E-11
DR76_RS17545	antiporter	7.41	5.2E-21
DR76_RS17565	peroxiredoxin	1.59	3.6E-03
DR76_RS17570	biofilm-dependent modulation protein	1.34	2.1E-03
DR76_RS17575	30S ribosomal protein S22	2.22	7.7E-05
DR76_RS17585	acetaldehyde reductase	1.75	4.9E-05

DR76_RS17595	putative DNA-binding transcriptional regulator	1.04	1.8E-02
DR76_RS17625	nitrite extrusion protein 2	5.37	4.8E-28
DR76_RS17630	nitrate reductase A subunit alpha	4.89	5.0E-16
DR76_RS17635	nitrate reductase A subunit beta	4.28	1.4E-20
DR76_RS17640	nitrate reductase molybdenum cofactor assembly chaperone NarJ	3.59	4.9E-15
DR76_RS17660	hypothetical protein	2.00	3.3E-05
DR76_RS17735	transferase	6.04	7.6E-15
DR76_RS17740	glutathione S-transferase	5.20	6.0E-21
DR76_RS17750	hypothetical protein	1.46	2.0E-03
DR76_RS17755	TonB-dependent receptor	3.42	6.3E-13
DR76_RS17785	hypothetical protein	3.51	1.8E-14
DR76_RS17790	hypothetical protein	2.57	2.1E-09
DR76_RS17795	spermidine/putrescine ABC transporter permease	3.36	1.3E-10
DR76_RS17800	spermidine/putrescine ABC transporter permease	2.89	1.0E-09
DR76_RS17805	polyamine ABC transporter ATP-binding protein	3.75	6.9E-14
DR76_RS17810	spermidine/putrescine ABC transporter substrate-binding protein	3.87	4.8E-18
DR76_RS17815	GntR family transcriptional regulator	1.01	1.8E-02
DR76_RS17820	antitoxin	3.75	9.5E-12
DR76_RS17860	acetyltransferase	2.28	3.2E-07
DR76_RS17885	hypothetical protein	1.30	2.4E-03
DR76_RS17890	LysR family transcripitonal regulator	1.79	1.5E-04
DR76_RS17915	glyceraldehyde-3-phosphate dehydrogenase	3.70	4.1E-17
DR76_RS18000	pyruvate-flavodoxin oxidoreductase	1.79	1.4E-03
DR76_RS18035	RNA helicase	1.40	1.7E-03
DR76_RS18040	hypothetical protein	4.63	2.7E-21
DR76_RS18050	diguanylate cyclase	2.09	2.8E-06
DR76_RS18135	membrane protein	1.28	1.8E-03
DR76_RS18140	L-Ala-D/L-Glu epimerase	1.21	5.5E-03
DR76_RS18160	hypothetical protein	1.93	9.0E-04
DR76_RS18185	glycosyl hydrolase family 65	1.06	2.4E-02
DR76_RS18190	oxidoreductase	1.34	1.8E-02
DR76_RS18230	phage-shock protein	1.72	5.2E-04
DR76_RS18235	transcriptional regulator	1.63	2.0E-04
DR76_RS18240	psp operon transcription co-activator	1.98	3.8E-06
DR76_RS18245	phage-shock protein	1.39	1.5E-03
DR76_RS18265	membrane protein	1.14	1.6E-02
DR76_RS18275	peptide ABC transporter permease	1.28	1.8E-03
DR76_RS18280	peptide ABC transporter permease	1.09	9.6E-03
DR76_RS18285	peptide ABC transporter ATP-binding protein	1.54	1.6E-04
DR76_RS18290	peptide ABC transporter ATP-binding protein	1.01	2.4E-02
DR76_RS18340	RNase II stability modulator	1.79	2.1E-05
DR76_RS18360	translation initiation factor Sui1	2.90	9.8E-09
DR76_RS18390	aconitate hydratase	3.10	2.5E-07
DR76_RS18500	hypothetical protein	4.35	5.2E-16

DR76_RS18530	transporter	1.47	4.5E-04
DR76_RS18615	response regulator	1.79	1.0E-03
DR76_RS18620	hypothetical protein	1.47	5.8E-03
DR76_RS18720	invasin	1.98	1.7E-06
DR76_RS18730	transporter	2.32	1.2E-08
DR76_RS18735	cation transport regulator	2.32	2.9E-07
DR76_RS18840	trehalase	2.59	1.2E-09
DR76_RS18875	hypothetical protein	5.98	1.0E-24
DR76_RS18910	SpoVR family protein	5.26	6.0E-17
DR76_RS18945	hypothetical protein	1.49	3.0E-04
DR76_RS18965	hypothetical protein	1.31	2.7E-03
DR76_RS18985	ATPase	2.75	3.1E-07
DR76_RS19030	diguanylate phosphodiesterase	1.53	1.3E-03
DR76_RS19050	two-component-system connector protein YcgZ	1.28	3.5E-03
DR76_RS19055	diguanylate phosphodiesterase	3.58	3.4E-10
DR76_RS20000	multiple stress resistance protein BhsA	1.29	5.1E-03
DR76_RS20030	thiamine kinase	1.48	2.3E-04
DR76_RS20055	fused glucose-specific PTS enzymes: IIB component/IIC component	1.56	1.3E-03
DR76_RS20060	DNAse	2.21	2.4E-05
DR76_RS20240	glutaredoxin	1.83	1.6E-03
DR76_RS20250	dihydroorotase	1.29	4.2E-03
DR76_RS20275	cytochrome B561	1.51	1.6E-03
DR76_RS20295	multidrug resistance protein MdtG	2.70	1.8E-08
DR76_RS20305	SecY/SecA suppressor protein	3.71	1.7E-14
DR76_RS20310	lipoprotein	3.01	2.5E-12
DR76_RS20340	hypothetical protein	1.49	8.0E-04
DR76_RS20345	RNase III inhibitor	1.18	3.6E-03
DR76_RS20350	hypothetical protein	1.72	4.6E-04
DR76_RS20355	curli assembly protein CsgC	2.07	3.5E-04
DR76_RS20375	transcriptional regulator	2.28	4.7E-07
DR76_RS20380	curlin secretion specificity factor	1.84	1.3E-04
DR76_RS20385	curli assembly protein CsgF	2.22	4.0E-05
DR76_RS20390	transporter	1.81	2.6E-05
DR76_RS20415	hypothetical protein	3.00	5.0E-07
DR76_RS20990	poly-beta-1-2C6-N-acetyl-D-glucosamine export protein	1.89	5.5E-04
DR76_RS20995	outer membrane N-deacetylase	2.00	5.3E-04
DR76_RS21000	N-glycosyltransferase	1.83	3.8E-03
DR76_RS21020	iron ABC transporter substrate-binding protein	1.14	1.2E-02
DR76_RS21035	hypothetical protein	1.54	2.5E-03
DR76_RS21040	bifunctional proline dehydrogenase/pyrroline-5-carboxylate dehydrogenase	1.71	1.9E-03
DR76_RS21055	amidohydrolase	2.23	2.8E-04
DR76_RS21060	putative aminoacrylate deaminase-2C reactive intermediate detoxification-3B weak enamine/imine deaminase activity	1.98	1.4E-03
DR76_RS21065	aminoacrylate hydrolase	2.61	1.6E-06

DR76_RS21070	malonic semialdehyde reductase	2.94	2.9E-08
DR76_RS21075	FMN reductase	3.20	1.1E-06
DR76_RS21080	pyrimidine permease	2.05	1.1E-05
DR76_RS21085	KGG family protein	4.54	4.3E-17
DR76_RS21090	hypothetical protein	3.11	7.9E-09
DR76_RS21095	NAD(P)H:quinone oxidoreductase	2.87	3.1E-06
DR76_RS21100	uncharacterized protein	2.96	2.1E-06
DR76_RS21110	DNA-binding protein	1.92	4.6E-04
DR76_RS21115	chaperone modulatory protein CbpM	1.35	1.0E-03
DR76_RS21120	chaperone protein TorD	1.10	1.7E-02
DR76_RS21175	periplasmic AppA protein	3.50	1.6E-09
DR76_RS21180	putative cytochrome bd-II oxidase subunit	4.56	1.0E-21
DR76_RS21185	cytochrome BD oxidase subunit II	5.76	1.8E-21
DR76_RS21190	cytochrome BD oxidase subunit I	5.88	4.0E-20
DR76_RS21195	hydrogenase-1 operon protein HyaF	7.25	5.4E-27
DR76_RS21200	hydrogenase-1 operon protein HyaE	6.68	3.1E-37
DR76_RS21205	hydrogenase 1 maturation protease	6.51	8.1E-27
DR76_RS21210	hydrogenase 1 b-type cytochrome subunit	6.63	1.5E-28
DR76_RS21215	hydrogenase 2 large subunit	7.12	2.2E-25
DR76_RS21220	hydrogenase 1-2C small subunit	7.35	2.1E-27
DR76_RS21240	acylphosphatase	1.73	3.7E-05
DR76_RS21245	ribosomal RNA large subunit methyltransferase I	1.15	7.1E-03
DR76_RS21250	heat shock protein HspQ	2.00	1.7E-04
DR76_RS21255	hypothetical protein	2.22	2.1E-06
DR76_RS21260	hypothetical protein	2.08	2.0E-04
DR76_RS21265	UPF0319 family protein	2.49	1.4E-08
DR76_RS21325	ribosome modulation factor	2.05	8.2E-04
DR76_RS21330	hypothetical protein	1.64	6.2E-05
DR76_RS21335	paraquat-inducible protein B	1.78	5.9E-05
DR76_RS21340	paraquat-inducible-2C SoxRS-regulated inner membrane protein	1.95	2.2E-06
DR76_RS21360	cell division protein ZapC	1.04	1.4E-02
DR76_RS21425	hypothetical protein	1.25	2.4E-02
DR76_RS21430	M15A protease-related family periplasmic protein	1.46	8.6E-03
DR76_RS21435	murein L-2CD-transpeptidase	1.95	3.6E-04
DR76_RS21480	hypothetical protein	1.15	6.6E-03
DR76_RS21530	membrane protein	4.03	1.4E-20
DR76_RS21560	hypothetical protein	3.81	1.2E-17
DR76_RS21640	ATP-dependent Clp protease adaptor protein ClpS	1.11	1.0E-02
DR76_RS21695	pyruvate dehydrogenase	5.49	1.5E-16
DR76_RS21715	N-acetylmuramoyl-L-alanine amidase	1.27	2.0E-03
DR76_RS21730	arginine transport ATP-binding protein ArtP	1.97	1.9E-06
DR76_RS21735	arginine ABC transporter substrate-binding protein	1.61	3.4E-04
DR76_RS21740	arginine ABC transporter permease	2.24	5.0E-08
DR76_RS21745	arginine ABC transporter permease	2.36	1.1E-08

DR76_RS21765	putrescine ABC transporter permease	1.04	2.5E-02
DR76_RS21795	ribosomal protein S6 modification protein	1.33	1.5E-03
DR76_RS21800	nitroreductase A	1.75	2.1E-05
DR76_RS21805	hypothetical protein	1.70	1.7E-04
DR76_RS21860	D-alanyl-D-alanine carboxypeptidase	1.22	6.2E-03
DR76_RS21870	aldose dehydrogenase	5.41	4.6E-19
DR76_RS21875	biofilm formation regulatory protein BssR	6.52	2.0E-09
DR76_RS21970	Mn(2)-response protein-2C MntR-repressed	1.75	1.6E-03
DR76_RS21975	phosphoethanolamine transferase	2.54	1.4E-07
DR76_RS21990	DNA protection during starvation protein	3.09	4.8E-07
DR76_RS22020	mechanosensitive channel protein	3.33	2.9E-15
DR76_RS22025	23S rRNA methyltransferase	1.06	1.3E-02
DR76_RS22030	hypothetical protein	3.26	4.1E-13
DR76_RS22045	hypothetical protein	2.06	8.8E-06
DR76_RS22050	hypothetical protein	2.17	6.4E-08
DR76_RS22110	hypothetical protein	5.01	1.1E-22
DR76_RS22115	cardiolipin synthase 2	5.11	6.0E-29
DR76_RS22120	membrane protein	4.32	7.5E-22
DR76_RS22125	hypothetical protein	1.58	1.1E-02
DR76_RS22130	putative acetate transporter-3B BAX Inhibitor-1 family inner membrane protein	1.11	1.2E-02
DR76_RS22205	kinase inhibitor protein	2.39	1.6E-08
DR76_RS22220	membrane protein	1.50	6.4E-03
DR76_RS22225	putative PrpF family isomerase	2.23	9.9E-05
DR76_RS22260	membrane protein	1.97	1.8E-05
DR76_RS22310	glutamate decarboxylase	4.03	1.1E-18
DR76_RS22315	hypothetical protein	6.99	9.8E-24
DR76_RS22320	zinc transporter ZitB	2.11	4.8E-07
DR76_RS22470	succinate dehydrogenase cytochrome b556 small membrane subunit	1.43	3.7E-03
DR76_RS22500	endoribonuclease SymE	1.06	1.1E-02
DR76_RS22630	hypothetical protein	1.05	2.4E-02
DR76_RS22715	asparagine synthetase B	3.48	3.5E-08
DR76_RS22720	hypothetical protein	2.33	1.5E-05
DR76_RS22770	endoribonuclease YbeY	1.06	1.5E-02
DR76_RS22780	apolipoprotein N-acyltransferase	1.21	7.9E-03
DR76_RS22795	glutamate/aspartate transport system permease protein GltJ	1.63	3.4E-03
DR76_RS22800	glutamate/aspartate ABC transporter permease	1.48	7.7E-03
DR76_RS22805	arginine ABC transporter ATP-binding protein	1.46	5.0E-03
DR76_RS22880	octanoyltransferase-3B octanoyl-[ACP]:protein N-octanoyltransferase	1.32	1.3E-03
DR76_RS22905	hydrolase	1.48	6.3E-04
DR76_RS22910	camphor resistance protein CrcB	2.41	2.2E-07
DR76_RS22920	phospholipid:lipid A palmitoyltransferase	2.58	6.3E-10
DR76_RS22975	ribonuclease I	1.17	6.9E-03
DR76_RS23000	disulfide isomerase	1.44	3.7E-03

DR76_RS23060	isochorismate synthase EntC	1.34	5.7E-03
DR76_RS23065	ferrienterobactin ABC transporter periplasmic binding protein	1.80	5.0E-05
DR76_RS23075	iron-enterobactin transporter membrane protein	1.87	4.2E-05
DR76_RS23085	iron-enterobactin transporter ATP-binding protein	1.42	2.8E-03
DR76_RS23140	gamma-glutamyl:cysteine ligase	4.03	1.1E-18
DR76_RS23145	membrane protein	1.96	3.5E-05
DR76_RS23200	general secretory system II-2C protein E protein	1.39	1.2E-03
DR76_RS23285	N5-carboxyaminoimidazole ribonucleotide mutase	2.15	3.3E-07
DR76_RS23290	phosphoribosylaminoimidazole carboxylase	1.70	5.6E-04
DR76_RS23330	glycerate kinase	2.18	3.8E-06
DR76_RS23335	purine permease	3.08	3.3E-10
DR76_RS23435	transcriptional regulator	2.00	5.4E-07
DR76_RS23440	amino acid permease	5.18	1.9E-22
DR76_RS23445	glutaminase	6.99	9.3E-36
DR76_RS23475	Fosmidomycin resistance protein	1.18	7.3E-03
DR76_RS23510	gap repair protein	1.20	7.6E-03
DR76_RS23520	DNA polymerase III subunit gamma/tau	1.58	3.5E-03
DR76_RS23530	membrane protein	2.19	4.4E-07
DR76_RS23540	hypothetical protein	1.11	2.3E-02
DR76_RS23545	hypothetical protein	1.40	2.0E-03
DR76_RS23555	HTH-type transcriptional regulator AcrR	1.38	1.5E-03
DR76_RS23570	Hha toxicity attenuator-3B conjugation-related protein	1.36	1.3E-02
DR76_RS23575	gene expression modulator	1.24	1.4E-02
DR76_RS23585	membrane protein	1.47	2.0E-03
DR76_RS23590	hypothetical protein	2.04	5.7E-06
DR76_RS23595	DUF1428 family protein	3.29	1.3E-13
DR76_RS23600	methylated-DNA--protein-cysteine methyltransferase	1.66	5.6E-05
DR76_RS23605	hypothetical protein	4.74	4.9E-15
DR76_RS23655	thioesterase	2.85	5.1E-11
DR76_RS23700	BolA family transcriptional regulator	3.20	4.3E-09
DR76_RS23720	cytochrome O ubiquinol oxidase	1.37	2.5E-02
DR76_RS23725	cytochrome O ubiquinol oxidase	1.49	1.1E-02
DR76_RS23730	cytochrome O ubiquinol oxidase	1.51	5.1E-03
DR76_RS23785	phosphatidylglycerophosphatase A	1.01	1.9E-02
DR76_RS23790	thiamine monophosphate kinase	1.14	6.2E-03
DR76_RS23815	hypothetical protein	5.16	4.4E-26
DR76_RS23870	branched-chain amino acid transporter 2 carrier protein BrnQ	1.63	2.4E-03
DR76_RS23890	exonuclease subunit SbcC	1.48	2.3E-03
DR76_RS23910	hypothetical protein	2.26	1.9E-07
DR76_RS23920	hypothetical protein	2.12	8.1E-06
DR76_RS23925	hypothetical protein	2.82	1.2E-06
DR76_RS23930	hypothetical protein	2.54	6.0E-07
DR76_RS23950	diguanylate cyclase-2C cellulose regualtor	1.41	1.3E-03
DR76_RS23955	PsiF family protein	4.24	5.3E-22

DR76_RS23995	hypothetical protein	1.27	6.6E-03
DR76_RS24130	acetyl-CoA synthetase	3.15	1.5E-11
DR76_RS24135	2-methylcitrate dehydratase	3.67	8.3E-17
DR76_RS24140	methylcitrate synthase	4.49	4.9E-22
DR76_RS24145	2-methylisocitrate lyase	4.33	1.6E-17
DR76_RS24150	propionate catabolism operon regulatory protein PrpR	1.26	1.1E-02
DR76_RS24155	hypothetical protein	4.31	1.1E-22
DR76_RS24170	zinc-binding dehydrogenase	3.54	1.2E-13
DR76_RS24175	deaminase	1.02	2.2E-02
DR76_RS24200	hypothetical protein	1.62	6.2E-03
DR76_RS24205	ankyrin	1.41	4.0E-03
DR76_RS24215	LysR family transcriptional regulator	1.41	1.0E-03
DR76_RS24225	hypothetical protein	1.56	7.6E-03
DR76_RS24275	membrane protein	2.18	9.5E-06
DR76_RS24475	outer membrane phosphoporin protein E	1.73	5.1E-05
DR76_RS24535	transpeptidase	1.14	7.4E-03
DR76_RS24550	acyl-CoA dehydrogenase	1.38	2.0E-02
DR76_RS24960	membrane protein	1.72	2.2E-05
DR76_RS24965	DNA gyrase inhibitor	2.15	2.7E-07
DR76_RS25000	LysR family transcripitonal regulator	1.07	1.5E-02
DR76_RS25065	UDP-glucose 6-dehydrogenase	2.91	4.4E-10
DR76_RS25125	colanic acid biosynthesis protein	1.47	2.1E-03
DR76_RS25225	membrane protein	1.23	2.5E-02
DR76_RS25245	diguanylate cyclase	1.10	2.4E-02
DR76_RS25305	hypothetical protein	4.90	1.6E-22
DR76_RS25345	lipid kinase	1.99	9.2E-07
DR76_RS25385	Fructose-bisphosphate aldolase class 1	2.04	4.5E-04
DR76_RS25460	hypothetical protein	1.34	1.6E-02
DR76_RS25465	tail fiber assembly protein	2.39	2.6E-07
DR76_RS25775	resolvase domain-containing protein	1.54	3.8E-04

Supplementary Table 2: Down-regulated genes resulted from differential gene analysis of *E.coli* 381 and ATCC 25922

Gene ID	Gene description	log2 FC	P-value
DR76_RS00025	lysozyme	-1.60	1.61E-04
DR76_RS00035	hydroxyethylthiazole kinase	-1.37	9.29E-05
DR76_RS00070	hypothetical protein	-1.65	1.82E-04
DR76_RS00105	hypothetical protein	-3.89	2.67E-05
DR76_RS00110	hypothetical protein	-16.84	6.57E-43
DR76_RS00115	hypothetical protein	-2.36	1.24E-04
DR76_RS00220	uncharacterized protein	-2.96	1.38E-07
DR76_RS00255	dihydropyrimidine dehydrogenase subunit A	-3.28	9.00E-12
DR76_RS00260	dihydropyrimidine dehydrogenase subunit B	-2.83	2.66E-09
DR76_RS00265	beta-methylgalactoside transporter	-2.77	1.02E-11

DR76_RS00270	D-ribose transporter ATP binding protein	-2.36	1.10E-07
DR76_RS00275	methyl-galactoside transporter subunit	-1.98	8.08E-07
DR76_RS00280	transcriptional regulator	-1.52	2.48E-05
DR76_RS00340	carbohydrate kinase	-2.38	7.27E-10
DR76_RS00345	ribonucleoside hydrolase	-4.61	4.63E-19
DR76_RS00350	Crp/Fnr family transcriptional regulator	-1.25	4.94E-03
DR76_RS00360	pseudouridine-5'-phosphate glycosidase	-7.32	9.87E-16
DR76_RS00365	pseudouridine kinase	-16.84	4.35E-21
DR76_RS00380	PTS fructose transporter subunit IIA	-1.37	2.84E-04
DR76_RS00470	Nucleoid-associated protein YejK	-1.01	3.95E-03
DR76_RS00485	hypothetical protein	-4.72	2.68E-17
DR76_RS00490	integrase	-16.84	2.82E-25
DR76_RS00495	hypothetical protein	-16.84	4.72E-54
DR76_RS00500	regulatory protein	-16.84	4.85E-18
DR76_RS00505	hypothetical protein	-16.84	4.37E-39
DR76_RS00510	hypothetical protein	-16.84	3.80E-09
DR76_RS00515	hypothetical protein	-16.84	3.73E-08
DR76_RS00520	hypothetical protein	-16.84	1.25E-04
DR76_RS00525	hypothetical protein	-16.84	9.51E-03
DR76_RS00540	Bacteriophage P4 DNA primase	-16.84	2.19E-16
DR76_RS00545	hypothetical protein	-16.84	1.47E-09
DR76_RS00550	single-stranded DNA-binding protein	-16.84	2.78E-11
DR76_RS00555	hypothetical protein	-16.84	2.48E-02
DR76_RS00560	hypothetical protein	-16.84	1.65E-12
DR76_RS00565	phage capsid protein	-16.84	6.39E-30
DR76_RS00570	capsid protein small subunit	-16.84	4.38E-20
DR76_RS00575	hypothetical protein	-16.84	4.04E-25
DR76_RS00580	hypothetical protein	-16.84	8.63E-05
DR76_RS00585	terminase	-16.84	9.73E-22
DR76_RS00590	hypothetical protein	-16.84	1.87E-19
DR76_RS00595	ATPase	-5.21	6.90E-07
DR76_RS00635	heme lyase subunit CcmF	-1.25	4.07E-04
DR76_RS00670	nitrate reductase cytochrome C550 subunit	-1.69	1.38E-03
DR76_RS00675	quinol dehydrogenase	-1.41	3.44E-03
DR76_RS00680	ferredoxin-type protein NapG	-1.22	6.06E-03
DR76_RS00705	ecotin	-1.57	1.59E-04
DR76_RS00765	acetoacetate metabolism regulatory protein AtoC	-1.44	1.54E-04
DR76_RS00835	ribonucleotide-diphosphate reductase subunit alpha	-1.87	8.18E-04
DR76_RS00840	ribonucleotide-diphosphate reductase subunit beta	-2.27	4.04E-05
DR76_RS00910	membrane protein	-1.22	2.32E-03
DR76_RS00915	nucleoside triphosphatase NudI	-1.97	2.27E-06
DR76_RS01000	acyltransferase	-3.78	6.12E-16
DR76_RS01005	ribonuclease Z	-1.02	3.06E-03
DR76_RS01010	hypothetical protein	-1.92	1.89E-05

DR76_RS01020	hypothetical protein	-3.49	4.36E-09
DR76_RS01115	hypothetical protein	-1.11	3.37E-03
DR76_RS01260	3-oxoacyl-[acyl-carrier-protein] synthase I	-2.61	5.72E-05
DR76_RS01265	tRNA methyltransferase	-1.35	1.50E-04
DR76_RS01300	peptidase S10	-2.76	5.78E-12
DR76_RS01305	hypothetical protein	-16.84	5.20E-33
DR76_RS01310	pilus protein	-16.84	1.24E-31
DR76_RS01315	fimbrial protein	-16.84	2.54E-14
DR76_RS01320	fimbrial protein SteE	-16.84	1.92E-11
DR76_RS01325	hypothetical protein	-16.84	9.19E-12
DR76_RS01330	fimbrial protein StfD	-16.84	3.77E-19
DR76_RS01335	fimbrial protein SteB	-16.84	1.81E-26
DR76_RS01340	fimbrial yfcV	-16.84	4.26E-17
DR76_RS01365	long-chain fatty acid outer membrane transporter	-2.47	4.77E-10
DR76_RS01385	hypothetical protein	-16.84	5.15E-21
DR76_RS01390	outer membrane autotransporter barrel domain-containing protein	-16.84	2.30E-61
DR76_RS01395	LuxR family transcriptional regulator	-16.84	7.22E-07
DR76_RS01400	pilus protein	-16.84	2.53E-22
DR76_RS01405	DNA recombinase	-16.84	1.55E-17
DR76_RS01410	transcriptional regulator	-16.84	6.05E-24
DR76_RS01415	permease	-1.83	2.23E-04
DR76_RS01420	D-serine dehydratase	-1.00	1.87E-02
DR76_RS01520	aminopeptidase	-1.48	1.09E-03
DR76_RS01570	hypothetical protein	-2.03	9.89E-04
DR76_RS01575	hypothetical protein	-1.08	1.95E-02
DR76_RS01580	DUF1323 family putative DNA-binding protein	-1.15	1.66E-03
DR76_RS01585	hypothetical protein	-3.69	1.99E-16
DR76_RS01595	hypothetical protein	-1.44	3.53E-04
DR76_RS01600	hypothetical protein	-1.87	4.96E-06
DR76_RS01610	hypothetical protein	-2.13	5.71E-03
DR76_RS01630	purine nucleoside phosphorylase	-2.12	6.79E-05
DR76_RS01635	membrane protein	-1.14	1.38E-02
DR76_RS01705	sulfate/thiosulfate import ATP-binding protein CysA	-2.19	4.51E-06
DR76_RS01710	sulfate/thiosulfate ABC transporter permease	-1.28	2.74E-03
DR76_RS01715	sulfate/thiosulfate transporter subunit	-2.08	5.87E-07
DR76_RS01720	thiosulfate transporter subunit	-1.86	4.14E-04
DR76_RS01730	N-acetylmuramic acid-6-phosphate etherase	-4.09	1.15E-20
DR76_RS01735	PTS N-acetylmuramic acid transporter subunit IIBC	-2.93	4.42E-13
DR76_RS01740	deferrochelatase/peroxidase YfeX	-1.19	2.02E-02
DR76_RS01775	carboxysome shell protein	-1.33	3.05E-03
DR76_RS01780	ethanolamine utilization protein EutL	-2.81	2.08E-08
DR76_RS01785	ethanolamine ammonia-lyase small subunit	-16.84	2.10E-20
DR76_RS01790	ethanolamine ammonia lyase large subunit	-16.84	2.49E-23
DR76_RS01795	ethanolamine utilization protein EutA	-16.84	3.64E-19

DR76_RS01800	ethanolamine utilization protein EutH	-16.84	4.85E-23
DR76_RS01805	ethanol dehydrogenase	-6.98	6.53E-14
DR76_RS01810	ethanolamine utilization protein EutJ	-16.84	7.64E-11
DR76_RS01815	aldehyde dehydrogenase	-3.75	4.48E-10
DR76_RS01820	ethanolamine catabolic microcompartment shell protein EutN	-2.51	2.30E-03
DR76_RS01825	ethanolamine utilization protein-2C putative carboxysome structural protein	-1.89	1.06E-05
DR76_RS01830	phosphotransacetylase	-1.80	9.57E-05
DR76_RS01840	ethanolamine utilization protein EutQ	-3.09	2.16E-10
DR76_RS01845	ethanolamine utilization protein EutP	-1.35	5.40E-03
DR76_RS01850	carboxysome shell protein	-1.42	9.21E-03
DR76_RS01880	oxidoreductase Fe-S binding subunit	-1.17	1.39E-03
DR76_RS01895	hypothetical protein	-1.30	1.71E-03
DR76_RS01900	reductase	-1.69	3.30E-06
DR76_RS01905	succinyl-diaminopimelate desuccinylase	-1.32	3.28E-04
DR76_RS02040	inosine 5'-monophosphate dehydrogenase	-1.29	2.28E-03
DR76_RS02050	hypothetical protein	-12.31	1.30E-57
DR76_RS02055	hypothetical protein	-16.84	1.14E-38
DR76_RS02060	intimin	-16.84	9.37E-25
DR76_RS02065	hypothetical protein	-16.84	1.17E-15
DR76_RS02120	3-mercaptopyruvate sulfurtransferase	-1.95	8.04E-05
DR76_RS02225	sugar ABC transporter substrate-binding protein	-2.36	2.51E-06
DR76_RS02245	bifunctional nitric oxide dioxygenase/dihydropteridine reductase 2	-2.28	2.68E-04
DR76_RS02300	Small toxic protein shoB	-2.42	6.13E-03
DR76_RS02305	hypothetical protein	-3.62	9.66E-11
DR76_RS02395	autonomous glycyl radical cofactor GrcA	-1.65	1.01E-02
DR76_RS02400	uracil-DNA glycosylase	-1.05	1.91E-03
DR76_RS02450	hypothetical protein	-16.84	2.50E-02
DR76_RS02460	hypothetical protein	-16.84	3.08E-03
DR76_RS02470	vitamin B12/cobalamin outer membrane transporter	-1.98	1.99E-04
DR76_RS02490	MFS transporter	-4.23	1.14E-12
DR76_RS02495	peptidase M20	-16.84	6.15E-21
DR76_RS02515	bifunctional D-altronate/D-mannonate dehydratase	-1.58	2.60E-03
DR76_RS02570	PTS fructose transporter subunit IIB	-1.73	9.49E-04
DR76_RS02575	PTS fructose transporter subunit IIC	-1.63	2.49E-03
DR76_RS02580	PTS fructose transporter subunit IIA	-1.01	6.60E-03
DR76_RS02585	fructose-bisphosphate aldolase	-6.35	3.63E-29
DR76_RS02590	glycerol dehydrogenase-2C NAD+ dependent-3B 1-2C2-propanediol:NAD+ oxidoreductase	-3.24	5.42E-09
DR76_RS02595	hypothetical protein	-2.13	1.38E-05
DR76_RS02610	catalase/hydroperoxidase HPI(I)	-1.50	9.64E-03
DR76_RS02615	5-2C10-methylenetetrahydrofolate reductase	-3.32	4.98E-13
DR76_RS02620	5'-nucleotidase	-16.84	2.02E-26
DR76_RS02625	hypothetical protein	-16.84	6.58E-23
DR76_RS02630	5'-nucleotidase	-16.84	2.70E-23

DR76_RS02635	5'-nucleotidase	-16.84	9.23E-21
DR76_RS02640	ion channel protein Tsx	-16.84	6.31E-12
DR76_RS02715	glycerol kinase	-2.07	4.60E-07
DR76_RS02720	fructose 1-2C6-bisphosphatase II	-1.66	6.24E-05
DR76_RS02760	sulfate transporter subunit	-2.08	1.09E-04
DR76_RS02855	glycoporin	-16.84	2.51E-10
DR76_RS02895	hypothetical protein	-2.84	1.43E-03
DR76_RS02900	hypothetical protein	-16.84	2.70E-54
DR76_RS02905	hypothetical protein	-16.84	7.44E-53
DR76_RS02910	Cro/Cl family transcriptional regulator	-16.84	1.10E-54
DR76_RS02915	hypothetical protein	-16.84	1.49E-41
DR76_RS02920	lipase	-16.84	6.23E-58
DR76_RS02945	aldose epimerase	-16.84	4.19E-24
DR76_RS02950	MFS transporter	-16.84	1.36E-14
DR76_RS02955	MFS transporter	-16.84	7.59E-22
DR76_RS02960	molecular chaperone GroES	-16.84	7.71E-18
DR76_RS02965	aldolase	-16.84	6.56E-15
DR76_RS02970	NADH-dependent gamma-hydroxybutyrate dehydrogenase	-16.84	3.10E-15
DR76_RS02975	sugar kinase	-16.84	6.75E-33
DR76_RS02980	DeoR family transcriptional regulator	-16.84	1.26E-42
DR76_RS02985	membrane protein	-2.10	5.70E-07
DR76_RS03025	uncharacterized protein	-1.38	5.93E-04
DR76_RS03190	DNA recombination protein RmuC	-2.26	2.59E-08
DR76_RS03195	transcriptional regulator	-16.84	3.00E-27
DR76_RS03200	acidic carbohydrate kinase	-16.84	1.06E-26
DR76_RS03205	2-dehydro-3-deoxy-6-phosphogalactonate aldolase	-16.84	4.75E-21
DR76_RS03210	ABC transporter substrate-binding protein	-16.84	9.81E-27
DR76_RS03215	permease	-16.84	4.25E-18
DR76_RS03220	C4-dicarboxylate ABC transporter permease	-16.84	2.48E-41
DR76_RS03235	hypothetical protein	-16.84	1.12E-04
DR76_RS03240	cysteine hydrolase	-16.84	3.74E-26
DR76_RS03245	hypothetical protein	-16.84	4.07E-21
DR76_RS03250	FdrA	-16.84	5.72E-17
DR76_RS03255	hypothetical protein	-16.84	5.93E-17
DR76_RS03260	carbamate kinase	-16.84	1.21E-13
DR76_RS03265	glutamyl-tRNA amidotransferase	-16.84	1.94E-09
DR76_RS03270	permease	-8.36	1.32E-22
DR76_RS03275	LysR family transcriptional regulator	-16.84	1.13E-29
DR76_RS03280	sugar isomerase	-16.84	4.15E-38
DR76_RS03285	transketolase	-16.84	4.86E-35
DR76_RS03290	PTS sugar transporter	-16.84	9.59E-39
DR76_RS03295	6-phospho 3-hexuloisomerase	-16.84	7.07E-27
DR76_RS03300	hypothetical protein	-16.84	1.74E-08
DR76_RS03305	HNH endonuclease	-16.84	9.80E-14

DR76_RS03310	hypothetical protein	-16.84	1.89E-19
DR76_RS03315	hypothetical protein	-16.84	1.43E-22
DR76_RS03375	hypothetical protein	-16.84	9.47E-07
DR76_RS03400	acetolactate synthase	-16.84	2.40E-57
DR76_RS03435	hypothetical protein	-6.82	2.54E-22
DR76_RS03440	hypothetical protein	-5.66	2.18E-15
DR76_RS03475	arylsulfatase	-2.63	2.80E-08
DR76_RS03480	arylsulfatase	-2.04	1.17E-06
DR76_RS03610	ketol-acid reductoisomerase	-1.20	2.47E-02
DR76_RS03615	transcriptional regulator	-2.74	4.23E-12
DR76_RS03620	threonine dehydratase	-1.24	2.74E-04
DR76_RS03825	glucosamine--fructose-6-phosphate aminotransferase	-1.41	4.86E-03
DR76_RS03855	transcription antitermination protein BlgG	-1.84	2.23E-03
DR76_RS03885	inner membrane protein CbrB	-2.21	9.55E-06
DR76_RS03890	6-phosphogluconate phosphatase	-2.00	1.27E-06
DR76_RS03915	multidrug resistance protein MdtL	-1.17	2.28E-02
DR76_RS03920	tryptophan permease	-5.55	2.13E-06
DR76_RS03925	L-cysteine desulfhydrase	-6.15	1.97E-07
DR76_RS03930	tryptophanase leader peptide	-5.03	4.92E-06
DR76_RS03995	hypothetical protein	-16.84	1.69E-19
DR76_RS04035	hypothetical protein	-1.02	3.83E-03
DR76_RS04055	transporter	-2.22	2.34E-09
DR76_RS04110	membrane protein	-7.30	1.07E-16
DR76_RS04115	hypothetical protein	-16.84	5.03E-03
DR76_RS04130	regulatory protein	-1.15	3.17E-03
DR76_RS04135	hexose phosphate transporter	-3.15	7.67E-08
DR76_RS04140	adenine deaminase	-2.11	4.70E-04
DR76_RS04170	methionine ABC transporter substrate-binding protein	-1.52	4.30E-05
DR76_RS04180	hypothetical protein	-16.84	2.30E-05
DR76_RS04185	hypothetical protein	-16.84	7.40E-10
DR76_RS04190	hypothetical protein	-16.84	3.12E-15
DR76_RS04195	hypothetical protein	-16.84	4.45E-05
DR76_RS04200	hypothetical protein	-16.84	7.92E-41
DR76_RS04205	ATP-binding protein	-16.84	8.19E-62
DR76_RS04210	hypothetical protein	-16.84	9.19E-11
DR76_RS04215	hypothetical protein	-16.84	8.55E-09
DR76_RS04220	hypothetical protein	-16.84	2.91E-09
DR76_RS04225	hypothetical protein	-16.84	5.06E-08
DR76_RS04230	hypothetical protein	-16.84	3.30E-23
DR76_RS04235	malate transporter	-16.84	2.01E-13
DR76_RS04240	Rha family transcriptional regulator	-16.84	1.31E-06
DR76_RS04245	hypothetical protein	-16.84	4.86E-14
DR76_RS04250	GTPase	-16.84	2.60E-16
DR76_RS04255	transcriptional regulator	-16.84	4.24E-16

DR76_RS04260	hypothetical protein	-16.84	2.41E-59
DR76_RS04265	threonine transporter	-16.84	1.01E-43
DR76_RS04270	hypothetical protein	-16.84	2.17E-50
DR76_RS04275	hypothetical protein	-16.84	3.79E-22
DR76_RS04280	hypothetical protein	-16.84	5.47E-47
DR76_RS04285	hypothetical protein	-16.84	4.34E-29
DR76_RS04290	integrase	-16.84	1.07E-37
DR76_RS04295	hypothetical protein	-4.06	1.44E-10
DR76_RS04310	transcriptional antiterminator	-16.84	1.80E-17
DR76_RS04315	putative phosphotransferase system (PTS)-2Cfructose-specific	-16.84	4.92E-08
DR76_RS04320	PTS fructose transporter subunit IIB	-16.84	3.50E-08
DR76_RS04325	FruA	-16.84	4.54E-12
DR76_RS04330	aldolase	-16.84	1.92E-12
DR76_RS04335	hypothetical protein	-16.84	7.82E-12
DR76_RS04340	fructokinase	-16.84	9.43E-12
DR76_RS04345	D-lyxose isomerase	-16.84	7.06E-24
DR76_RS04400	DNA damage-inducible protein D	-5.08	1.59E-17
DR76_RS04525	2-amino-3-ketobutyrate CoA ligase	-1.43	3.40E-03
DR76_RS04530	L-threonine 3-dehydrogenase	-1.72	2.99E-04
DR76_RS04600	adhesin	-2.53	2.34E-11
DR76_RS04610	ribosome-associated DUF2810 family protein	-1.72	5.54E-06
DR76_RS04665	hypothetical protein	-16.84	9.38E-30
DR76_RS04675	L-ribulose-5-phosphate 4-epimerase	-4.08	1.26E-07
DR76_RS04680	L-xylulose 5-phosphate 3-epimerase	-2.43	1.34E-03
DR76_RS04685	3-keto-L-gulonate-6-phosphate decarboxylase	-2.31	1.13E-03
DR76_RS04690	L-xylulose/3-keto-L-gulonate kinase	-1.89	1.40E-03
DR76_RS04695	C4-dicarboxylate ABC transporter substrate-binding protein	-3.40	3.42E-05
DR76_RS04700	dehydroascorbate transporter	-16.84	3.43E-11
DR76_RS04705	2-2C3-diketo-L-gulonate TRAP transporter permease	-16.84	2.95E-06
DR76_RS04710	hypothetical protein	-16.84	3.45E-17
DR76_RS04715	hypothetical protein	-2.73	1.03E-05
DR76_RS04720	2-2C3-diketo-L-gulonate reductase	-1.25	3.10E-03
DR76_RS04770	xylose ABC transporter substrate-binding protein	-1.69	4.04E-05
DR76_RS04835	bifunctional glyoxylate/hydroxypyruvate reductase B	-2.22	1.60E-05
DR76_RS04865	membrane protein	-1.41	3.80E-04
DR76_RS04925	hypothetical protein	-2.44	1.66E-09
DR76_RS05005	cyclic di-GMP phosphodiesterase	-4.71	1.92E-15
DR76_RS05100	hemin ABC transporter ATP-binding protein	-16.84	2.47E-19
DR76_RS05105	putative permease of iron compound ABC transport system	-16.84	9.33E-23
DR76_RS05110	hypothetical protein	-16.84	4.32E-22
DR76_RS05115	ShuX	-16.84	4.39E-20
DR76_RS05120	coproporphyrinogen III oxidase	-16.84	1.57E-20
DR76_RS05125	ABC transporter substrate-binding protein	-16.84	1.67E-16
DR76_RS05130	hypothetical protein	-16.84	5.45E-08

DR76_RS05135	ligand-gated channel protein	-16.84	3.95E-26
DR76_RS05140	hemin transporter	-16.84	2.90E-24
DR76_RS05155	arsenate reductase	-3.08	3.32E-11
DR76_RS05160	hypothetical protein	-3.77	1.76E-09
DR76_RS05215	membrane protein	-16.84	4.57E-17
DR76_RS05220	membrane protein	-1.74	2.13E-05
DR76_RS05225	multidrug ABC transporter ATP-binding protein	-1.82	3.43E-04
DR76_RS05235	fructose-bisphosphate aldolase	-16.84	8.51E-29
DR76_RS05240	phosphocarrier protein HPr	-16.84	1.93E-05
DR76_RS05245	carbohydrate kinase	-16.84	1.89E-23
DR76_RS05250	PTS galactitol transporter subunit IIC	-16.84	1.56E-24
DR76_RS05255	PTS sugar transporter subunit IIB	-16.84	7.24E-21
DR76_RS05260	PTS suar transporter subunit IIA	-16.84	3.75E-24
DR76_RS05265	regulatory protein	-16.84	6.23E-27
DR76_RS05270	nickel responsive regulator	-1.91	3.73E-05
DR76_RS05275	nickel ABC transporter ATP-binding protein	-1.36	3.93E-03
DR76_RS05280	nickel ABC transporter ATP-binding protein	-2.51	1.65E-04
DR76_RS05285	nickel ABC transporter permease	-2.59	7.43E-06
DR76_RS05295	nickel ABC transporter substrate-binding protein	-2.28	2.18E-06
DR76_RS05325	sulfurtransferase TusA	-1.46	3.07E-04
DR76_RS05380	hypothetical protein	-16.84	2.14E-14
DR76_RS05385	hypothetical protein	-1.06	3.73E-03
DR76_RS05460	hypothetical protein	-16.84	7.46E-05
DR76_RS05465	hypothetical protein	-16.84	2.43E-07
DR76_RS05505	ATP-dependent DNA helicase RecQ	-16.84	7.76E-60
DR76_RS05510	DNA processing protein DprA	-16.84	3.17E-54
DR76_RS05550	hypothetical protein	-16.84	6.80E-52
DR76_RS05555	fimbrial protein	-16.84	3.25E-14
DR76_RS05560	outer membrane usher protein	-16.84	2.46E-22
DR76_RS05565	fimbrial protein	-16.84	1.01E-05
DR76_RS05570	auf fimbriae minor subunit AufE	-16.84	2.74E-04
DR76_RS05575	pilin chaperone	-16.84	4.25E-10
DR76_RS05580	fimbrial protein	-16.84	1.13E-08
DR76_RS05640	glycogen phosphorylase	-2.04	6.62E-05
DR76_RS05645	4-alpha-glucanotransferase	-1.45	8.08E-04
DR76_RS05675	iron transporter	-1.78	7.27E-06
DR76_RS05710	XRE family transcriptional regulator	-16.84	2.81E-29
DR76_RS05715	hypothetical protein	-16.84	4.20E-33
DR76_RS05720	phosphoenolpyruvate carboxykinase	-1.49	3.48E-03
DR76_RS05730	membrane protein	-1.04	2.61E-03
DR76_RS05825	hypothetical protein	-2.05	7.63E-07
DR76_RS05835	hypothetical protein	-1.31	1.37E-02
DR76_RS05860	hypothetical protein	-1.54	4.80E-04
DR76_RS06125	50S ribosomal protein L4	-1.11	2.30E-02

DR76_RS06130	50S ribosomal protein L23	-1.18	1.33E-02
DR76_RS06140	30S ribosomal protein S19	-1.05	1.77E-02
DR76_RS06145	50S ribosomal protein L22	-1.11	1.77E-02
DR76_RS06160	50S ribosomal protein L29	-1.06	6.13E-03
DR76_RS06165	30S ribosomal protein S17	-1.00	2.06E-02
DR76_RS06395	transcriptional regulator	-3.11	1.52E-03
DR76_RS06400	putative membrane protein	-2.48	2.33E-02
DR76_RS06435	sugar kinase	-16.84	1.33E-41
DR76_RS06440	DeoR family transcriptional regulator	-9.53	2.22E-30
DR76_RS06445	fructose-bisphosphate aldolase	-16.84	9.59E-13
DR76_RS06450	sugar ABC transporter	-16.84	3.13E-16
DR76_RS06455	D-ribose transporter ATP binding protein	-16.84	3.89E-13
DR76_RS06460	sugar ABC transporter permease	-16.84	3.99E-08
DR76_RS06465	sugar kinase	-16.84	9.74E-12
DR76_RS06470	hypothetical protein	-16.84	7.65E-27
DR76_RS06490	hypothetical protein	-3.74	2.14E-09
DR76_RS06495	hypothetical protein	-1.25	6.49E-03
DR76_RS06500	quinone oxidoreductase	-1.06	1.18E-02
DR76_RS06635	N-acetylneuraminate lyase	-2.10	2.65E-04
DR76_RS06670	hypothetical protein	-1.44	1.37E-04
DR76_RS06925	tryptophan permease	-2.73	6.18E-04
DR76_RS06935	protease	-1.40	1.61E-03
DR76_RS06940	protease	-1.42	6.28E-04
DR76_RS07000	galactosamine-6-phosphate isomerase	-2.45	8.31E-09
DR76_RS07005	PTS N-acetylgalactosamine transporter subunit IID	-1.96	2.21E-04
DR76_RS07010	PTS N-acetylgalactosamine transporter subunit IIC	-2.30	1.18E-04
DR76_RS07015	PTS N-acetylgalactosamine transporter subunit IIB	-3.47	3.58E-06
DR76_RS07020	D-tagatose-1-2C6-bisphosphate aldolase subunit KbaY	-3.15	3.84E-09
DR76_RS07025	aldose isomerase	-2.71	2.23E-08
DR76_RS07030	N-acetylglucosamine-6-phosphate deacetylase	-1.67	7.35E-05
DR76_RS07035	PTS N-acetylgalactosamine transporter subunit IIA	-2.02	1.54E-05
DR76_RS07040	PTS N-acetylgalactosamine transporter subunit IID	-2.80	3.73E-10
DR76_RS07045	PTS N-acetylgalactosamine transporter subunit IIC	-4.30	7.60E-16
DR76_RS07050	PTS N-acetylgalactosamine transporter subunit IIB	-3.65	1.46E-12
DR76_RS07055	tagatose-bisphosphate aldolase	-2.56	1.35E-09
DR76_RS07060	DeoR family transcriptional regulator	-1.39	9.31E-05
DR76_RS07065	toxin YhaV	-1.16	2.03E-03
DR76_RS07070	regulator	-1.12	3.22E-03
DR76_RS07075	galactarate dehydrogenase	-3.64	1.72E-15
DR76_RS07080	galactonate transporter	-2.79	6.40E-13
DR76_RS07085	alpha-dehydro-beta-deoxy-D-glucarate aldolase	-2.49	9.00E-11
DR76_RS07090	tartronate semialdehyde reductase	-2.06	1.95E-08
DR76_RS07095	glycerate kinase	-1.32	1.98E-04
DR76_RS07105	transcriptional regulator	-4.66	6.27E-10

DR76_RS07110	threonine dehydratase	-6.39	3.36E-09
DR76_RS07115	threonine/serine transporter TdcC	-6.43	2.23E-08
DR76_RS07120	propionate/acetate kinase	-7.08	1.40E-08
DR76_RS07125	keto-acid formate acetyltransferase	-8.56	1.89E-09
DR76_RS07130	putative reactive intermediate deaminase	-7.52	5.28E-11
DR76_RS07135	L-serine dehydratase	-6.82	2.20E-08
DR76_RS07140	membrane protein	-1.60	2.10E-04
DR76_RS07145	membrane protein	-1.88	1.03E-05
DR76_RS07215	hexuronate transporter ExuT	-1.41	4.73E-04
DR76_RS07220	glucuronate isomerase	-2.73	1.05E-08
DR76_RS07225	altronate hydrolase	-2.44	6.15E-08
DR76_RS07245	oxidoreductase	-2.10	9.43E-07
DR76_RS07250	hypothetical protein	-1.03	1.01E-02
DR76_RS07265	toxin RelE	-6.71	2.60E-13
DR76_RS07270	transcriptional regulator	-16.84	4.70E-32
DR76_RS07285	hypothetical protein	-1.19	6.26E-03
DR76_RS07290	amino acid permease	-1.15	1.42E-02
DR76_RS07295	beta-D-galactosidase subunit beta	-2.23	1.56E-05
DR76_RS07320	aerotaxis receptor	-3.11	4.95E-10
DR76_RS07325	transcriptional regulator	-1.02	5.32E-03
DR76_RS07370	L(+)-tartrate dehydratase subunit beta	-3.01	3.22E-11
DR76_RS07375	tartrate dehydratase subunit alpha	-1.34	3.65E-03
DR76_RS07380	LysR family transcriptional regulator	-1.98	3.72E-07
DR76_RS07415	transposase	-16.84	4.51E-56
DR76_RS07420	transposase	-16.84	6.91E-28
DR76_RS07445	membrane protein	-1.94	3.28E-06
DR76_RS07460	hypothetical protein	-1.97	1.64E-07
DR76_RS07470	pilus protein	-16.84	1.26E-22
DR76_RS07475	fimbrial assembly protein PapD	-16.84	6.37E-24
DR76_RS07480	fimbrial outer membrane usher protein StdB	-16.84	1.11E-31
DR76_RS07485	fimbrial protein	-16.84	2.48E-10
DR76_RS07490	hypothetical protein	-16.84	5.02E-31
DR76_RS07505	disulfide oxidoreductase	-16.84	5.72E-31
DR76_RS07510	thiol:disulfide interchange protein DsbL	-16.84	7.95E-19
DR76_RS07515	arylsulfate sulfotransferase	-16.84	2.51E-21
DR76_RS07565	TonB-dependent receptor	-16.84	1.95E-27
DR76_RS07570	ABC transporter	-16.84	9.56E-22
DR76_RS07575	heme ABC transporter	-16.84	9.27E-19
DR76_RS07580	transporter	-16.84	1.46E-20
DR76_RS07585	preprotein translocase subunit YidC	-16.84	1.35E-26
DR76_RS07590	RpiR family transcriptional regulator	-16.84	3.74E-28
DR76_RS07660	membrane protein	-16.84	2.12E-21
DR76_RS07665	C4-dicarboxylate ABC transporter permease	-16.84	1.73E-03
DR76_RS07670	C4-dicarboxylate ABC transporter substrate-binding protein	-16.84	5.22E-11

DR76_RS07675	ureidoglycolate dehydrogenase	-16.84	6.82E-14
DR76_RS07680	galactonate oxidoreductase	-16.84	2.39E-14
DR76_RS07685	fructuronate reductase	-16.84	1.38E-22
DR76_RS07690	transcriptional regulator	-16.84	5.55E-36
DR76_RS07765	L-glyceraldehyde 3-phosphate reductase	-2.53	4.16E-09
DR76_RS07790	hydrogenase	-1.17	1.28E-02
DR76_RS07795	hydrogenase	-1.57	1.29E-03
DR76_RS07800	hydrogenase 2 large subunit	-1.13	2.19E-02
DR76_RS07805	hydrogenase 2 maturation endopeptidase	-1.24	2.77E-03
DR76_RS07810	hydrogenase	-1.23	2.40E-03
DR76_RS07820	hydrogenase 2 accessory protein HypG	-1.25	3.65E-04
DR76_RS07940	hypothetical protein	-16.84	1.85E-26
DR76_RS07945	transposase	-16.84	1.96E-03
DR76_RS07955	hypothetical protein	-1.21	5.84E-03
DR76_RS07960	general secretion pathway protein	-1.34	1.53E-02
DR76_RS07965	polysialic acid transporter	-16.84	4.21E-43
DR76_RS07970	ABC transporter ATP-binding protein	-16.84	2.73E-35
DR76_RS07975	hypothetical protein	-16.84	3.64E-24
DR76_RS07980	hypothetical protein	-16.84	1.20E-27
DR76_RS07985	glycosyl transferase family 1	-14.68	5.29E-25
DR76_RS07990	hypothetical protein	-16.84	5.13E-25
DR76_RS07995	hypothetical protein	-16.84	1.66E-26
DR76_RS08000	capsular polysaccharide biosynthesis protein	-16.84	1.52E-34
DR76_RS08005	capsule polysaccharide transporter	-12.24	6.26E-55
DR76_RS08010	3-deoxy-manno-octulosonate cytidylyltransferase	-16.84	8.73E-56
DR76_RS08015	polysialic acid transporter	-16.84	6.82E-62
DR76_RS08020	transporter	-16.84	8.99E-58
DR76_RS08025	arabinose 5-phosphate isomerase	-13.08	1.68E-61
DR76_RS08030	transposase	-16.84	4.14E-29
DR76_RS08035	hypothetical protein	-16.84	3.78E-48
DR76_RS08040	S-adenosylhomocysteine hydrolase	-16.84	1.72E-50
DR76_RS08045	restriction methylase	-4.03	5.51E-16
DR76_RS08050	transposase	-16.84	4.88E-24
DR76_RS08055	hypothetical protein	-1.49	9.03E-04
DR76_RS08060	hypothetical protein	-16.84	9.30E-20
DR76_RS08070	antitoxin	-1.19	8.67E-03
DR76_RS08075	hypothetical protein	-9.62	2.09E-31
DR76_RS08080	hypothetical protein	-16.84	3.40E-06
DR76_RS08085	hypothetical protein	-3.33	1.31E-05
DR76_RS08090	hypothetical protein	-3.28	1.26E-07
DR76_RS08100	hypothetical protein	-16.84	1.84E-14
DR76_RS08105	hypothetical protein	-16.84	1.10E-03
DR76_RS08115	hypothetical protein	-16.84	1.69E-19
DR76_RS08120	hypothetical protein	-16.84	5.07E-53

DR76_RS08125	isocitrate lyase	-16.84	4.33E-10
DR76_RS08130	transposase	-16.84	4.69E-19
DR76_RS08135	transposase	-16.84	2.54E-60
DR76_RS08140	cytochrome O ubiquinol oxidase	-16.84	1.44E-13
DR76_RS08145	hypothetical protein	-16.84	1.35E-19
DR76_RS08150	hypothetical protein	-8.68	2.81E-32
DR76_RS08155	hypothetical protein	-16.84	5.67E-07
DR76_RS08160	hypothetical protein	-16.84	1.20E-07
DR76_RS08165	hypothetical protein	-10.03	7.91E-46
DR76_RS08170	hypothetical protein	-16.84	2.38E-48
DR76_RS08175	Rha family transcriptional regulator	-16.84	3.60E-26
DR76_RS08180	hypothetical protein	-16.84	1.63E-29
DR76_RS08185	hypothetical protein	-16.84	3.03E-28
DR76_RS08190	hypothetical protein	-4.49	3.59E-05
DR76_RS08195	hypothetical protein	-16.84	8.70E-03
DR76_RS08200	hypothetical protein	-16.84	8.54E-03
DR76_RS08205	transposase	-4.78	1.16E-05
DR76_RS08210	transposase	-16.84	1.80E-40
DR76_RS08215	isocitrate lyase	-16.84	1.20E-09
DR76_RS08220	transposase	-16.84	7.72E-11
DR76_RS08225	hypothetical protein	-16.84	1.72E-04
DR76_RS08230	hypothetical protein	-16.84	4.57E-11
DR76_RS08235	N-acetylneuraminate lyase	-16.84	1.00E-18
DR76_RS08240	N-acetylmannosamine kinase	-16.84	1.49E-15
DR76_RS08245	sialic acid transporter	-16.84	3.17E-17
DR76_RS08250	peptidase S9	-16.84	3.24E-12
DR76_RS08255	N-acetylneuraminic acid channel protein	-16.84	1.30E-09
DR76_RS08260	N-acetylneuraminic acid mutarotase	-16.84	2.25E-21
DR76_RS08265	hydrolase	-12.57	8.00E-53
DR76_RS08270	transcriptional regulator	-16.84	5.85E-54
DR76_RS08275	hypothetical protein	-16.84	2.02E-43
DR76_RS08280	MFS transporter	-16.84	1.45E-42
DR76_RS08285	aerobactin synthase IucA	-16.84	1.02E-32
DR76_RS08290	N(6)-hydroxylysine O-acetyltransferase	-16.84	1.10E-27
DR76_RS08295	aerobactin synthase IucC	-16.84	1.76E-41
DR76_RS08300	lysine 6-monooxygenase	-16.84	1.43E-52
DR76_RS08305	ligand-gated channel protein	-16.84	4.59E-50
DR76_RS08310	hypothetical protein	-16.84	1.78E-14
DR76_RS08315	transporter	-16.84	6.64E-61
DR76_RS08320	hypothetical protein	-16.84	2.79E-31
DR76_RS08325	transposase	-16.84	3.60E-17
DR76_RS08330	isocitrate lyase	-16.84	5.32E-19
DR76_RS08335	transposase	-16.84	1.11E-41
DR76_RS08340	transposase	-16.84	1.15E-03

DR76_RS08345	transposase	-16.84	2.26E-18
DR76_RS08370	ligand-gated channel	-16.84	4.18E-42
DR76_RS08375	hypothetical protein	-16.84	3.15E-32
DR76_RS08380	hypothetical protein	-16.84	6.45E-51
DR76_RS08385	hypothetical protein	-16.84	1.45E-29
DR76_RS08390	hypothetical protein	-16.84	4.81E-30
DR76_RS08395	hypothetical protein	-16.84	7.32E-21
DR76_RS08400	hypothetical protein	-16.84	3.18E-23
DR76_RS08405	membrane protein	-16.84	8.18E-34
DR76_RS08410	TetR family transcriptional regulator	-16.84	2.11E-40
DR76_RS08415	hypothetical protein	-16.84	4.69E-25
DR76_RS08420	hypothetical protein	-16.84	2.72E-18
DR76_RS08425	hypothetical protein	-16.84	8.50E-26
DR76_RS08430	hypothetical protein	-16.84	6.44E-18
DR76_RS08435	hypothetical protein	-16.84	5.29E-26
DR76_RS08440	transposase	-16.84	7.22E-05
DR76_RS08460	transposase	-16.84	7.48E-15
DR76_RS08465	Major pilus subunit operon regulatory protein	-16.84	3.15E-18
DR76_RS08475	Major pilu subunit operon regulatory protein papB	-16.84	6.18E-34
DR76_RS08480	F7-2 fimbrial protein	-14.50	9.38E-28
DR76_RS08485	PAP fimbrial minor pilin protein	-16.84	1.68E-35
DR76_RS08490	hypothetical protein	-16.84	2.66E-55
DR76_RS08495	molecular chaperone	-16.84	2.61E-31
DR76_RS08500	protein papJ	-16.84	2.98E-22
DR76_RS08505	fimbrial adapter papK	-16.84	1.59E-24
DR76_RS08510	fimbrial protein	-16.84	7.89E-23
DR76_RS08515	fimbrial adapter papF	-16.84	2.43E-22
DR76_RS08520	fimbrial protein	-16.84	5.68E-31
DR76_RS08525	transcriptional regulator	-16.84	6.23E-47
DR76_RS08530	protein encoded within IS	-16.84	1.98E-31
DR76_RS08535	transposase	-16.84	1.71E-29
DR76_RS08540	hypothetical protein-2C putative transposase ORF2-2CIS66 family	-16.84	8.80E-16
DR76_RS08545	transposase	-16.84	2.67E-11
DR76_RS08550	transposase	-16.84	7.83E-20
DR76_RS08555	transposase	-16.84	9.56E-38
DR76_RS08560	hemolysin D	-16.84	8.72E-29
DR76_RS08565	peptidase C39	-16.84	7.85E-25
DR76_RS08570	exotoxin paxA	-16.84	1.10E-39
DR76_RS08575	hemolysin-activating lysine-acyltransferase hlyC	-16.84	1.39E-28
DR76_RS08580	hypothetical protein	-16.84	8.76E-32
DR76_RS08585	hypothetical protein	-16.84	1.44E-31
DR76_RS08590	membrane protein	-16.84	5.21E-22
DR76_RS08595	PhoB family transcriptional regulator	-16.84	5.03E-51
DR76_RS08600	sensor histidine kinase	-16.84	9.86E-52

DR76_RS08605	transposase	-16.84	1.23E-21
DR76_RS08610	transposase	-16.84	4.59E-25
DR76_RS08615	hypothetical protein	-16.84	3.08E-12
DR76_RS08620	transposase	-16.84	5.23E-12
DR76_RS08625	phosphoethanolamine transferase	-16.84	8.35E-30
DR76_RS08630	hypothetical protein	-16.84	9.86E-48
DR76_RS08635	hypothetical protein	-16.84	2.07E-36
DR76_RS08645	membrane protein	-1.16	4.83E-03
DR76_RS08655	nucleoside permease	-2.63	6.47E-08
DR76_RS08695	L-asparaginase	-3.18	1.22E-07
DR76_RS08700	hypothetical protein	-2.54	2.70E-07
DR76_RS08810	transketolase	-2.61	3.31E-05
DR76_RS08815	SPFH domain protein	-16.84	1.67E-29
DR76_RS08855	cobalt ABC transporter ATP-binding protein	-1.53	4.53E-04
DR76_RS08860	cobalt ABC transporter permease	-2.25	5.99E-07
DR76_RS08865	hypothetical protein	-1.87	2.19E-05
DR76_RS08870	DNA-binding protein	-4.41	2.42E-16
DR76_RS08910	methylmalonyl-CoA mutase	-1.72	3.65E-04
DR76_RS08970	glycine cleavage system protein H	-1.51	4.41E-03
DR76_RS08980	6-phospho-beta-glucosidase	-1.63	3.79E-04
DR76_RS08990	hemolysin	-1.21	3.96E-03
DR76_RS09100	selenate reductase	-2.66	2.49E-02
DR76_RS09110	hypothetical protein	-3.61	1.54E-05
DR76_RS09115	hypothetical protein	-3.75	3.55E-04
DR76_RS09145	Fis family transcriptional regulator	-2.58	2.10E-04
DR76_RS09150	xanthine dehydrogenase subunit C	-2.76	4.54E-06
DR76_RS09155	xanthine dehydrogenase subunit B	-1.92	7.02E-03
DR76_RS09170	-	-1.95	1.01E-04
DR76_RS09255	hypothetical protein	-1.37	2.11E-03
DR76_RS09280	hypothetical protein	-1.83	2.57E-05
DR76_RS09290	hypothetical protein	-1.89	1.04E-05
DR76_RS09330	antitermination protein	-16.84	6.89E-04
DR76_RS09335	PTS glucose transporter subunit IIBC	-16.84	3.41E-10
DR76_RS09340	aminotransferase	-16.84	9.72E-07
DR76_RS09345	isomerase	-16.84	5.50E-09
DR76_RS09350	phosphoglycerate dehydrogenase	-16.84	1.55E-13
DR76_RS09355	hypothetical protein	-16.84	2.76E-26
DR76_RS09360	hypothetical protein	-16.84	3.78E-15
DR76_RS09365	type VI secretion protein	-16.84	7.30E-15
DR76_RS09370	hypothetical protein	-16.84	8.76E-26
DR76_RS09375	type VI secretion protein ImpG	-16.84	1.25E-30
DR76_RS09380	hypothetical protein	-16.84	7.61E-24
DR76_RS09385	type VI secretion protein VasK	-16.84	7.69E-34
DR76_RS09390	hypothetical protein	-16.84	5.73E-25

DR76_RS09395	membrane protein	-16.84	2.00E-11
DR76_RS09400	transposase	-16.84	5.56E-05
DR76_RS09405	hypothetical protein	-16.84	7.17E-22
DR76_RS09410	hypothetical protein	-16.84	1.27E-14
DR76_RS09415	hypothetical protein	-16.84	2.54E-38
DR76_RS09420	hypothetical protein	-16.84	2.62E-27
DR76_RS09425	hypothetical protein	-16.84	2.07E-17
DR76_RS09430	ATPase	-16.84	8.31E-20
DR76_RS09435	Secreted protein Hcp	-16.84	1.07E-16
DR76_RS09440	hypothetical protein	-16.84	2.80E-12
DR76_RS09445	type VI secretion protein ImpK	-16.84	5.49E-06
DR76_RS09450	hypothetical protein	-16.84	3.15E-16
DR76_RS09455	hypothetical protein	-16.84	1.85E-19
DR76_RS09460	hypothetical protein	-16.84	1.64E-06
DR76_RS09465	membrane protein	-3.53	6.49E-06
DR76_RS09485	sulfur acceptor protein CsdL	-1.24	3.55E-04
DR76_RS09525	L-fucose mutarotase	-1.79	2.30E-06
DR76_RS09530	L-fuculokinase	-1.88	2.84E-06
DR76_RS09535	fucose isomerase	-2.37	2.40E-07
DR76_RS09540	L-fucose transporter	-2.12	5.09E-08
DR76_RS09545	fuculose phosphate aldolase	-1.94	8.88E-07
DR76_RS09550	L-1-2C2-propanediol oxidoreductase	-1.67	1.31E-04
DR76_RS09555	endonuclease	-1.35	1.32E-04
DR76_RS09560	L-serine dehydratase	-1.06	1.82E-03
DR76_RS09565	serine/threonine transporter	-1.60	1.67E-05
DR76_RS09605	glucarate transporter	-1.75	2.54E-06
DR76_RS09610	glucarate dehydratase	-1.79	2.77E-06
DR76_RS09615	glucarate dehydratase	-1.57	1.99E-05
DR76_RS09650	hypothetical protein	-3.90	1.19E-14
DR76_RS09655	membrane protein	-16.84	4.23E-32
DR76_RS09660	membrane protein	-16.84	2.35E-28
DR76_RS09665	hypothetical protein	-16.84	2.53E-27
DR76_RS09675	hypothetical protein	-4.14	1.04E-08
DR76_RS09680	sugar kinase	-2.03	2.67E-07
DR76_RS09685	membrane protein	-1.44	4.61E-04
DR76_RS09690	oxidoreductase	-2.91	6.00E-06
DR76_RS09710	electron transfer flavoprotein	-1.17	3.52E-03
DR76_RS09715	hypothetical protein	-1.24	5.04E-04
DR76_RS09720	ferredoxin	-1.96	7.52E-06
DR76_RS09725	FAD-dependent oxidoreductase	-1.95	1.07E-07
DR76_RS09735	sulfite reductase subunit alpha	-2.15	6.20E-05
DR76_RS09740	sulfite reductase subunit beta	-1.82	3.56E-03
DR76_RS09745	phosphoadenosine phosphosulfate reductase	-1.50	4.63E-03
DR76_RS09750	protein hokG	-4.54	7.28E-27

DR76_RS09760	sulfate adenylyltransferase subunit 2	-3.13	1.06E-10
DR76_RS09765	sulfate adenylyltransferase subunit 1	-2.86	1.10E-09
DR76_RS09770	adenylylsulfate kinase	-2.77	1.48E-10
DR76_RS09775	membrane protein	-1.86	1.07E-05
DR76_RS09820	NADPH-dependent FMN reductase	-16.84	5.47E-24
DR76_RS09825	lactamase	-16.84	6.57E-16
DR76_RS09855	DeoR family transcriptional regulator	-1.03	1.89E-03
DR76_RS09860	serine/threonine protein phosphatase	-1.80	4.39E-04
DR76_RS09865	DNA mismatch repair protein MutS	-1.74	6.91E-06
DR76_RS09870	hypothetical protein	-1.56	9.93E-04
DR76_RS09875	transporter	-16.84	1.54E-44
DR76_RS09890	hydrogenase formation protein HypD	-2.12	1.94E-07
DR76_RS09895	hydrogenase assembly chaperone	-1.89	1.19E-06
DR76_RS09900	GTP hydrolase involved in nickel liganding into hydrogenases	-2.04	3.83E-06
DR76_RS09905	hydrogenase nickel incorporation protein	-2.25	4.88E-09
DR76_RS09910	formate hydrogenlyase regulatory protein HycA	-3.14	2.47E-06
DR76_RS09920	hydrogenase 3-2C Fe-S subunit	-2.88	1.30E-04
DR76_RS09925	formate hydrogenlyase subunit 3	-1.96	2.33E-04
DR76_RS09930	hydrogenase 3 membrane subunit	-2.97	3.93E-05
DR76_RS09935	hydrogenase 3 large subunit	-3.61	2.51E-06
DR76_RS09940	formate hydrogenlyase complex iron-sulfur subunit	-3.58	3.89E-05
DR76_RS09945	formate hydrogenlyase subunit 7	-1.93	1.47E-03
DR76_RS09955	hydrogenase 3 maturation protease	-2.52	3.45E-10
DR76_RS09960	hypothetical protein	-2.70	8.48E-10
DR76_RS09970	XRE family transcriptional regulator	-1.41	3.71E-04
DR76_RS09975	hypothetical protein	-16.84	5.17E-44
DR76_RS09980	formate dehydrogenase-H-2C [4Fe-4S] ferredoxin subunit	-2.58	1.86E-06
DR76_RS09985	carbamoyl phosphate phosphatase	-1.26	4.01E-04
DR76_RS10005	arabinose 5-phosphate isomerase	-1.84	8.72E-06
DR76_RS10020	sorbitol-6-phosphate dehydrogenase	-2.99	1.19E-04
DR76_RS10025	PTS sorbitol transporter subunit IIA	-3.61	5.88E-05
DR76_RS10030	PTS sorbitol transporter subunit IIB	-3.46	8.76E-05
DR76_RS10035	PTS sorbitol transporter subunit IIC	-4.24	1.55E-06
DR76_RS10045	nicotinamide-nucleotide amidohydrolase PncC	-1.60	1.52E-05
DR76_RS10050	recombinase RecA	-2.95	1.15E-07
DR76_RS10055	regulatory protein RecX	-2.16	3.78E-08
DR76_RS10060	membrane protein	-3.18	1.92E-05
DR76_RS10080	hypothetical protein	-2.42	1.08E-02
DR76_RS10085	hypothetical protein	-7.43	2.60E-23
DR76_RS10090	membrane protein	-8.11	2.12E-20
DR76_RS10095	hypothetical protein	-16.84	9.66E-17
DR76_RS10125	hypothetical protein	-16.84	2.98E-31
DR76_RS10130	membrane protein	-16.84	4.38E-26
DR76_RS10200	alkylhydroperoxidase	-16.84	1.60E-13

DR76_RS10205	DeoR family transcriptional regulator	-16.84	6.33E-34
DR76_RS10300	recombinase	-2.79	4.62E-07
DR76_RS10475	Elongation factor Tu 2	-1.86	3.94E-04
DR76_RS10575	transcriptional regulator	-1.53	7.26E-03
DR76_RS10670	hypothetical protein	-16.84	3.17E-18
DR76_RS10675	membrane protein	-7.26	4.97E-16
DR76_RS10685	(alpha)-aspartyl dipeptidase	-3.44	7.40E-07
DR76_RS10710	PTS friuctose transporter subunit IIB	-1.59	2.35E-02
DR76_RS10795	sugar ABC transporter substrate-binding protein	-2.80	1.05E-03
DR76_RS10805	sugar ABC transporter ATP-binding protein	-1.96	6.94E-03
DR76_RS10810	maltoporin	-1.77	1.10E-02
DR76_RS10815	maltose operon protein	-1.21	1.65E-02
DR76_RS10840	transcriptional repressor of SOS regulon	-1.44	4.07E-03
DR76_RS10875	oxidoreductase	-16.84	1.21E-35
DR76_RS10880	MFS transporter	-16.84	3.41E-27
DR76_RS10885	enoyl-CoA hydratase	-16.84	7.24E-22
DR76_RS10890	CoA-transferase	-16.84	9.80E-22
DR76_RS10895	regulator	-16.84	2.09E-56
DR76_RS10905	hypothetical protein	-16.84	3.03E-38
DR76_RS10910	alanine racemase	-1.65	3.80E-06
DR76_RS10915	nicotinamide mononucleotide transporter	-16.84	2.16E-24
DR76_RS10920	NAD metabolism ATPase/kinase	-16.84	3.55E-24
DR76_RS10930	2-oxoglutarate dehydrogenase E1	-9.03	3.80E-28
DR76_RS10935	dihydrolipoamide succinyltransferase	-16.84	3.68E-17
DR76_RS10940	dihydrolipoamide dehydrogenase	-16.84	1.54E-21
DR76_RS10945	malate--CoA ligase subunit beta	-16.84	9.44E-25
DR76_RS10950	succinyl-CoA synthetase subunit alpha	-16.84	3.63E-24
DR76_RS10955	membrane protein	-16.84	6.44E-08
DR76_RS10960	lactate dehydrogenase	-16.84	9.76E-21
DR76_RS10965	Fis family transcriptional regulator	-16.84	1.28E-24
DR76_RS10970	ATPase	-16.84	8.80E-27
DR76_RS10975	phosphotransferase	-2.06	6.13E-05
DR76_RS11030	transcriptional regulator	-16.84	8.10E-34
DR76_RS11035	LrgA	-16.84	1.01E-22
DR76_RS11040	LrgB	-16.84	4.19E-25
DR76_RS11065	nitrite reductase-2C formate-dependent-2C penta-heme cytochrome c	-1.34	4.40E-04
DR76_RS11070	formate-dependent nitrite reductase subunit NrfC	-1.24	6.90E-04
DR76_RS11075	formate-dependent nitrite reductase subunit NrfD	-1.12	2.56E-03
DR76_RS11105	peptide ABC transporter ATP-binding protein	-16.84	1.36E-25
DR76_RS11110	peptide ABC transporter ATP-binding protein	-16.84	6.62E-30
DR76_RS11115	peptide ABC transporter permease	-16.84	8.38E-27
DR76_RS11120	peptide ABC transporter permease	-16.84	1.74E-30
DR76_RS11125	ABC transporter substrate-binding protein	-11.99	8.69E-55
DR76_RS11130	formate dehydrogenase subunit alpha	-2.14	4.55E-05

DR76_RS11135	spermidine/putrescine ABC transporter substrate-binding protein	-2.06	4.02E-06
DR76_RS11215	ribose 1-2C5-bisphosphokinase	-1.29	2.38E-02
DR76_RS11225	phosphonate ABC transporter ATP-binding protein	-1.10	2.12E-02
DR76_RS11230	phosphonate C-P lyase	-2.54	1.25E-04
DR76_RS11260	phosphonate ABC transporter permease	-1.40	1.56E-02
DR76_RS11285	hypothetical protein	-1.49	7.09E-05
DR76_RS11290	hypothetical protein	-2.86	5.86E-12
DR76_RS11310	transcriptional regulator	-1.78	1.37E-06
DR76_RS11315	metal dependent hydrolase	-2.56	9.11E-10
DR76_RS11335	melibiose operon transcriptional regulator-3B autoregulator	-1.90	1.80E-07
DR76_RS11350	membrane protein	-1.40	3.55E-04
DR76_RS11355	fumarate hydratase	-3.74	1.59E-09
DR76_RS11360	C4-dicarboxylate ABC transporter	-3.55	3.43E-10
DR76_RS11410	lysine decarboxylase CadA	-1.21	5.48E-04
DR76_RS11415	putative cadaverine/lysine antiporter	-1.92	2.80E-05
DR76_RS11450	C4-dicarboxylate antiporter	-1.66	2.05E-03
DR76_RS11455	aspartate ammonia-lyase	-1.68	1.80E-03
DR76_RS11480	DUF4156 family lipoprotein	-1.08	3.31E-03
DR76_RS11485	hypothetical protein	-2.65	1.76E-10
DR76_RS11520	beta-lactamase	-1.80	1.59E-06
DR76_RS11525	fumarate reductase (anaerobic)-2C membrane anchor subunit	-1.70	8.88E-04
DR76_RS11530	fumarate reductase (anaerobic)-2C membrane anchor subunit	-2.06	6.18E-05
DR76_RS11535	fumarate reductase (anaerobic)-2C Fe-S subunit	-1.88	4.00E-04
DR76_RS11540	fumarate reductase flavoprotein subunit	-1.94	2.62E-04
DR76_RS11660	hypothetical protein	-2.33	8.38E-07
DR76_RS11665	PspA/IM30 family protein	-2.39	2.16E-08
DR76_RS11670	hypothetical protein	-3.11	5.19E-10
DR76_RS11675	membrane protein	-1.72	8.66E-04
DR76_RS11680	membrane protein	-2.25	7.47E-06
DR76_RS11715	L-ascorbate 6-phosphate lactonase	-1.89	2.70E-05
DR76_RS11720	PTS ascorbate transporter subunit IIC	-1.63	1.10E-04
DR76_RS11725	PTS ascorbate transporter subunit IIB	-2.01	1.37E-05
DR76_RS11730	PTS ascorbate transporter subunit IIA	-3.03	6.02E-10
DR76_RS11735	3-keto-L-gulonate-6-phosphate decarboxylase UlaD	-2.20	1.78E-07
DR76_RS11740	L-xylulose 5-phosphate 3-epimerase	-2.11	5.43E-07
DR76_RS11745	L-ribulose-5-phosphate 4-epimerase	-1.90	3.22E-06
DR76_RS11770	30S ribosomal protein S18	-1.00	9.71E-03
DR76_RS11775	50S ribosomal protein L9	-1.04	2.23E-02
DR76_RS11780	XRE family transcriptional regulator	-16.84	5.91E-20
DR76_RS11785	membrane protein	-16.84	3.27E-23
DR76_RS11790	hexuronate transporter ExuT	-16.84	1.82E-12
DR76_RS11830	peptidyl-prolyl cis-trans isomerase	-1.13	9.13E-03
DR76_RS11910	hypothetical protein	-16.84	3.74E-29
DR76_RS11965	anaerobic ribonucleotide reductase-activating protein	-1.05	9.61E-03

DR76_RS11970	ribonucleoside triphosphate reductase	-1.52	4.27E-03
DR76_RS11980	trehalose-6-phosphate hydrolase	-1.24	5.51E-03
DR76_RS12000	2-iminobutanoate/2-iminopropanoate deaminase	-1.17	2.27E-02
DR76_RS12020	ArgR family transcriptional regulator	-16.84	3.07E-20
DR76_RS12025	C4-dicarboxylate ABC transporter	-16.84	6.65E-30
DR76_RS12030	ornithine carbamoyltransferase	-16.84	1.66E-19
DR76_RS12035	carbamate kinase	-16.84	2.79E-16
DR76_RS12040	arginine deiminase	-16.84	2.81E-17
DR76_RS12045	hypothetical protein	-16.84	1.89E-28
DR76_RS12050	ornithine carbamoyltransferase	-4.86	2.97E-17
DR76_RS12060	acetyltransferase	-1.06	4.67E-03
DR76_RS12065	membrane protein	-16.84	1.02E-23
DR76_RS12260	hypothetical protein	-3.00	4.45E-05
DR76_RS12265	hemolysin activation protein	-7.05	1.76E-29
DR76_RS12270	Rha family transcriptional regulator	-16.84	2.89E-10
DR76_RS12275	hypothetical protein	-16.84	5.10E-11
DR76_RS12280	hypothetical protein	-16.84	7.21E-31
DR76_RS12285	hypothetical protein	-1.63	7.99E-04
DR76_RS12290	antigen 43	-3.38	1.30E-12
DR76_RS12295	hypothetical protein	-16.84	1.14E-03
DR76_RS12305	hypothetical protein	-4.38	3.78E-10
DR76_RS12310	hypothetical protein	-3.60	8.20E-07
DR76_RS12315	hypothetical protein	-16.84	1.06E-14
DR76_RS12320	hypothetical protein	-16.84	1.05E-14
DR76_RS12325	hypothetical protein	-16.84	3.22E-06
DR76_RS12330	antitoxin	-16.84	1.10E-18
DR76_RS12335	toxin	-16.84	8.58E-15
DR76_RS12340	hypothetical protein	-16.84	8.22E-22
DR76_RS12345	hypothetical protein	-16.84	1.57E-26
DR76_RS12350	transposase	-16.84	3.08E-24
DR76_RS12355	DNA methyltransferase	-16.84	3.37E-62
DR76_RS12360	cytosine methyltransferase	-16.84	9.04E-47
DR76_RS12365	type II restriction endonuclease	-16.84	2.72E-35
DR76_RS12370	restriction endonuclease	-16.84	1.31E-20
DR76_RS12375	restriction endonuclease	-16.84	5.19E-33
DR76_RS12380	hypothetical protein	-16.84	1.97E-05
DR76_RS12385	transposase	-16.84	6.22E-15
DR76_RS12390	hypothetical protein	-11.96	8.34E-48
DR76_RS12395	glycosyl transferase	-16.84	7.49E-10
DR76_RS12400	methylthioribose kinase	-16.84	4.30E-32
DR76_RS12405	methylthioribose-1-phosphate isomerase	-16.84	1.24E-30
DR76_RS12410	fuculose phosphate aldolase	-16.84	4.29E-24
DR76_RS12415	multidrug DMT transporter permease	-16.84	2.02E-27
DR76_RS12420	putative DNA-binding transcriptional regulator-3B KpLE2 phage-like element	-12.52	5.65E-58

DR76_RS12425	membrane protein	-16.84	5.37E-18
DR76_RS12430	putative transporter	-16.84	1.25E-31
DR76_RS12435	dehydratase	-16.84	3.95E-43
DR76_RS12440	putative lyase/synthase	-16.84	1.12E-34
DR76_RS12445	IclR family transcriptional regulator	-16.84	3.68E-25
DR76_RS12450	transcriptional regulator	-16.84	8.64E-26
DR76_RS12455	epimerase	-16.84	5.50E-24
DR76_RS12460	PTS sugar transporter subunit IIA	-16.84	1.74E-24
DR76_RS12465	putative nucleoside triphosphatase	-16.84	3.00E-37
DR76_RS12470	permase	-16.84	1.17E-26
DR76_RS12475	putative enzyme IIB component of PTS	-16.84	3.32E-16
DR76_RS12480	aminopeptidase	-9.84	9.92E-35
DR76_RS12485	hypothetical protein	-16.84	2.29E-35
DR76_RS12490	hypothetical protein	-16.84	4.08E-51
DR76_RS12495	N-acetyltransferase	-16.84	2.43E-46
DR76_RS12500	hypothetical protein	-16.84	3.46E-35
DR76_RS12505	membrane protein	-16.84	2.38E-08
DR76_RS12515	hypothetical protein	-16.84	9.41E-19
DR76_RS12545	transposase	-16.84	3.11E-38
DR76_RS12550	hypothetical protein	-16.84	4.47E-28
DR76_RS12560	type 1 fimbriae regulatory protein FimE	-4.27	7.16E-15
DR76_RS12605	fructuronate transporter	-6.18	2.90E-25
DR76_RS12615	mannonate dehydratase	-1.65	2.09E-05
DR76_RS12620	D-mannonate oxidoreductase	-1.83	1.25E-05
DR76_RS12690	hypothetical protein	-1.10	2.83E-03
DR76_RS12725	hypothetical protein	-16.84	1.12E-39
DR76_RS12730	DR76_RS14920	-12.85	3.00E-59
DR76_RS12735	restriction endonuclease subunit S	-16.84	2.62E-36
DR76_RS12740	DNA methyltransferase	-16.84	5.80E-55
DR76_RS12745	restriction endonuclease	-16.84	3.45E-54
DR76_RS12750	membrane protein	-16.84	4.31E-30
DR76_RS12755	hypothetical protein	-16.84	1.04E-29
DR76_RS12770	carbon starvation protein CstA	-1.75	2.77E-06
DR76_RS12775	hypothetical protein	-2.87	1.52E-04
DR76_RS12780	methyl-accepting chemotaxis protein	-3.30	2.56E-15
DR76_RS12790	GntR family transcriptional regulator	-2.49	7.85E-09
DR76_RS12865	membrane protein	-4.38	2.45E-08
DR76_RS13100	hypothetical protein	-1.15	6.65E-03
DR76_RS13135	hypothetical protein	-16.84	2.59E-06
DR76_RS13140	arylsulfatase	-16.84	1.51E-19
DR76_RS13145	hypothetical protein	-16.84	7.75E-21
DR76_RS13155	transcriptional regulator	-1.02	2.98E-03
DR76_RS13195	ribonucleoside hydrolase	-1.66	5.11E-05
DR76_RS13200	hypothetical protein	-16.84	1.16E-25

DR76_RS13220	carbamoyl phosphate synthase small subunit	-1.92	9.19E-06
DR76_RS13225	carbamoyl phosphate synthase large subunit	-1.71	1.83E-05
DR76_RS13235	transcriptional regulator	-1.08	4.45E-03
DR76_RS13240	carnitine operon protein CaiE	-2.55	4.42E-09
DR76_RS13245	carnitinyl-CoA dehydratase	-2.35	7.80E-06
DR76_RS13250	crotonobetaine/carnitine-CoA ligase	-1.11	1.60E-03
DR76_RS13255	crotonobetainyl-CoA:carnitine CoA-transferase	-1.54	3.95E-05
DR76_RS13260	crotonobetaine reductase subunit II-2C FAD-binding	-1.37	3.38E-04
DR76_RS13265	antiporter	-3.30	7.19E-15
DR76_RS13270	electron transfer flavoprotein FixB	-3.48	2.87E-15
DR76_RS13275	protein fixB	-2.94	4.49E-12
DR76_RS13280	putative oxidoreductase	-3.02	2.24E-12
DR76_RS13285	putative 4Fe-4S ferredoxin-type protein	-3.34	7.72E-08
DR76_RS13290	metabolite transporter	-2.67	1.35E-08
DR76_RS13310	antitoxin	-16.84	5.73E-35
DR76_RS13315	plasmid maintenance protein CcdB	-16.84	1.87E-32
DR76_RS13370	L-ribulose-5-phosphate 4-epimerase	-1.48	2.75E-04
DR76_RS13375	arabinose isomerase	-1.40	1.61E-04
DR76_RS13385	transcriptional regulator	-1.97	6.87E-08
DR76_RS13390	membrane protein	-16.84	7.14E-06
DR76_RS13395	hypothetical protein	-16.84	3.12E-07
DR76_RS13415	thiamine ABC transporter substrate-binding protein	-1.77	1.84E-06
DR76_RS13425	inhibitor of glucose transporter	-7.01	1.89E-23
DR76_RS13460	acetolactate synthase	-1.02	3.17E-03
DR76_RS13575	hypothetical protein	-16.84	8.63E-11
DR76_RS13580	transposase	-16.84	2.23E-14
DR76_RS13585	transposase	-16.84	1.18E-18
DR76_RS13650	HNH nuclease	-16.84	2.57E-31
DR76_RS13655	colicin immunity protein	-16.84	5.95E-17
DR76_RS13660	HNH endonuclease	-16.84	1.53E-11
DR76_RS13665	hypothetical protein	-16.84	3.44E-18
DR76_RS13670	hypothetical protein	-16.84	3.94E-31
DR76_RS13675	hypothetical protein	-16.84	5.28E-42
DR76_RS13680	transcriptional regulator	-1.02	7.54E-03
DR76_RS13710	hypothetical protein	-1.25	1.18E-03
DR76_RS13780	hypothetical protein	-16.84	4.40E-25
DR76_RS13785	pantoate--beta-alanine ligase	-1.04	7.47E-03
DR76_RS13790	3-methyl-2-oxobutanoate hydroxymethyltransferase	-3.63	2.24E-18
DR76_RS13795	fimbrial protein	-16.84	3.62E-43
DR76_RS13800	fimbrial protein	-16.84	1.34E-12
DR76_RS13805	fimbrial protein	-16.84	5.13E-12
DR76_RS13810	fimbrial protein StaD	-16.84	2.43E-11
DR76_RS13815	fimbrial protein	-16.84	8.35E-23
DR76_RS13820	molecular chaperone EcpD	-16.84	8.01E-12

DR76_RS13825	fimbrial protein	-16.84	2.99E-12
DR76_RS13830	2-amino-4-hydroxy-6-hydroxymethyldihydropteridine pyrophosphokinase	-1.30	1.94E-03
DR76_RS13870	ferrichrome outer membrane transporter	-2.90	2.66E-13
DR76_RS13925	serine endoprotease	-1.05	1.79E-02
DR76_RS14080	transposase	-16.84	4.57E-20
DR76_RS14210	membrane protein	-16.84	1.76E-25
DR76_RS14215	transposase	-16.84	3.32E-11
DR76_RS14220	isocitrate lyase	-16.84	2.93E-18
DR76_RS14225	transposase	-16.84	2.61E-23
DR76_RS14230	transposase	-16.84	1.85E-12
DR76_RS14235	transposase-2C IS116/IS110/IS902 family	-16.84	7.81E-42
DR76_RS14240	transposase	-16.84	2.70E-25
DR76_RS14245	membrane protein	-16.84	4.62E-24
DR76_RS14250	hypothetical protein	-16.84	5.76E-07
DR76_RS14255	malate transporter	-16.84	5.40E-11
DR76_RS14260	toxin	-16.84	2.11E-12
DR76_RS14265	antitoxin	-16.84	8.41E-18
DR76_RS14270	hypothetical protein	-4.84	5.13E-15
DR76_RS14275	hypothetical protein	-16.84	1.33E-10
DR76_RS14280	hypothetical protein	-16.84	3.57E-12
DR76_RS14285	hypothetical protein	-16.84	6.75E-11
DR76_RS14290	hypothetical protein	-16.84	6.12E-19
DR76_RS14295	hypothetical protein	-16.84	3.87E-20
DR76_RS14300	hypothetical protein	-16.84	6.38E-17
DR76_RS14305	hypothetical protein	-16.84	1.42E-26
DR76_RS14310	hypothetical protein	-16.84	1.84E-16
DR76_RS14315	hypothetical protein	-16.84	1.23E-18
DR76_RS14320	hypothetical protein	-16.84	1.28E-14
DR76_RS14325	phospholipase	-16.84	1.16E-20
DR76_RS14330	membrane protein	-16.84	1.06E-12
DR76_RS14335	hypothetical protein	-16.84	2.63E-27
DR76_RS14340	hypothetical protein	-16.84	1.02E-03
DR76_RS14370	hypothetical protein	-16.84	1.47E-49
DR76_RS14375	transposase	-16.84	2.07E-32
DR76_RS14380	transposase	-16.84	7.70E-33
DR76_RS14385	transposase	-16.84	1.23E-26
DR76_RS14390	transposase	-7.87	3.01E-20
DR76_RS14395	hypothetical protein	-16.84	8.42E-28
DR76_RS14400	Rha family transcriptional regulator	-16.84	7.02E-25
DR76_RS14405	transposase-2C IS116/IS110/IS902 family	-16.84	3.72E-38
DR76_RS14415	hypothetical protein	-1.16	1.35E-02
DR76_RS14420	hypothetical protein	-16.84	5.08E-25
DR76_RS14425	hypothetical protein	-2.58	4.05E-04
DR76_RS14435	hypothetical protein	-16.84	1.70E-13

DR76_RS14445	transposase	-8.87	2.31E-33
DR76_RS14450	hypothetical protein	-16.84	2.23E-22
DR76_RS14455	gamma-glutamyltranspeptidase	-16.84	8.08E-18
DR76_RS14460	phosphotriesterase	-16.84	4.20E-40
DR76_RS14465	membrane protein	-16.84	4.53E-30
DR76_RS14470	hypothetical protein	-16.84	2.99E-30
DR76_RS14475	ribokinase RbsK	-16.84	3.00E-31
DR76_RS14480	transposase	-16.84	1.27E-04
DR76_RS14485	membrane protein	-16.84	1.91E-17
DR76_RS14490	transferase	-16.84	2.10E-08
DR76_RS14495	membrane protein	-16.84	1.83E-15
DR76_RS14500	GlcNAc-PI de-N-acetylase	-16.84	1.71E-07
DR76_RS14505	hypothetical protein	-16.84	5.45E-58
DR76_RS14510	transcriptional regulator	-16.84	4.96E-41
DR76_RS14515	ligand-gated channel	-16.84	8.65E-49
DR76_RS14520	hypothetical protein	-16.84	4.19E-17
DR76_RS14550	hypothetical protein	-16.84	2.33E-24
DR76_RS14555	transposase	-16.84	2.23E-47
DR76_RS14560	transposase	-16.84	5.11E-54
DR76_RS14565	4'-phosphopantetheinyl transferase	-16.84	4.10E-62
DR76_RS14570	regulator	-16.84	1.43E-49
DR76_RS14575	amino acid adenylation protein	-16.84	6.13E-54
DR76_RS14580	polyketide synthase	-15.20	1.47E-41
DR76_RS14585	3-hydroxybutyryl-CoA dehydrogenase	-16.84	6.18E-39
DR76_RS14590	D-alanine--poly(phosphoribitol) ligase	-16.84	1.71E-38
DR76_RS14595	acyl-CoA dehydrogenase	-16.84	1.97E-35
DR76_RS14600	transacylase	-14.66	2.66E-34
DR76_RS14605	hypothetical protein	-14.99	1.62E-33
DR76_RS14610	polyketide synthase	-16.84	8.70E-36
DR76_RS14615	peptide synthetase	-15.99	2.42E-39
DR76_RS14620	peptide synthetase	-15.00	1.49E-44
DR76_RS14625	amidase	-16.84	6.22E-59
DR76_RS14630	transporter	-16.84	9.46E-60
DR76_RS14635	peptide synthetase	-16.84	2.62E-49
DR76_RS14640	polyketide synthase	-14.35	5.65E-49
DR76_RS14645	beta-lactamase	-16.84	2.66E-61
DR76_RS14650	thioesterase	-16.84	1.07E-56
DR76_RS14655	hypothetical protein	-14.65	1.40E-48
DR76_RS14660	hypothetical protein	-16.84	2.68E-57
DR76_RS14665	phage integrase	-16.84	2.14E-53
DR76_RS14670	membrane protein	-2.95	4.86E-09
DR76_RS14700	UPF0082 family protein	-1.22	2.26E-03
DR76_RS14735	hypothetical protein	-16.84	5.62E-08
DR76_RS14740	ligand-gated channel protein	-16.84	4.57E-24

DR76_RS14745	salicyl-AMP ligase	-16.84	2.18E-16
DR76_RS14750	thioesterase	-16.84	3.56E-08
DR76_RS14755	oxidoreductase	-16.84	1.22E-13
DR76_RS14760	polyketide synthase	-16.84	8.44E-37
DR76_RS14765	peptide synthetase	-16.84	9.09E-36
DR76_RS14770	AraC family transcriptional regulator	-16.84	3.40E-21
DR76_RS14775	ABC transporter ATP-binding protein	-16.84	5.81E-18
DR76_RS14780	ABC transporter permease	-16.84	2.84E-23
DR76_RS14785	MFS transporter	-16.84	5.22E-16
DR76_RS14790	salicylate synthase	-16.84	4.72E-26
DR76_RS14795	integrase	-16.84	4.09E-58
DR76_RS14800	membrane protein	-2.72	1.64E-09
DR76_RS14810	membrane protein	-1.54	6.55E-05
DR76_RS14815	hypothetical protein	-16.84	6.01E-55
DR76_RS14820	regulatory protein	-16.84	1.88E-04
DR76_RS14825	hypothetical protein	-16.84	6.58E-03
DR76_RS14835	DNA-binding protein	-16.84	1.14E-03
DR76_RS14840	hypothetical protein	-16.84	6.01E-27
DR76_RS14845	DNA-binding protein	-16.84	1.10E-47
DR76_RS14850	hypothetical protein	-16.84	2.74E-04
DR76_RS14855	hypothetical protein	-16.84	6.42E-22
DR76_RS14860	hypothetical protein	-16.84	1.47E-12
DR76_RS14865	hypothetical protein	-11.76	8.63E-52
DR76_RS14870	hypothetical protein	-16.84	8.97E-43
DR76_RS14875	membrane protein	-16.84	2.89E-09
DR76_RS14880	hypothetical protein	-16.84	1.18E-09
DR76_RS14885	hypothetical protein	-16.84	1.57E-08
DR76_RS14890	membrane protein	-16.84	1.97E-07
DR76_RS14895	hypothetical protein	-16.84	2.12E-24
DR76_RS14900	molecular chaperone Tir	-16.84	1.08E-26
DR76_RS14905	hypothetical protein	-16.84	5.30E-41
DR76_RS14910	conjugal transfer protein	-16.84	2.66E-19
DR76_RS14920	type IV pilin	-16.84	6.78E-08
DR76_RS14925	PilV	-16.84	7.58E-13
DR76_RS14930	hypothetical protein	-16.84	2.02E-43
DR76_RS14935	integrase	-16.84	5.07E-53
DR76_RS15055	flagellar biosynthesis protein FliR	-1.17	2.94E-03
DR76_RS15065	flagellar biosynthesis protein FliP	-1.49	6.92E-03
DR76_RS15070	flagellar biosynthesis protein FliO	-1.79	1.66E-03
DR76_RS15075	flagellar motor switch protein FliN	-2.45	4.66E-05
DR76_RS15080	flagellar motor switch protein FliM	-1.18	7.52E-03
DR76_RS15115	flagellar MS-ring protein	-1.95	2.26E-04
DR76_RS15140	kinase inhibitor	-1.56	1.04E-04
DR76_RS15145	transcriptional regulator	-1.42	1.84E-03

DR76_RS15150	outer membrane porin protein C	-15.25	1.52E-49
DR76_RS15165	membrane protein	-1.18	1.85E-03
DR76_RS15180	flagellar biosynthesis protein FliT	-4.43	3.21E-19
DR76_RS15185	flagellar biosynthesis protein FliS	-5.59	1.55E-24
DR76_RS15190	flagellar capping protein	-5.82	1.17E-23
DR76_RS15195	flagellin	-8.93	1.97E-28
DR76_RS15200	RNA polymerase-2C sigma 28 (sigma F) factor	-5.46	5.58E-31
DR76_RS15205	flagellar biosynthesis protein FliZ	-3.53	1.34E-15
DR76_RS15265	membrane protein	-1.46	1.87E-03
DR76_RS15270	membrane protein	-2.00	1.91E-05
DR76_RS15290	ferritin iron storage protein (cytoplasmic)	-2.10	1.76E-05
DR76_RS15310	ferritin	-1.58	1.36E-03
DR76_RS15315	arabinose ABC transporter substrate-binding protein	-2.19	1.92E-08
DR76_RS15320	L-arabinose ABC transporter ATPase	-1.06	3.01E-03
DR76_RS15345	flagellar class II regulon transcriptional activator-2C with FlhC	-2.04	2.94E-08
DR76_RS15350	flagellar class II regulon transcriptional activator-2C with FlhD	-1.74	3.44E-06
DR76_RS15355	flagellar motor protein MotA	-4.69	1.82E-23
DR76_RS15360	flagellar motor protein MotB	-4.66	5.11E-22
DR76_RS15365	chemotaxis protein CheA	-4.74	1.13E-22
DR76_RS15370	purine-binding chemotaxis protein	-3.75	7.46E-18
DR76_RS15375	methyl-accepting chemotaxis protein	-5.79	4.01E-24
DR76_RS15380	membrane protein	-7.89	8.50E-31
DR76_RS15385	chemotaxis protein CheR	-4.63	2.34E-24
DR76_RS15390	chemotaxis response regulator protein-glutamate methylesterase	-5.11	7.42E-30
DR76_RS15395	chemotaxis protein CheY	-4.69	9.06E-25
DR76_RS15400	chemotaxis protein CheZ	-4.77	6.25E-22
DR76_RS15405	flagellar biosynthesis protein FlhB	-1.88	4.13E-05
DR76_RS15415	flagellar protein flhE	-1.65	9.56E-04
DR76_RS15495	component of RuvABC resolvasome-2C regulatory subunit	-1.35	4.55E-04
DR76_RS15500	Holliday junction DNA helicase RuvB	-1.02	8.66E-03
DR76_RS15555	KHG/KDPG aldolase-3B 2-dehydro-3-deoxy-phosphogluconate/4-hydroxy-2-oxoglutarate aldolase	-1.05	1.25E-02
DR76_RS15565	DNA damage-inducible protein YebG	-1.22	1.22E-02
DR76_RS15575	hypothetical protein	-1.22	1.42E-03
DR76_RS15625	hypothetical protein	-3.65	4.76E-08
DR76_RS15715	stress protein-2C member of the CspA-family	-1.17	2.60E-02
DR76_RS15725	putative Mn(2+) efflux pump-2C mntR-regulated	-1.89	1.41E-06
DR76_RS15730	hypothetical protein	-3.86	6.35E-16
DR76_RS15795	hypothetical protein	-1.36	2.47E-02
DR76_RS15800	putative reactive intermediate deaminase	-1.62	3.67E-05
DR76_RS15875	hypothetical protein	-1.09	1.48E-03
DR76_RS15955	alcohol dehydrogenase	-16.84	1.27E-23
DR76_RS15960	transporter	-16.84	5.28E-19
DR76_RS15965	alcohol dehydrogenase	-16.84	1.76E-10
DR76_RS15970	hypothetical protein	-16.84	3.61E-13

DR76_RS15975	sugar kinase	-16.84	2.18E-14
DR76_RS15980	oxidoreductase	-16.84	1.90E-24
DR76_RS15985	DeoR family transcriptional regulator	-16.84	2.16E-30
DR76_RS15990	MFS transporter	-16.84	1.02E-23
DR76_RS16035	hypothetical protein	-16.84	4.19E-14
DR76_RS16040	hypothetical protein	-16.84	1.48E-10
DR76_RS16045	molecular chaperone DnaJ	-16.84	6.79E-20
DR76_RS16070	thiosulfate sulfurtransferase	-1.31	2.11E-04
DR76_RS16075	sulfate ABC transporter ATP-binding protein	-1.97	3.45E-06
DR76_RS16100	hypothetical protein	-1.44	8.01E-04
DR76_RS16150	nucleotide excision repair endonuclease	-1.20	9.17E-03
DR76_RS16205	cell division modulator	-2.38	2.64E-08
DR76_RS16210	L-cystine transporter tcyP	-2.15	3.87E-05
DR76_RS16390	putative acyl-CoA dehydrogenase	-1.56	1.89E-03
DR76_RS16395	hypothetical protein	-2.07	5.43E-03
DR76_RS16410	transporter	-1.39	1.73E-02
DR76_RS16495	hypothetical protein	-3.94	2.87E-13
DR76_RS16500	oxidoreductase	-3.70	6.57E-08
DR76_RS16505	hypothetical protein	-2.94	6.31E-07
DR76_RS16510	ferredoxin	-2.80	3.71E-06
DR76_RS16515	thiosulfate reductase cytochrome B	-2.27	2.04E-04
DR76_RS16520	hypothetical protein	-1.45	1.00E-02
DR76_RS16535	hypothetical protein	-2.46	3.26E-05
DR76_RS16670	anhydro-N-acetylmuramic acid kinase	-1.01	5.64E-03
DR76_RS16765	adenosine deaminase	-1.35	1.20E-03
DR76_RS16780	LacI family transcriptional regulator	-2.29	1.77E-09
DR76_RS16860	hypothetical protein	-1.10	1.94E-02
DR76_RS16935	dimethyl sulfoxide reductase subunit H	-1.77	2.34E-04
DR76_RS16940	oxidoreductase-2C Fe-S subunit	-2.45	4.08E-08
DR76_RS16945	DmsA/YnfE family anaerobic dimethyl sulfoxide reductase-2C A subunit	-2.38	1.13E-05
DR76_RS16950	dimethyl sulfoxide reductase subunit A	-2.70	9.61E-08
DR76_RS16980	bifunctional D-altronate/D-mannonate dehydratase	-2.03	2.03E-04
DR76_RS17005	Qin prophage-3B uncharacterized protein	-16.84	1.43E-02
DR76_RS17010	Qin prophage-3B cell division inhibition protein	-16.84	4.53E-03
DR76_RS17015	hypothetical protein	-16.84	2.26E-30
DR76_RS17020	hypothetical protein	-16.84	3.59E-10
DR76_RS17025	transcriptional regulator	-10.60	2.94E-39
DR76_RS17030	transcriptional regulator	-16.84	5.66E-03
DR76_RS17035	hypothetical protein	-6.37	1.93E-11
DR76_RS17040	hypothetical protein	-2.82	5.39E-07
DR76_RS17045	hypothetical protein	-16.84	2.70E-21
DR76_RS17050	DR76_RS14920	-16.84	4.75E-58
DR76_RS17055	hypothetical protein	-2.08	8.12E-05
DR76_RS17060	Regulatory protein mokC	-4.31	2.85E-13

DR76_RS17085	membrane protein	-16.84	1.03E-15
DR76_RS17090	cold shock-like protein CspB	-13.40	1.98E-37
DR76_RS17095	Qin prophage-3B putative S lysis protein	-16.84	2.93E-05
DR76_RS17100	hypothetical protein	-16.84	1.55E-06
DR76_RS17105	Qin prophage-3B putative lysozyme	-16.84	1.64E-09
DR76_RS17110	Rz-like protein-2C Qin prophage	-16.84	1.78E-16
DR76_RS17115	cold shock-like protein CspI	-15.19	7.77E-47
DR76_RS17120	hypothetical protein	-16.84	9.96E-37
DR76_RS17125	Qin prophage-3B multicopy suppressor of secG(Cs) and fabA6(Ts)	-16.84	1.63E-46
DR76_RS17130	hypothetical protein	-16.84	1.32E-11
DR76_RS17135	hypothetical protein	-16.84	2.88E-28
DR76_RS17140	membrane protein	-16.84	7.76E-16
DR76_RS17145	hypothetical protein	-16.84	3.60E-27
DR76_RS17150	DNA packaging protein	-3.82	1.64E-16
DR76_RS17155	hypothetical protein	-16.84	1.30E-11
DR76_RS17160	capsid protein	-16.84	2.15E-26
DR76_RS17165	peptidase S14	-16.84	2.26E-26
DR76_RS17170	hypothetical protein	-16.84	1.19E-08
DR76_RS17175	DNA breaking-rejoining protein	-16.84	6.39E-07
DR76_RS17180	tail protein	-16.84	7.24E-13
DR76_RS17185	tail protein	-16.84	5.66E-12
DR76_RS17190	tail protein	-16.84	9.77E-25
DR76_RS17195	phage minor tail protein G	-16.84	1.66E-09
DR76_RS17200	tail protein	-16.84	1.10E-07
DR76_RS17205	hypothetical protein	-16.84	1.01E-32
DR76_RS17210	tail protein	-16.84	2.35E-10
DR76_RS17215	tail protein	-16.84	3.19E-17
DR76_RS17220	tail protein	-16.84	2.47E-11
DR76_RS17225	tail protein	-16.84	6.39E-07
DR76_RS17230	phage tail component	-4.43	3.13E-17
DR76_RS17245	hypothetical protein	-16.84	4.31E-07
DR76_RS17250	hypothetical protein	-16.84	5.45E-16
DR76_RS17255	phage tail fiber repeat-containing domain protein	-16.84	2.87E-21
DR76_RS17260	tail assembly protein	-16.84	1.14E-17
DR76_RS17265	DNA invertase	-16.84	2.89E-43
DR76_RS17270	cold shock protein-2C function unknown-2C Qin prophage	-11.13	2.87E-43
DR76_RS17320	inner membrane-associated protein	-1.09	6.41E-03
DR76_RS17340	PTS lactose transporter subunit IIB	-5.55	1.07E-11
DR76_RS17345	PTS cellobiose transporter subunit IIC	-2.67	2.18E-06
DR76_RS17355	porin	-2.04	3.83E-04
DR76_RS17395	membrane protein	-5.44	1.93E-15
DR76_RS17400	glutaminase	-1.71	8.95E-06
DR76_RS17405	hypothetical protein	-1.96	1.29E-06
DR76_RS17445	hypothetical protein	-16.84	5.61E-26

DR76_RS17450	LysR family transcriptional regulator	-16.84	4.72E-38
DR76_RS17455	NAD-dependent dehydratase	-16.84	1.39E-15
DR76_RS17460	hypothetical protein	-16.84	1.12E-02
DR76_RS17465	fimbrial protein	-1.30	4.64E-04
DR76_RS17470	hypothetical protein	-1.08	9.64E-03
DR76_RS17510	sulfatase	-4.25	1.68E-09
DR76_RS17555	diguanylate cyclase	-2.94	4.61E-09
DR76_RS17590	hypothetical protein	-16.84	1.02E-34
DR76_RS17605	formate dehydrogenase-N subunit beta	-1.58	4.70E-04
DR76_RS17610	formate dehydrogenase subunit alpha	-1.74	1.05E-05
DR76_RS17615	sulfate ABC transporter substrate-binding protein	-1.51	1.49E-04
DR76_RS17655	N-hydroxyarylamine O-acetyltransferase	-1.46	1.18E-04
DR76_RS17665	hypothetical protein	-16.84	3.25E-03
DR76_RS17670	acetyltransferase	-16.84	2.11E-09
DR76_RS17675	hypothetical protein	-16.84	6.28E-08
DR76_RS17680	hypothetical protein	-16.84	1.10E-03
DR76_RS17690	hypothetical protein	-16.84	1.65E-06
DR76_RS17695	peptidase M35	-16.84	1.02E-07
DR76_RS17700	type IV secretion protein Rhs	-16.84	2.51E-24
DR76_RS17705	hypothetical protein	-16.84	3.02E-15
DR76_RS17710	hypothetical protein	-16.84	7.68E-38
DR76_RS17715	hypothetical protein	-16.84	4.23E-27
DR76_RS17720	hypothetical protein	-16.84	1.29E-21
DR76_RS17725	type IV secretion protein Rhs	-16.84	1.35E-27
DR76_RS17730	hypothetical protein	-16.84	1.03E-13
DR76_RS17760	GntR family transcriptional regulator	-2.67	7.50E-07
DR76_RS17850	tellurite resistance protein TehB	-1.41	1.45E-04
DR76_RS17865	ribosomal-protein-L7/L12-serine acetyltransferase	-1.76	5.57E-06
DR76_RS17870	hypothetical protein	-1.99	1.78E-03
DR76_RS17880	glucan biosynthesis protein D	-1.34	2.80E-03
DR76_RS17935	FMN-dependent NADH-azoreductase	-2.47	1.93E-08
DR76_RS17950	hypothetical protein	-1.39	1.15E-02
DR76_RS17955	membrane protein	-2.35	6.01E-06
DR76_RS17960	oxidoreductase	-1.02	2.29E-03
DR76_RS17965	hypothetical protein	-16.84	7.81E-55
DR76_RS18010	universal stress protein F	-1.96	4.28E-04
DR76_RS18015	hypothetical protein	-16.84	2.39E-04
DR76_RS18020	hypothetical protein	-16.84	1.80E-09
DR76_RS18025	integrase	-16.84	2.93E-20
DR76_RS18060	drug resistance transporter EmrB/QacA subfamily protein	-16.84	3.33E-10
DR76_RS18065	HlyD family secretion protein	-16.84	2.74E-04
DR76_RS18070	regulator	-16.84	1.23E-27
DR76_RS18075	glutamate decarboxylase	-16.84	1.04E-11
DR76_RS18080	pump protein	-5.92	1.56E-09

DR76_RS18130	NAD-dependent dehydratase	-3.46	3.26E-13
DR76_RS18165	LacI family transcriptional regulator	-1.17	1.27E-03
DR76_RS18170	membrane protein	-1.23	1.16E-02
DR76_RS18215	sugar ABC transporter substrate-binding protein	-1.34	2.60E-03
DR76_RS18220	sucrose phosphorylase	-1.43	2.20E-03
DR76_RS18225	thiosulfate:cyanide sulfurtransferase	-3.63	6.20E-08
DR76_RS18250	transcriptional regulator	-1.30	7.26E-04
DR76_RS18255	hypothetical protein	-2.00	3.22E-04
DR76_RS18295	major facilitator transporter	-16.84	1.99E-30
DR76_RS18300	Multidrug transporter	-16.81	2.74E-24
DR76_RS18305	multidrug transporter	-16.84	4.91E-27
DR76_RS18310	acriflavine resistance protein E	-16.84	5.77E-18
DR76_RS18315	hypothetical protein	-16.84	3.02E-22
DR76_RS18405	hypothetical protein	-1.19	4.29E-03
DR76_RS18410	hypothetical protein	-1.48	3.84E-04
DR76_RS18460	hypothetical protein	-16.84	5.00E-42
DR76_RS18475	anthranilate synthase subunit I	-3.53	6.60E-05
DR76_RS18480	anthranilate phosphoribosyltransferase	-2.77	4.97E-03
DR76_RS18510	outer membrane protein W	-1.89	2.75E-04
DR76_RS18595	membrane protein	-16.84	3.14E-26
DR76_RS18640	membrane protein	-2.36	1.11E-07
DR76_RS18645	linoleoyl-CoA desaturase	-9.93	3.32E-34
DR76_RS18650	hypothetical protein	-16.84	1.34E-23
DR76_RS18655	acid phosphatase	-16.84	2.84E-14
DR76_RS18660	coenzyme F390 synthetase	-16.84	4.31E-28
DR76_RS18665	metallo-beta-lactamase	-16.84	2.11E-21
DR76_RS18670	hypothetical protein	-16.84	4.36E-36
DR76_RS18675	3-oxoacyl-ACP synthase	-16.84	4.16E-29
DR76_RS18725	hypothetical protein	-1.07	4.46E-03
DR76_RS18745	hypothetical protein	-1.19	1.62E-03
DR76_RS18815	hypothetical protein	-16.84	3.87E-31
DR76_RS18830	dihydroxyacetone kinase subunit DhaL	-1.24	5.86E-04
DR76_RS18845	ABC transporter ATP-binding protein	-16.84	4.43E-19
DR76_RS18850	peptide ABC transporter substrate-binding protein	-16.84	3.06E-18
DR76_RS18855	iron ABC transporter ATP-binding protein	-16.84	1.08E-17
DR76_RS18860	methyltransferase	-16.84	8.40E-17
DR76_RS18865	molybdenum transporter	-16.84	4.53E-17
DR76_RS18870	TonB-dependent receptor	-9.70	1.19E-25
DR76_RS18880	flagellar brake protein YcgR	-2.55	3.56E-07
DR76_RS18925	disulfide bond formation protein DsbB	-1.01	2.80E-03
DR76_RS18930	DNA polymerase V subunit UmuC	-3.16	1.26E-14
DR76_RS18935	DNA polymerase V subunit UmuD	-2.83	4.72E-13
DR76_RS18995	UPF0757 family protein	-2.33	1.67E-08
DR76_RS19000	uncharacterized protein	-1.54	4.35E-05

DR76_RS19005	hypothetical protein	-1.90	2.04E-05
DR76_RS19010	ATPase	-16.84	9.90E-15
DR76_RS19015	ATPase	-2.59	6.75E-05
DR76_RS19035	hypothetical protein	-2.53	1.65E-05
DR76_RS19070	hypothetical protein	-3.05	3.38E-03
DR76_RS19075	hypothetical protein	-7.05	1.82E-21
DR76_RS19080	hypothetical protein	-16.84	5.78E-44
DR76_RS19085	hypothetical protein	-16.84	1.55E-20
DR76_RS19110	hypothetical protein	-9.97	2.48E-34
DR76_RS19115	hypothetical protein	-16.84	8.73E-33
DR76_RS19120	phage tail protein	-16.84	3.31E-61
DR76_RS19125	phage tail protein	-6.20	2.78E-37
DR76_RS19130	tail assembly protein	-16.84	4.04E-42
DR76_RS19135	endopeptidase	-16.84	1.59E-42
DR76_RS19140	tail protein	-16.84	1.92E-41
DR76_RS19145	tail protein	-16.84	1.66E-21
DR76_RS19150	hypothetical protein	-16.84	1.93E-61
DR76_RS19155	tail protein	-16.84	2.20E-42
DR76_RS19160	tail protein	-16.84	2.49E-45
DR76_RS19165	tail protein	-16.84	1.67E-55
DR76_RS19170	tail protein	-16.84	2.37E-49
DR76_RS19175	tail protein	-16.84	9.65E-52
DR76_RS19180	tail attachment protein	-16.84	3.69E-45
DR76_RS19185	hypothetical protein	-16.84	1.08E-17
DR76_RS19190	hypothetical protein	-16.84	6.74E-53
DR76_RS19195	Head decoration protein	-16.84	2.23E-57
DR76_RS19200	scaffolding protein	-16.84	1.14E-37
DR76_RS19215	terminase	-16.84	5.08E-57
DR76_RS19220	terminase	-16.84	1.19E-47
DR76_RS19225	DNA-packaging protein	-16.84	1.53E-10
DR76_RS19230	hypothetical protein	-16.84	1.87E-11
DR76_RS19235	hypothetical protein	-16.84	1.30E-06
DR76_RS19240	cell envelope biogenesis protein TonB	-16.84	1.07E-22
DR76_RS19245	hypothetical protein	-16.84	1.55E-44
DR76_RS19250	endopeptidase	-16.84	6.94E-49
DR76_RS19255	lysozyme	-4.55	1.29E-19
DR76_RS19260	DLP12 prophage-3B putative phage lysis protein	-7.28	3.86E-24
DR76_RS19265	outer membrane porin protein C	-14.76	2.57E-48
DR76_RS19270	antitermination protein	-4.71	3.45E-21
DR76_RS19275	hypothetical protein	-3.93	2.40E-12
DR76_RS19280	DLP12 prophage-3B endonuclease RUS	-3.85	2.39E-17
DR76_RS19285	Protein ninF	-16.84	3.45E-27
DR76_RS19290	protein ninX	-16.84	2.99E-40
DR76_RS19295	protein ninE	-16.84	6.66E-26

DR76_RS19300	DNA methylase	-16.84	1.82E-46
DR76_RS19305	hypothetical protein	-16.84	6.65E-51
DR76_RS19310	protein ren	-16.84	2.40E-51
DR76_RS19315	Replication protein 14	-3.56	2.02E-18
DR76_RS19320	hypothetical protein	-4.37	4.31E-23
DR76_RS19325	regulatory protein	-16.84	1.73E-57
DR76_RS19330	CRO	-16.84	2.04E-62
DR76_RS19335	prophage repressor protein	-16.84	2.14E-55
DR76_RS19340	hypothetical protein	-13.03	1.63E-49
DR76_RS19345	hypothetical protein	-16.84	7.21E-51
DR76_RS19350	hypothetical protein	-16.84	1.62E-09
DR76_RS19355	hypothetical protein	-16.84	2.47E-15
DR76_RS19360	antitermination protein N	-16.84	7.94E-53
DR76_RS19365	superinfection exclusion protein B	-16.84	6.22E-48
DR76_RS19370	transposase	-16.84	2.17E-30
DR76_RS19375	membrane protein	-16.84	2.52E-41
DR76_RS19380	Restriction inhibitor protein ral	-16.84	4.57E-43
DR76_RS19385	Lambda prophage-derived protein ea10	-16.84	4.92E-45
DR76_RS19390	regulatory protein	-16.84	1.24E-48
DR76_RS19395	hypothetical protein	-7.18	1.79E-31
DR76_RS19400	host-nuclease inhibitor protein Gam	-1.42	1.72E-03
DR76_RS19405	recombinase	-1.66	2.59E-03
DR76_RS19410	exonuclease	-1.87	1.00E-03
DR76_RS19415	hypothetical protein	-1.64	1.71E-03
DR76_RS19420	hypothetical protein	-2.63	2.72E-06
DR76_RS19425	-	-16.84	1.70E-16
DR76_RS19430	transposase	-16.84	2.59E-20
DR76_RS19435	hypothetical protein	-4.28	2.60E-11
DR76_RS19440	hypothetical protein	-1.50	1.29E-03
DR76_RS19445	conjugal transfer protein TraR	-2.75	4.86E-08
DR76_RS19450	hypothetical protein	-10.20	1.66E-44
DR76_RS19455	hypothetical protein	-4.73	2.02E-19
DR76_RS19460	hypothetical protein	-16.84	7.41E-48
DR76_RS19465	hypothetical protein	-16.84	1.37E-38
DR76_RS19470	hypothetical protein	-16.84	1.11E-53
DR76_RS19475	RNA-binding protein	-16.84	2.85E-43
DR76_RS19480	hypothetical protein	-7.88	3.08E-33
DR76_RS19485	excisionase	-7.46	2.59E-28
DR76_RS19490	integrase	-6.13	1.22E-20
DR76_RS19540	peptidase T	-1.34	4.92E-03
DR76_RS19545	hypothetical protein	-1.77	1.52E-04
DR76_RS19550	spermidine/putrescine ABC transporter ATPase	-1.50	3.26E-05
DR76_RS19555	putrescine/spermidine ABC transporter permease	-1.28	1.05E-03
DR76_RS19560	hypothetical protein	-3.10	9.52E-10

DR76_RS19565	hypothetical protein	-2.07	1.15E-06
DR76_RS19570	tail assembly protein	-4.27	2.66E-17
DR76_RS19575	membrane protein	-16.84	4.28E-46
DR76_RS19580	hypothetical protein	-16.84	9.73E-28
DR76_RS19585	Host specificity protein J	-16.84	6.60E-49
DR76_RS19590	hypothetical protein	-16.84	2.31E-10
DR76_RS19595	tail assembly protein	-16.84	6.38E-23
DR76_RS19600	tail protein	-16.84	1.25E-22
DR76_RS19605	tail protein	-16.84	1.93E-21
DR76_RS19610	tail protein	-16.84	4.47E-11
DR76_RS19615	lambda family phage tail tape measure protein	-16.84	2.71E-29
DR76_RS19620	tail protein	-16.84	9.26E-14
DR76_RS19625	tail protein	-16.84	3.53E-18
DR76_RS19630	phage tail protein	-16.84	4.71E-25
DR76_RS19635	tail protein	-16.84	8.21E-18
DR76_RS19640	tail protein	-16.84	5.31E-21
DR76_RS19645	tail attachment protein	-16.84	3.65E-16
DR76_RS19650	hypothetical protein	-16.84	1.76E-04
DR76_RS19655	head protein	-16.84	6.62E-10
DR76_RS19665	hypothetical protein	-16.84	1.00E-58
DR76_RS19670	plasmid partitioning protein ParB	-16.84	2.91E-53
DR76_RS19675	head-tail joining protein	-16.84	2.09E-23
DR76_RS19680	terminase	-16.84	9.24E-39
DR76_RS19685	terminase	-16.84	6.71E-27
DR76_RS19690	DNA-packaging protein	-16.84	2.00E-13
DR76_RS19695	hypothetical protein	-16.84	1.84E-04
DR76_RS19710	hypothetical protein	-16.84	2.46E-10
DR76_RS19715	endopeptidase	-16.84	7.31E-18
DR76_RS19720	membrane protein	-16.84	1.76E-04
DR76_RS19725	membrane protein	-16.84	2.00E-08
DR76_RS19730	lysozyme	-16.84	1.18E-20
DR76_RS19735	hypothetical protein	-16.84	1.25E-27
DR76_RS19740	holin	-16.84	5.79E-11
DR76_RS19745	hypothetical protein	-16.84	4.95E-17
DR76_RS19750	hypothetical protein	-16.84	5.07E-15
DR76_RS19755	hypothetical protein	-16.84	1.77E-25
DR76_RS19760	hypothetical protein	-10.34	5.73E-39
DR76_RS19765	anti-adapter protein IraM	-16.84	1.89E-24
DR76_RS19770	hypothetical protein	-16.84	9.60E-50
DR76_RS19775	hypothetical protein	-16.84	6.56E-11
DR76_RS19780	hypothetical protein	-16.84	5.07E-12
DR76_RS19785	endodeoxyribonuclease	-1.78	7.17E-03
DR76_RS19795	hypothetical protein	-7.25	7.69E-20
DR76_RS19800	Protein hokC	-1.56	1.72E-03

DR76_RS19805	hypothetical protein	-8.28	1.09E-41
DR76_RS19810	membrane protein	-16.84	8.22E-22
DR76_RS19815	hypothetical protein	-16.84	5.05E-13
DR76_RS19830	hypothetical protein	-16.84	5.17E-11
DR76_RS19835	hypothetical protein	-16.84	1.01E-07
DR76_RS19840	hypothetical protein	-16.84	9.31E-03
DR76_RS19845	hypothetical protein	-16.84	7.08E-11
DR76_RS19850	membrane protein	-3.71	1.64E-07
DR76_RS19855	hypothetical protein	-16.84	1.12E-25
DR76_RS19860	DNA replication protein DnaC	-16.84	3.37E-23
DR76_RS19865	DNA-binding protein	-16.84	3.77E-28
DR76_RS19870	Rha family transcriptional regulator	-16.84	1.61E-25
DR76_RS19875	hypothetical protein	-16.84	9.09E-21
DR76_RS19880	repressor	-11.09	1.68E-47
DR76_RS19885	hypothetical protein	-16.84	1.09E-52
DR76_RS19890	plasmid stabilization protein ParE	-16.84	4.90E-59
DR76_RS19895	phage protein	-16.84	6.95E-25
DR76_RS19900	hypothetical protein	-16.84	4.34E-26
DR76_RS19905	hypothetical protein	-16.84	3.85E-34
DR76_RS19910	membrane protein	-16.84	1.94E-23
DR76_RS19915	cell division inhibitor	-16.84	4.49E-10
DR76_RS19920	hypothetical protein	-16.84	7.77E-13
DR76_RS19925	exodeoxyribonuclease VIII	-7.44	1.35E-21
DR76_RS19930	excisionase	-16.84	3.09E-15
DR76_RS19935	integrase	-16.84	2.85E-31
DR76_RS19940	putrescine/spermidine ABC transporter permease	-1.12	2.44E-03
DR76_RS19945	putrescine/spermidine ABC transporter substrate-binding protein	-1.18	2.17E-03
DR76_RS19950	membrane protein	-16.84	4.44E-24
DR76_RS19955	membrane protein	-4.02	2.17E-16
DR76_RS19985	membrane protein	-3.15	1.80E-13
DR76_RS20015	NADH dehydrogenase	-1.12	3.34E-03
DR76_RS20145	flagellar hook-associated protein FlgL	-2.50	2.74E-10
DR76_RS20150	flagellar hook-associated protein FlgK	-4.43	6.65E-22
DR76_RS20155	flagellar rod assembly protein FlgJ	-1.09	7.02E-03
DR76_RS20160	flagellar basal body P-ring protein	-1.55	1.26E-03
DR76_RS20175	flagellar basal body rod protein FlgF	-2.35	2.02E-05
DR76_RS20180	flagellar hook protein FlgE	-3.36	5.81E-09
DR76_RS20185	flagellar basal body rod modification protein	-3.47	3.83E-09
DR76_RS20190	flagellar component of cell-proximal portion of basal-body rod	-3.60	7.53E-08
DR76_RS20195	flagellar component of cell-proximal portion of basal-body rod	-4.07	1.22E-07
DR76_RS20205	anti-sigma28 factor FlgM	-3.73	1.10E-17
DR76_RS20210	flagella synthesis chaperone protein FlgN	-4.02	1.53E-15
DR76_RS20255	DNA damage-inducible protein I	-2.47	5.60E-09
DR76_RS20270	hypothetical protein	-2.37	1.10E-03

DR76_RS20360	major curlin subunit	-2.86	8.81E-07
DR76_RS20425	hypothetical protein	-7.80	6.18E-25
DR76_RS20430	phage protein	-3.48	2.71E-11
DR76_RS20435	toxin	-3.67	8.86E-05
DR76_RS20440	antitoxin YeeU	-16.84	3.70E-15
DR76_RS20445	hypothetical protein	-5.22	1.30E-15
DR76_RS20450	hypothetical protein	-16.84	5.47E-07
DR76_RS20455	phage DNA repair protein	-16.84	1.50E-10
DR76_RS20460	hypothetical protein	-16.84	1.81E-23
DR76_RS20465	hypothetical protein	-16.84	2.06E-34
DR76_RS20470	hypothetical protein	-16.84	4.28E-20
DR76_RS20475	hypothetical protein	-16.84	5.80E-19
DR76_RS20480	membrane protein	-16.84	6.71E-31
DR76_RS20485	hypothetical protein	-10.07	9.94E-45
DR76_RS20490	hypothetical protein	-16.84	1.23E-13
DR76_RS20495	hypothetical protein	-16.84	1.46E-07
DR76_RS20500	hypothetical protein	-16.84	1.64E-26
DR76_RS20505	hypothetical protein	-16.84	1.14E-05
DR76_RS20510	hypothetical protein	-16.84	2.15E-20
DR76_RS20515	hypothetical protein	-16.84	6.24E-49
DR76_RS20520	transposase	-16.84	1.10E-09
DR76_RS20525	hypothetical protein	-16.84	4.71E-24
DR76_RS20530	ABC transporter	-16.84	1.03E-41
DR76_RS20535	ligand-gated channel	-11.08	5.63E-43
DR76_RS20540	hypothetical protein	-10.74	2.30E-41
DR76_RS20545	hypothetical protein	-16.84	2.05E-49
DR76_RS20550	membrane protein	-16.84	8.23E-30
DR76_RS20555	membrane protein	-16.84	5.02E-25
DR76_RS20560	transposase	-16.84	7.76E-30
DR76_RS20565	transposase	-16.84	4.49E-22
DR76_RS20570	hypothetical protein	-16.84	2.82E-19
DR76_RS20575	glycosyl transferase	-16.84	9.12E-33
DR76_RS20580	transporter	-16.84	1.62E-55
DR76_RS20585	enterochelin esterase	-10.15	4.31E-38
DR76_RS20590	hypothetical protein	-16.84	2.63E-31
DR76_RS20595	outer membrane receptor protein	-16.84	2.89E-42
DR76_RS20600	DDE endonuclease	-16.84	9.37E-38
DR76_RS20605	transcriptional regulator	-16.84	2.80E-28
DR76_RS20610	diguanylate phosphodiesterase	-16.84	8.01E-33
DR76_RS20615	S-fimbrial protein subunit SfaH	-16.84	2.50E-31
DR76_RS20620	S-fimbrial adhesin protein SfaS	-16.84	3.66E-25
DR76_RS20625	S-fimbrial protein subunit SfaG	-16.84	7.29E-32
DR76_RS20630	fimbrial protein FimD	-16.84	2.12E-43
DR76_RS20635	molecular chaperone FimC	-16.84	3.91E-38

DR76_RS20640	fimbrin fimI	-16.84	3.11E-41
DR76_RS20645	type-1 fimbrial protein subunit A	-14.34	2.65E-38
DR76_RS20650	Major pilu subunit operon regulatory protein papB	-16.84	1.85E-42
DR76_RS20655	Major pilus subunit operon regulatory protein	-16.84	1.36E-22
DR76_RS20660	hypothetical protein	-16.84	1.61E-30
DR76_RS20665	hypothetical protein	-16.84	7.25E-24
DR76_RS20670	hypothetical protein	-16.84	1.57E-06
DR76_RS20675	hypothetical protein	-16.84	3.47E-13
DR76_RS20680	CAAX amino terminal protease	-16.84	5.43E-15
DR76_RS20685	hypothetical protein	-16.84	2.91E-11
DR76_RS20690	colicin V synthesis protein	-16.84	6.86E-25
DR76_RS20695	colicin V secretion protein CvaA	-16.84	4.60E-22
DR76_RS20700	RTX toxin acyltransferase	-16.84	5.16E-21
DR76_RS20705	hypothetical protein	-16.84	5.47E-34
DR76_RS20710	microcin H47 immunity protein mchI	-16.84	5.45E-31
DR76_RS20715	membrane protein	-16.84	9.62E-09
DR76_RS20720	membrane protein	-16.84	2.37E-17
DR76_RS20725	transposase	-16.84	3.61E-23
DR76_RS20730	phospho-2-dehydro-3-deoxyheptonate aldolase	-16.84	3.82E-24
DR76_RS20740	transposase	-2.98	6.12E-05
DR76_RS20745	hypothetical protein	-16.84	2.12E-24
DR76_RS20750	hypothetical protein	-16.84	2.72E-39
DR76_RS20755	hypothetical protein	-16.84	2.37E-61
DR76_RS20760	3'-5' exonuclease	-16.84	1.15E-52
DR76_RS20765	integrase	-16.84	6.48E-44
DR76_RS20770	hypothetical protein	-16.84	6.04E-25
DR76_RS20775	hypothetical protein	-16.84	3.08E-21
DR76_RS20785	hypothetical protein	-16.84	4.44E-10
DR76_RS20795	hemagglutinin	-16.84	4.48E-29
DR76_RS20800	adhesin HecA family 20-residue repeat (two copies)	-16.84	1.28E-53
DR76_RS20805	hypothetical protein	-16.84	1.21E-61
DR76_RS20810	O-methyltransferase	-16.84	1.03E-50
DR76_RS20815	hypothetical protein	-16.84	2.59E-41
DR76_RS20820	1-acyl-sn-glycerol-3-phosphate acyltransferase	-16.84	7.87E-42
DR76_RS20825	acyl carrier protein	-16.84	4.23E-44
DR76_RS20830	acyl carrier protein	-16.84	1.44E-39
DR76_RS20835	membrane protein	-16.84	3.73E-42
DR76_RS20840	AMP-dependent synthetase	-16.84	4.42E-59
DR76_RS20845	hydroxymyristoyl-ACP dehydratase	-16.84	1.73E-33
DR76_RS20850	acyltransferase	-16.84	2.83E-57
DR76_RS20855	hypothetical protein	-16.84	7.15E-33
DR76_RS20860	membrane protein	-16.84	1.20E-39
DR76_RS20865	membrane protein	-16.84	9.68E-46
DR76_RS20870	hypothetical protein	-16.84	8.25E-28

DR76_RS20875	3-oxoacyl-ACP synthase	-16.84	1.01E-29
DR76_RS20880	3-hydroxy-fatty acyl-ACP dehydratase	-16.84	4.84E-22
DR76_RS20885	3-ketoacyl-ACP reductase	-16.84	1.74E-22
DR76_RS20890	3-oxoacyl-ACP synthase	-16.84	1.17E-37
DR76_RS20895	hypothetical protein	-16.84	3.56E-27
DR76_RS20900	hypothetical protein	-16.84	4.11E-13
DR76_RS20905	hydrolase	-16.84	2.58E-09
DR76_RS20910	galactarate dehydratase	-16.84	2.87E-17
DR76_RS20915	deoR C terminal sensor domain protein	-5.22	7.98E-12
DR76_RS20925	tagatose-6-phosphate ketose isomerase	-16.84	1.32E-34
DR76_RS20930	aminotransferase	-16.84	2.49E-45
DR76_RS20935	enterotoxin	-16.84	7.97E-48
DR76_RS20940	methyltransferase	-16.84	3.13E-14
DR76_RS20945	hypothetical protein	-1.76	3.05E-03
DR76_RS20950	hypothetical protein	-16.84	7.00E-27
DR76_RS20955	hypothetical protein	-16.84	6.23E-07
DR76_RS20960	regulatory protein	-16.84	1.43E-22
DR76_RS20965	membrane protein	-16.84	6.26E-21
DR76_RS20970	membrane protein	-16.84	2.55E-53
DR76_RS20980	hypothetical protein	-4.14	2.40E-10
DR76_RS21270	methylglyoxal synthase	-1.15	1.70E-03
DR76_RS21295	SOS cell division inhibitor	-3.11	4.33E-08
DR76_RS21300	hypothetical protein	-3.26	4.09E-14
DR76_RS21370	NAD(P)H-dependent FMN reductase	-1.22	1.49E-02
DR76_RS21380	alkanesulfonate monooxygenase	-1.23	7.90E-03
DR76_RS21390	aliphatic sulfonate ABC transporter ATP-binding protein	-1.33	1.15E-03
DR76_RS21410	outer membrane phosphoporin protein E	-3.71	1.69E-10
DR76_RS21420	aromatic amino acid aminotransferase	-1.37	8.54E-03
DR76_RS21515	metalloprotease	-1.37	7.81E-04
DR76_RS21535	hypothetical protein	-1.69	3.85E-05
DR76_RS21545	formate C-acetyltransferase 1-2C anaerobic-3B pyruvate formate-lyase 1	-1.43	1.96E-02
DR76_RS21570	dimethyl sulfoxide reductase subunit B	-1.39	1.97E-04
DR76_RS21605	thioredoxin reductase	-1.24	7.07E-03
DR76_RS21665	hypothetical protein	-1.47	1.52E-04
DR76_RS21685	hydroxylamine reductase	-3.99	3.09E-05
DR76_RS21690	oxidoreductase	-3.32	3.87E-04
DR76_RS21835	sugar phosphatase SupH	-1.07	6.41E-03
DR76_RS21935	pyruvate formate-lyase 3-activating protein	-1.05	1.26E-02
DR76_RS22070	hypothetical protein	-5.70	1.10E-19
DR76_RS22080	transcriptional regulator	-1.12	1.29E-02
DR76_RS22085	transporter	-1.94	6.68E-04
DR76_RS22090	multidrug ABC transporter ATP-binding protein	-1.40	1.71E-02
DR76_RS22150	molybdenum cofactor biosynthesis protein B	-1.27	8.28E-04
DR76_RS22175	dithiobiotin synthetase	-2.17	4.67E-07

DR76_RS22180	biotin biosynthesis protein BioC	-1.60	1.18E-04
DR76_RS22185	8-amino-7-oxononanoate synthase	-2.77	4.55E-10
DR76_RS22190	biotin synthase	-2.74	2.59E-11
DR76_RS22195	adenosylmethionine--8-amino-7-oxononanoate aminotransferase	-1.23	1.49E-03
DR76_RS22200	transposase	-16.84	3.02E-58
DR76_RS22280	UDP-galactose-4-epimerase	-2.56	1.28E-05
DR76_RS22285	galactose-1-phosphate uridylyltransferase	-2.81	9.44E-07
DR76_RS22290	galactokinase	-2.26	6.50E-05
DR76_RS22340	membrane protein	-1.49	4.75E-04
DR76_RS22345	hypothetical protein	-3.79	2.54E-07
DR76_RS22350	membrane protein	-1.41	2.20E-03
DR76_RS22355	hypothetical protein	-5.81	4.06E-17
DR76_RS22360	hypothetical protein	-1.78	2.70E-03
DR76_RS22365	membrane protein	-1.59	4.94E-05
DR76_RS22425	hypothetical protein	-16.84	1.71E-02
DR76_RS22430	hypothetical protein	-16.84	1.28E-15
DR76_RS22455	membrane protein	-4.83	5.68E-10
DR76_RS22490	hypothetical protein	-5.40	1.43E-15
DR76_RS22535	hypothetical protein	-1.27	5.02E-03
DR76_RS22540	hypothetical protein	-2.96	2.29E-12
DR76_RS22545	potassium transporter TrkA	-16.84	1.75E-03
DR76_RS22550	potassium-transporting ATPase subunit A	-4.00	7.78E-15
DR76_RS22580	ornithine decarboxylase-2C inducible	-8.81	2.74E-09
DR76_RS22585	putrescine-ornithine antiporter	-5.73	1.49E-09
DR76_RS22635	chitoporin	-1.15	1.63E-03
DR76_RS22645	DeoR family transcriptional regulator	-16.84	5.66E-44
DR76_RS22650	4-hydroxythreonine-4-phosphate dehydrogenase	-16.84	6.27E-22
DR76_RS22655	membrane protein	-16.84	4.20E-23
DR76_RS22660	DhaT	-16.84	1.66E-25
DR76_RS22665	dihydrodipicolinate synthetase	-16.84	6.59E-19
DR76_RS22670	membrane protein	-16.84	4.73E-06
DR76_RS22675	glycosyl hydrolase	-16.84	2.09E-12
DR76_RS22680	hypothetical protein	-16.84	2.22E-08
DR76_RS22685	hypothetical protein	-16.84	2.93E-24
DR76_RS22690	PTS N-acetylglucosamine transporter subunit IIABC	-2.01	1.47E-05
DR76_RS22810	ribonucleoside hydrolase	-2.19	1.85E-05
DR76_RS22890	hypothetical protein	-2.04	9.91E-04
DR76_RS22925	C4-dicarboxylate ABC transporter	-2.95	6.61E-08
DR76_RS22930	transcriptional regulatory protein DpiA	-1.25	8.35E-04
DR76_RS22945	citrate lyase subunit gamma	-2.73	1.43E-03
DR76_RS22950	citrate lyase-2C citryl-ACP lyase (beta) subunit	-2.72	6.87E-05
DR76_RS22955	citrate lyase subunit alpha	-3.51	9.22E-06
DR76_RS22960	2-(5"-triphosphoribosyl)-3'-dephospho-CoA synthase	-3.34	1.04E-04
DR76_RS22985	universal stress protein G	-2.37	9.72E-06

DR76_RS23005	LysR family transcripitonal regulator	-5.38	2.13E-12
DR76_RS23090	hypothetical protein	-9.03	9.41E-30
DR76_RS23095	enterobactin synthase subunit F	-1.05	3.30E-03
DR76_RS23120	4'-phosphopantetheinyl transferase	-1.36	1.84E-03
DR76_RS23125	membrane protein	-1.71	2.46E-05
DR76_RS23130	hypothetical protein	-2.70	9.01E-10
DR76_RS23135	ubiquitin carboxyl-hydrolase	-3.29	5.60E-05
DR76_RS23175	metal RND transporter	-1.31	5.78E-03
DR76_RS23180	cation transporter	-1.65	1.34E-02
DR76_RS23185	cation efflux system protein CusC	-1.88	1.47E-03
DR76_RS23215	protease	-16.84	4.52E-30
DR76_RS23220	hypothetical protein	-16.84	7.39E-23
DR76_RS23225	tail fiber assembly protein	-16.84	3.86E-18
DR76_RS23230	integrase	-16.84	7.12E-12
DR76_RS23235	integrase	-16.84	3.02E-21
DR76_RS23245	hypothetical protein	-16.84	6.93E-08
DR76_RS23280	hypothetical protein	-6.99	3.33E-12
DR76_RS23310	membrane protein	-1.16	2.02E-02
DR76_RS23315	ureidoglycolate dehydrogenase	-2.24	1.86E-04
DR76_RS23320	allantoate amidohydrolase	-1.19	8.37E-03
DR76_RS23340	allantoinase	-1.18	7.72E-03
DR76_RS23370	ureidoglycolate hydrolase	-1.17	1.06E-02
DR76_RS23375	LysR family transcriptional regulator	-1.38	9.60E-04
DR76_RS23430	hypothetical protein	-16.84	1.17E-43
DR76_RS23455	putative DNA-binding transcriptional regulator	-1.16	1.23E-03
DR76_RS23660	hypothetical protein	-1.21	2.22E-03
DR76_RS23670	HU-2C DNA-binding transcriptional regulator-2C beta subunit	-1.19	1.68E-02
DR76_RS23820	ion channel protein Tsx	-1.41	8.73E-03
DR76_RS23825	hypothetical protein	-1.53	1.11E-05
DR76_RS23965	anti-RssB factor	-1.29	4.20E-04
DR76_RS24015	transposase	-16.84	2.94E-04
DR76_RS24025	transposase	-16.84	8.50E-09
DR76_RS24060	hypothetical protein	-4.15	4.53E-05
DR76_RS24165	hypothetical protein	-1.04	1.35E-02
DR76_RS24185	hypothetical protein	-2.02	9.09E-03
DR76_RS24250	hypothetical protein	-6.61	1.11E-13
DR76_RS24255	DNA recombinase	-16.84	1.89E-28
DR76_RS24260	LuxR family transcriptional regulator	-16.84	8.01E-24
DR76_RS24265	hypothetical protein	-16.84	3.04E-16
DR76_RS24270	autotransporter	-16.84	2.20E-35
DR76_RS24280	universal stress protein	-4.32	7.07E-05
DR76_RS24285	hypothetical protein	-3.94	3.47E-07
DR76_RS24290	amino acid dehydrogenase	-3.49	3.59E-06
DR76_RS24295	hypothetical protein	-3.78	1.07E-08

DR76_RS24300	AraC family transcriptional regulator	-1.13	1.14E-02
DR76_RS24320	aldo/keto reductase	-7.35	2.72E-28
DR76_RS24335	2-2C5-diketo-D-gluconic acid reductase	-16.84	7.52E-41
DR76_RS24340	aldo/keto reductase	-16.84	1.29E-45
DR76_RS24345	transcriptional regulator	-16.84	2.73E-45
DR76_RS24350	transcriptional regulator	-16.84	1.41E-40
DR76_RS24355	alpha/beta hydrolase	-16.84	5.34E-50
DR76_RS24360	NADH-dependent flavin oxidoreductase	-8.96	7.92E-37
DR76_RS24365	uncharacterized protein	-4.20	5.26E-12
DR76_RS24380	LuxR family transcriptional regulator	-2.68	3.78E-07
DR76_RS24385	fimbrillin MatB	-1.79	7.90E-07
DR76_RS24390	hypothetical protein	-1.89	1.75E-05
DR76_RS24395	hypothetical protein	-1.19	7.16E-04
DR76_RS24415	membrane protein	-2.22	8.87E-09
DR76_RS24420	membrane protein	-16.84	6.21E-11
DR76_RS24425	transposase	-16.84	3.24E-16
DR76_RS24435	membrane protein	-16.84	7.72E-09
DR76_RS24440	transposase	-16.84	5.08E-15
DR76_RS24445	peptidase	-16.84	8.40E-55
DR76_RS24450	transcriptional regulator	-16.84	1.83E-03
DR76_RS24455	integrase	-16.84	6.21E-19
DR76_RS24500	peptide chain release factor	-2.94	7.38E-13
DR76_RS24505	hypothetical protein	-5.74	1.07E-23
DR76_RS24510	acyltransferase	-1.53	5.72E-04
DR76_RS24515	DNA polymerase IV	-1.64	5.53E-05
DR76_RS24530	endopeptidase	-1.32	6.49E-03
DR76_RS24565	membrane protein	-16.84	1.97E-05
DR76_RS24575	membrane protein	-16.84	1.45E-22
DR76_RS24580	LuxR family transcriptional regulator	-16.84	8.62E-05
DR76_RS24585	RTX family exoprotein A protein	-16.84	2.89E-47
DR76_RS24590	hemolysin secretion protein D	-16.84	2.67E-25
DR76_RS24595	membrane protein	-16.84	1.53E-29
DR76_RS24600	transporter	-16.84	2.07E-24
DR76_RS24605	transposase	-3.47	1.74E-09
DR76_RS24610	transposase	-16.84	5.11E-19
DR76_RS24615	autotransporter	-12.61	3.66E-58
DR76_RS24620	transposase	-16.84	5.30E-13
DR76_RS24625	membrane protein	-16.84	8.55E-27
DR76_RS24630	hypothetical protein	-16.84	1.39E-10
DR76_RS24645	Tat pathway signal sequence protein	-16.84	5.18E-06
DR76_RS24655	Contact-dependent inhibitor A	-16.84	5.18E-59
DR76_RS24660	membrane protein	-16.84	1.67E-60
DR76_RS24665	DNA-binding protein	-16.84	7.15E-31
DR76_RS24670	LacI family transcription regulator	-16.84	7.60E-47

DR76_RS24675	PTS ascorbate transporter subunit IIA	-16.84	1.67E-18
DR76_RS24680	PTS ascorbate transporter subunit IIB	-16.84	1.14E-20
DR76_RS24685	PTS beta-glucoside transporter subunit IIBC	-16.84	5.88E-39
DR76_RS24690	membrane protein	-16.84	2.65E-25
DR76_RS24695	deoxyribose mutarotase	-16.84	8.32E-44
DR76_RS24700	sugar:proton symporter	-16.84	1.21E-31
DR76_RS24705	ribokinase	-16.84	2.39E-28
DR76_RS24710	DeoR family transcriptional regulator	-16.84	1.07E-27
DR76_RS24720	acetolactate synthase	-5.04	1.97E-06
DR76_RS24725	hypothetical protein	-16.84	1.97E-05
DR76_RS24730	membrane protein	-16.84	1.99E-23
DR76_RS24735	CAAX amino terminal protease	-16.84	5.65E-05
DR76_RS24740	hypothetical protein	-16.84	1.34E-07
DR76_RS24745	exopolygalacturonate lyase	-16.84	2.62E-31
DR76_RS24750	major facilitator transporter	-16.84	5.03E-20
DR76_RS24755	cupin	-16.84	5.64E-18
DR76_RS24760	gluconate 5-dehydrogenase	-16.84	6.08E-25
DR76_RS24765	oligogalacturonate lyase	-16.84	4.84E-34
DR76_RS24770	hypothetical protein	-16.84	5.98E-25
DR76_RS24775	regulator	-16.84	2.07E-08
DR76_RS24780	hypothetical protein	-16.84	4.08E-14
DR76_RS24785	hypothetical protein	-16.84	3.52E-31
DR76_RS24790	hypothetical protein	-16.84	4.82E-17
DR76_RS24795	hypothetical protein	-16.84	1.80E-06
DR76_RS24800	hypothetical protein	-16.84	4.93E-31
DR76_RS24805	hemolysin activation protein	-16.84	9.65E-12
DR76_RS24810	hypothetical protein	-16.84	4.64E-32
DR76_RS24815	Rha family transcriptional regulator	-16.84	4.68E-06
DR76_RS24820	hypothetical protein	-16.84	3.82E-06
DR76_RS24825	hypothetical protein	-16.84	2.48E-02
DR76_RS24830	hypothetical protein	-16.84	4.11E-20
DR76_RS24835	hypothetical protein	-16.84	4.82E-04
DR76_RS24860	hypothetical protein	-16.84	3.35E-42
DR76_RS24870	resolvase	-16.84	3.65E-30
DR76_RS24875	hypothetical protein	-16.84	8.08E-65
DR76_RS24880	hypothetical protein	-16.84	3.33E-11
DR76_RS24885	hypothetical protein	-16.84	4.69E-25
DR76_RS24890	chemotaxis protein	-16.84	4.64E-18
DR76_RS24895	phospholipase	-16.84	9.12E-26
DR76_RS24900	hypothetical protein	-16.84	4.04E-22
DR76_RS24905	hypothetical protein	-16.84	5.04E-28
DR76_RS24910	hypothetical protein	-4.59	6.59E-12
DR76_RS24915	hypothetical protein	-16.84	3.76E-15
DR76_RS24920	hypothetical protein	-16.84	5.38E-20

DR76_RS24925	CP4-44 prophage-3B uncharacterized protein	-16.84	2.11E-15
DR76_RS24930	antitoxin	-16.84	1.02E-15
DR76_RS24935	CP4-44 prophage-3B toxin of the YeeV-YeeU toxin-antitoxin system	-16.84	1.53E-11
DR76_RS24940	hypothetical protein	-16.84	2.89E-09
DR76_RS24945	hypothetical protein	-16.84	5.27E-14
DR76_RS24970	hypothetical protein	-16.84	2.08E-20
DR76_RS24980	membrane protein	-16.84	4.73E-05
DR76_RS25010	toxin YoeB	-1.46	2.63E-04
DR76_RS25015	antitoxin of the YoeB-YefM toxin-antitoxin system	-1.30	1.10E-03
DR76_RS25025	bifunctional histidinal dehydrogenase/ histidinol dehydrogenase	-1.23	3.39E-04
DR76_RS25045	1-(5-phosphoribosyl)-5-[(5-phosphoribosylamino)methylideneamino] imidazole-4-carboxamide isomerase	-1.09	2.02E-03
DR76_RS25055	phosphoribosyl-AMP cyclohydrolase	-1.86	1.42E-06
DR76_RS25060	chain-length determining protein	-5.90	5.12E-21
DR76_RS25070	6-phosphogluconate dehydrogenase	-2.85	5.49E-07
DR76_RS25075	phosphomannomutase	-10.18	2.90E-29
DR76_RS25080	mannose-1-phosphate guanyltransferase	-16.84	1.77E-33
DR76_RS25085	glycosyl transferase family 1	-16.84	3.71E-32
DR76_RS25090	UDP-glucose 4-epimerase	-16.84	1.47E-36
DR76_RS25095	glycosyl transferase family 1	-16.84	1.49E-31
DR76_RS25100	glycosyl transferase family 1	-16.84	2.19E-47
DR76_RS25105	glycosyl transferase family A	-16.84	7.09E-50
DR76_RS25110	polymerase	-16.84	7.53E-55
DR76_RS25115	polysaccharide biosynthesis protein	-16.84	8.21E-53
DR76_RS25140	colanic acid exporter	-1.76	7.49E-05
DR76_RS25150	phosphomannomutase	-1.43	7.79E-03
DR76_RS25180	acyl transferase	-1.42	2.34E-02
DR76_RS25265	hypothetical protein	-1.28	2.76E-03
DR76_RS25270	hypothetical protein	-2.58	6.67E-06
DR76_RS25275	multidrug transporter	-1.32	3.92E-04
DR76_RS25310	hypothetical protein	-16.84	2.02E-25
DR76_RS25315	membrane protein	-16.84	6.70E-24
DR76_RS25320	membrane protein	-16.84	1.77E-15
DR76_RS25325	hypothetical protein	-16.84	2.16E-13
DR76_RS25410	membrane protein	-1.86	3.41E-04
DR76_RS25415	tail protein	-16.84	9.00E-15
DR76_RS25420	hypothetical protein	-16.84	2.12E-18
DR76_RS25425	hypothetical protein	-16.84	1.23E-36
DR76_RS25430	hypothetical protein	-16.84	8.54E-18
DR76_RS25435	baseplate assembly protein	-16.84	1.51E-13
DR76_RS25440	hypothetical protein	-16.84	3.06E-06
DR76_RS25445	phage baseplate protein	-16.84	1.40E-17
DR76_RS25450	Tail protein I	-16.84	6.02E-11
DR76_RS25455	hypothetical protein	-16.84	3.16E-16

DR76_RS25475	tail fiber protein	-16.84	2.14E-10
DR76_RS25480	multiple promoter invertase	-16.84	7.69E-31
DR76_RS25485	hypothetical protein	-16.84	1.17E-43
DR76_RS25490	hypothetical protein	-16.84	2.01E-34
DR76_RS25495	hypothetical protein	-16.84	1.98E-32
DR76_RS25500	toxin RelE	-13.78	3.11E-56
DR76_RS25505	transcriptional regulator	-16.84	1.66E-62
DR76_RS25510	hypothetical protein	-16.84	1.26E-41
DR76_RS25515	hypothetical protein	-16.84	5.93E-27
DR76_RS25520	hypothetical protein	-16.84	1.71E-18
DR76_RS25525	hypothetical protein	-16.84	2.90E-44
DR76_RS25530	hypothetical protein	-16.84	6.88E-24
DR76_RS25535	hypothetical protein	-12.78	6.44E-58
DR76_RS25540	hypothetical protein	-16.84	1.01E-42
DR76_RS25545	ATPase	-16.84	4.43E-46
DR76_RS25550	hypothetical protein	-16.84	2.96E-20
DR76_RS25555	hypothetical protein	-16.84	1.22E-54
DR76_RS25560	hypothetical protein	-16.84	2.39E-29
DR76_RS25565	hypothetical protein	-16.84	1.08E-23
DR76_RS25570	hypothetical protein	-16.84	9.86E-14
DR76_RS25575	hypothetical protein	-16.84	4.62E-17
DR76_RS25580	hypothetical protein	-16.84	2.11E-17
DR76_RS25585	hypothetical protein	-16.84	2.08E-20
DR76_RS25590	hypothetical protein	-16.84	1.22E-24
DR76_RS25595	hypothetical protein	-16.84	4.87E-20
DR76_RS25600	hypothetical protein	-16.84	5.90E-23
DR76_RS25605	hypothetical protein	-16.84	5.58E-23
DR76_RS25610	hypothetical protein	-16.84	5.33E-24
DR76_RS25615	hypothetical protein	-16.84	6.93E-19
DR76_RS25620	membrane protein	-16.84	7.70E-20
DR76_RS25625	membrane protein	-16.84	3.14E-13
DR76_RS25630	hypothetical protein	-16.84	1.59E-21
DR76_RS25635	hypothetical protein	-16.84	1.61E-18
DR76_RS25640	-	-16.84	2.58E-02
DR76_RS25645	hypothetical protein	-16.84	1.68E-18
DR76_RS25650	hypothetical protein	-16.84	3.03E-19
DR76_RS25655	hypothetical protein	-16.84	1.76E-18
DR76_RS25660	hypothetical protein	-16.84	4.52E-20
DR76_RS25665	hypothetical protein	-16.84	2.05E-16
DR76_RS25670	hypothetical protein	-16.84	4.17E-28
DR76_RS25675	transcriptional regulator	-16.84	1.64E-25
DR76_RS25680	hypothetical protein	-16.84	6.81E-39
DR76_RS25685	hypothetical protein	-16.84	4.15E-28
DR76_RS25690	nuclease	-16.84	4.00E-15

DR76_RS25695	hypothetical protein	-16.84	2.01E-19
DR76_RS25700	hypothetical protein	-16.84	2.21E-28
DR76_RS25705	hypothetical protein	-16.84	7.04E-10
DR76_RS25710	portal protein	-16.84	2.86E-28
DR76_RS25715	protease	-16.84	7.35E-26
DR76_RS25720	hypothetical protein	-16.84	1.64E-16
DR76_RS25725	capsid protein	-16.84	1.79E-32
DR76_RS25730	hypothetical protein	-16.84	7.89E-20
DR76_RS25735	hypothetical protein	-16.84	2.42E-11
DR76_RS25740	hypothetical protein	-16.84	5.97E-14
DR76_RS25745	hypothetical protein	-16.84	5.19E-07
DR76_RS25750	phage tail protein	-16.84	1.99E-27
DR76_RS25755	membrane protein	-16.84	2.50E-18
DR76_RS25760	hypothetical protein	-16.84	2.47E-41
DR76_RS25765	hypothetical protein	-16.84	1.83E-46
DR76_RS25770	peptidyl-arginine deiminase	-16.84	5.08E-60
DR76_RS25780	hypothetical protein	-16.84	4.17E-22
DR76_RS25785	hypothetical protein	-16.84	2.82E-46
DR76_RS25790	hypothetical protein	-16.84	1.07E-50
DR76_RS25795	hypothetical protein	-16.84	1.54E-34
DR76_RS25800	membrane protein	-16.84	7.31E-40
DR76_RS25805	membrane protein	-15.42	3.41E-51
DR76_RS25810	peptidase S66	-16.84	2.02E-53
DR76_RS25815	hypothetical protein	-16.84	3.63E-25
DR76_RS25820	hypothetical protein	-16.84	9.50E-22
DR76_RS25825	hypothetical protein	-16.84	1.50E-39
DR76_RS25830	SagC family bacteriocin biosynthesis cyclodehydratase	-16.84	5.97E-51
DR76_RS25835	hypothetical protein	-16.84	2.57E-29
DR76_RS25840	hypothetical protein	-16.84	4.41E-51
DR76_RS25845	DNA-binding protein	-11.27	9.49E-47
DR76_RS25850	hypothetical protein	-16.84	2.28E-32
DR76_RS25855	hypothetical protein	-16.84	2.52E-32
DR76_RS25860	hypothetical protein	-16.84	6.26E-41
DR76_RS25865	hypothetical protein	-16.84	5.50E-43
DR76_RS25870	hypothetical protein	-16.84	1.28E-48
DR76_RS25875	toxin RelE	-1.23	3.57E-03
DR76_RS25885	hypothetical protein	-1.20	3.37E-03
DR76_RS25895	hypothetical protein	-2.64	1.06E-11
DR76_RS25900	hypothetical protein	-5.27	1.01E-19

Supplementary Table 3: Oligonucleotides used for PCR amplification of resistant genes in poultry *E. coli*

Resistant genes	Primer part	Primer gene sequence (5´ to3´)	References
aac(3)-IV	aac(3)-IV-F	CTTCAGGATGGCAAGTTGGT	17
	aac(3)-IV -R	TCATCTCGTTCTCCGCTCAT	
cat-A1	cat-A1-F	AGTTGCTCAATGTACCTATATAACC	17
	cat-A1-R	TTGTAATTCATTAAGCATTCTGCC	
cml-A	cml-A-F	CCGCCACGGTGTTGTTGTTATC	
	cml-A-R	CACCTTGCCTGCCCATCATTAG	
qnr-A	qnr-A-F	AGAGGATTTCTCACGCCAGG	20
	qnr-A-R	TGCCAGGCACAGATCTTGAC	
qnr-B	qnr-B-F	GGMATHGAAATTCGCCACTG	21
	qnr-B-R	TTTGCYGYYCGCCAGTCGAA	
qnr-S	qnr-S-F	ACGACATTCGTCAACTGCAA	22
	qnr-S-R	TAAATTGGCACCCTGTAGGC	
tet-A	tet-A-F	TTGGCATTCTGCATTCACTCG	16
	tet-A-R	CCACCCGTTCCACGTTGTT	
tet-B	tet-B-F	TTCACCGCATAGTCCCTT	
	tet-B-R	TGCAATAAATCCGAGCAG	
CTX-M	CTX-M F	CGCTTTGCGATGTGCAG	23
	CTX-M R	ACCGCGATATCGTTGGT	
CTX-M-1	CTX-M-1 F	GCTGTTGTTAGGAAGTGTGCCGC	24
	CTX-M-1 R	GCCGCCGACGCTAATACATC	
OXY	OXY F	GGTTTTGGTAACTGTGACGGG	25
	OXY R	CAGAGTGCAGAGTGTTGCAG	
TEM-1	TEM-1 F	ATAAAATTCTTGAAGACGAAA	
	TEM-1 R	GACAGTTACCAATGCTTAATC	
MdtB	*MdtB-F*	TCTTCCCGGTACAGGACAAT	18
	MdtB-R	CATCAACGCCAACAAATGAG	
MdtF	*MdtF-F*	CCGTACCGGTGGTTATTCTC	
	MdtF-R	ATCGATTTATGCGTCGCTTC	
MdtG	*MdtG*-F	CGGTATTGTCTTCAGCATTACATTTT	19
	MdtG-F	GGCGAGTCCACCCCAAA	
MdtL	*MdtL*-F	TATCCCGCCGGGATTGATAT	
	MdtL-R	CGCTTCGCTGGCATTGA	

Supplementary Table 4: Primers used in RT-qPCR

Gene ID	Gene name	Gene Description	Direc-tion	Sequence	bp
DR76_RS09810	NlpD	Lipoprotein NlpD	F	ACGATTTCCGTGAC CTTGCT	236
			R	AGCACTCTGTTCAC CCGAAG	
DR76_RS21875	BssR	Biofilm formation regulatory protein BssR	F	ACGGTAATAACAGT CGGGCA	196
			R	ACGGTAATAACAGT CGGGCA	
DR76_RS23570	Hha	Hha toxicity attenuator B conjugation-related protein	F	GGGTTAACGACCCA ACCTCG	199
			R	TTCCGCCATTTCTGA AGATCC	
DR76_RS05060	MdtF	Multidrug resistance protein MdtF	F	CCGTCACCTGTTGTA GCACT	277
			R	GAGGGCGGTCACAA ACCTAA	
DR76_RS20295	MdtG	Multidrug resistance protein MdtG	F	TACTTGGCGGATTTG TCCCC	193
			R	ATGAGCACACTGGC GGTAAT	
DR76_RS12580	FimC	Molecular chaperone FimC	F	CGTTTTATCGTGACG CCTCC	253
			R	ATTTTTCTGCGGCCT GATCG	
DR76_RS09375	ImpG	Type VI secretion protein ImpG	F	TACCCGCGTGTGGT AATAGC	216
			R	CTCGAAGCCGATCC GTTACA	
DR76_RS07265	RelE	Toxin RelE	F	GTCTGCCCAGTTCTG GAATC	158
			R	CCTTAGTACGATGA ACGGCG	
DR76_RS17090	CspB	Cold shock-like protein CspB	F	CGCTGATAAAGGTT TCGGCT	150
			R	GCAGGACCTTTAGC ACCACT	
DR76_RS14030	dnaE	DNA polymerase III subunit alpha	F	GATTGAGCGTTATG TCGGAGGC	81
			R	GCCCCGCAGCCGTG AT	
